# Synthesis, Photophysical and Electronic Properties of New Red‐to‐NIR Emitting Donor–Acceptor Pyrene Derivatives

**DOI:** 10.1002/chem.201904219

**Published:** 2019-11-19

**Authors:** Julia Merz, Maximilian Dietz, Yvonne Vonhausen, Frederik Wöber, Alexandra Friedrich, Daniel Sieh, Ivo Krummenacher, Holger Braunschweig, Michael Moos, Marco Holzapfel, Christoph Lambert, Todd B. Marder

**Affiliations:** ^1^ Institut für Anorganische Chemie and Institute for Sustainable Chemistry & Catalysis with Boron (ICB) Julius-Maximilians-Universität Würzburg Am Hubland 97074 Würzburg Germany; ^2^ Institut für Organische Chemie Julius-Maximilians-Universität Würzburg Am Hubland 97074 Würzburg Germany

**Keywords:** borylation, K-region, luminescence, polycyclic aromatic hydrocarbons, redox

## Abstract

We synthesized new pyrene derivatives with strong bis(*para*‐methoxyphenyl)amine donors at the 2,7‐positions and *n*‐azaacene acceptors at the K‐region of pyrene. The compounds possess a strong intramolecular charge transfer, leading to unusual properties such as emission in the red to NIR region (700 nm), which has not been reported before for monomeric pyrenes. Detailed photophysical studies reveal very long intrinsic lifetimes of >100 ns for the new compounds, which is typical for 2,7‐substituted pyrenes but not for K‐region substituted pyrenes. The incorporation of strong donors and acceptors leads to very low reduction and oxidation potentials, and spectroelectrochemical studies show that the compounds are on the borderline between localized Robin‐Day class‐II and delocalized Robin‐Day class‐III species.

## Graphical abstract

**Donor–acceptor pyrenes**: Three new pyrene derivatives with strong donors at the 2,7‐positions and acceptors at the K‐region were synthesized. This leads to unusual properties such as emission in the red‐to‐NIR region (700 nm) and long intrinsic lifetimes of >100 ns, and the monocations absorb up to 3000 nm.

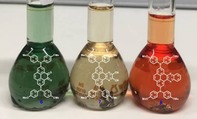

## Introduction

The polycyclic aromatic hydrocarbon (PAH) pyrene is among the most widely studied chromophores and possesses some unique properties such as intense blue emission and an exceptionally long‐lived excited singlet state together with excimer and exciplex formation.^1^ In addition to its photophysical properties, it has high chemical stability and charge‐carrier mobility. Therefore, pyrene derivatives have been used in a broad range of applications in diverse scientific fields such as organic light‐emitting diodes (OLEDs), organic field‐effect transistors (OFETs) and organic photovoltaic cells (OPVs).^1^, ^2^ Furthermore, they have been used for sensing of temperature,^3^ pressure^4^ or pH,^5^ or to detect guest molecules such as O_2_ or NH_3_,^6^ organic molecules,^7^ or metals^1^, ^8^ and to construct covalent organic frameworks.^9^ Its monomer and excimer fluorescence have also been used to study the properties such as dynamics or interactions of macromolecules^10^ and lipids.^11^ Its remarkably long fluorescence lifetime of 354 ns, compared to other PAHs, makes pyrene exceptionally well‐suited for further applications such as the determination of cellular oxygen concentrations or reactive oxygen species (ROS) in biological systems.^12^ Furthermore, 2‐phenylethynylpyrenes have also been used as fluorescent labels for DNA.^13^

To adjust the properties for a specific application, electron donors and/or acceptors are often introduced onto a PAH core as they strongly modulate the frontier orbitals. Substituting PAHs with donors and acceptors gives derivatives with properties such as a permanent dipole moment, charge transfer (CT) excited states, strong solvatochromism, environmentally influenced photophysics, the possibility for energy or electron transfer, and narrowed energy gaps.^14^, ^15^ Furthermore, such chromophores can absorb and emit in the near‐infrared (NIR) region, which is in demand for applications such as bioimaging and cell recognition, as NIR light penetrates deeper into biological tissues, is less damaging than visible or UV light, and gives minimum interference from background autofluorescence by biomolecules.^16^ In general, materials with high HOMO energies, such as the compound *N*,*N′*‐diphenyl‐*N*,*N′*‐bis(3‐methylphenyl)‐(1,1′‐biphenyl)‐4,4′‐diamine (TPD), are especially useful for hole transport.^17^ Common π‐donors that have been used in dyes to boost HOMO energies include amines, with a lone pair on the nitrogen, such as diarylamino, diethylamino, dimethylamino or carbazolyl moieties.^18^ Diarylamines are among the strongest π‐electron donors and have been employed in diverse applications,^19^ due to their outstanding physical, photochemical and electrochemical properties, and they are easy to synthesize and handle.^20^ A methoxy group at the position *para* to the nitrogen not only increases the electron donating strength of diarylamines, but enables reversible oxidations.^20^, ^21^ Nevertheless, a significant drawback can be energy loss due to the rotational motion of the phenyl rings which can result in a reduction in luminescence efficiency.^22^ In addition, the possibility of rotation around the N−C(π) bond can lead to twisted intramolecular charge transfer (TICT) excited states.^15^ The compound 1,1,7,7‐tetramethyl‐julolidine is known to be an even stronger π‐donor than diarylamines. The julolidine moiety has been thoroughly studied since its discovery in 1892 by Pinkus,^23^ and is used in a wide range of dyes.^15^, ^24^ Its nitrogen lone pair is conformationally restricted to remain parallel to the aromatic system,^25^ in our case, the pyrene moiety, in both the ground and excited states, and our previous studies revealed a significantly enhanced electron donating effect on the pyrene core compared to diarylamines.^15^

**Figure 1:**
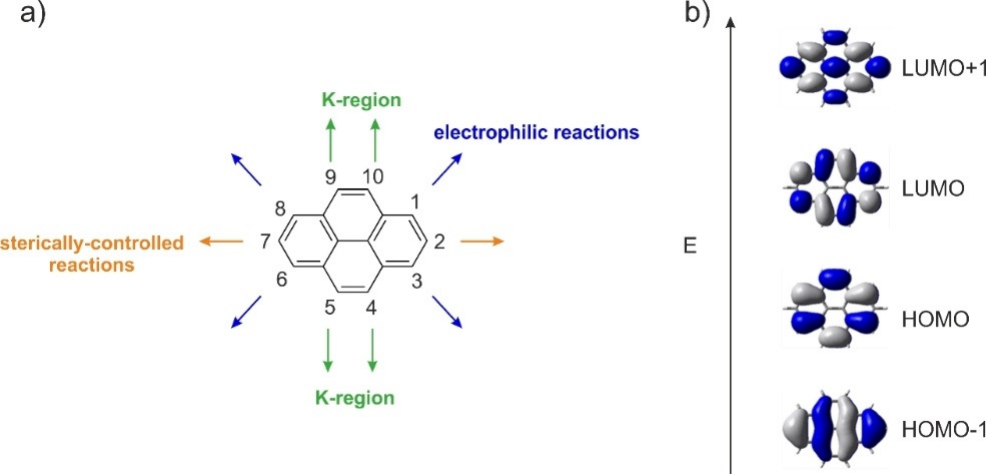
a) Atom numbering system in pyrene with the three sets of chemically inequivalent sites; b) the four frontier orbitals of pyrene.

**Scheme 1:**
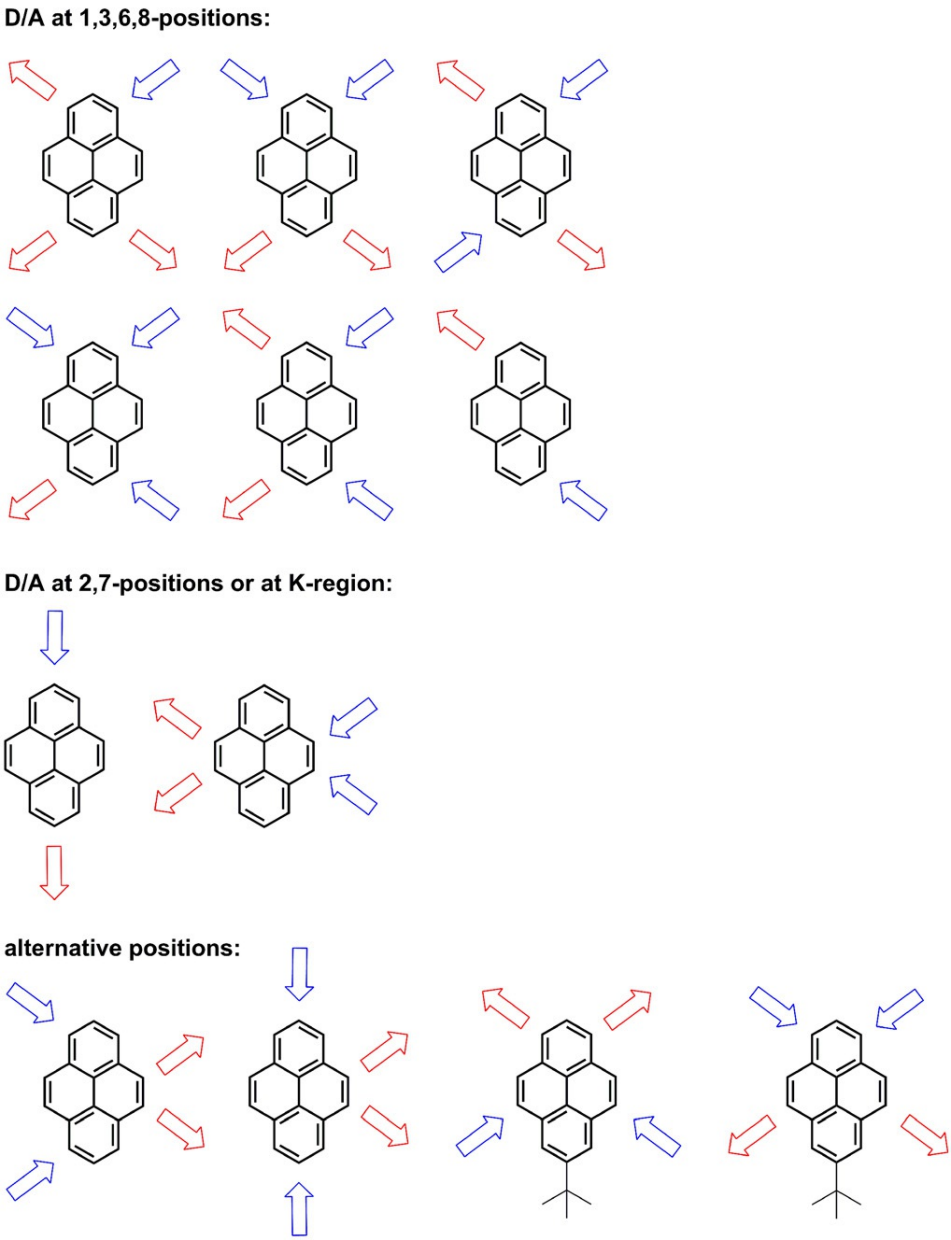
Schematic representation of reported D/A‐pyrene patterns.

Pyrene exhibits ten peripheral reactive positions, which can be classified into three sets of chemically inequivalent sites (Figure 1 a). Thus, the position of substitution is very important and, typically, the 1‐, 3‐, 6‐ and 8‐positions are functionalized by electrophilic substitution reactions as the HOMO has its largest coefficients at these positions (Figure 1 b).^1^, ^26^ Therefore, π‐orbitals of substituents at the 1‐, 3‐, 6‐, and 8‐positions mix very efficiently with pyrene's HOMO/LUMO orbitals. However, unsymmetrical substitution at these positions is rather challenging due to the numerous possible isomers (Scheme 1). Niko, Konishi and co‐workers synthesized D–π–A pyrene systems with donors and acceptors at the 1‐,3‐,6‐ and 8‐positions, which displayed strong solvatochromism with emissions into the red region (557–648 nm, in MeOH) and high quantum yields (*φ*>0.75).^27^

**Scheme 2:**
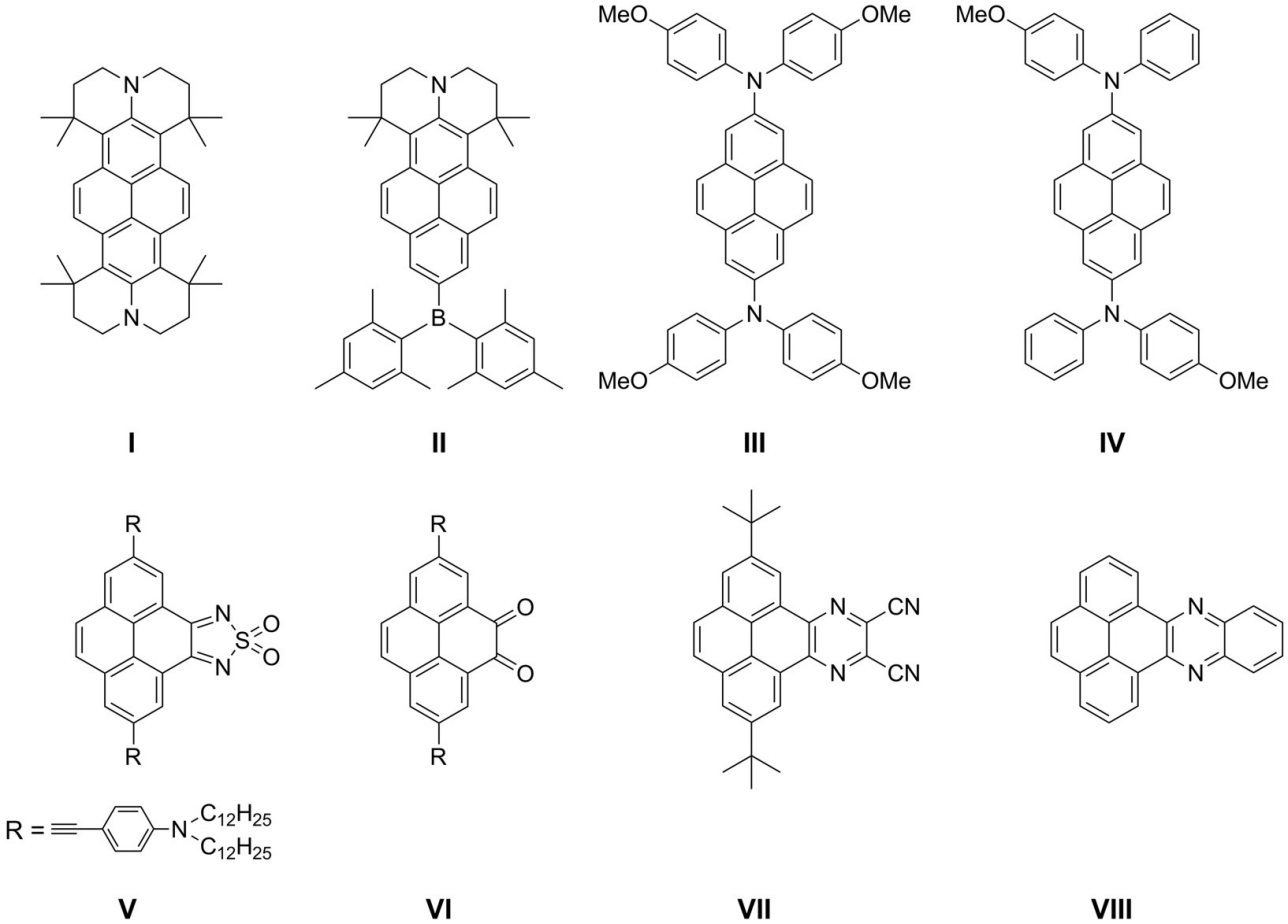
Schematic overview of some important pyrene derivatives that possess either the same acceptor or similar donor moieties as our derivatives **6**, **7**, or **8**.

Substituents at the 2,7‐positions do not interact with the HOMO/LUMO orbitals of pyrene as they lie on the nodal plane (Figure 1 b). However, they can interact strongly with the HOMO−1 and LUMO+1 of pyrene that have nonzero contributions at these positions. We previously reported that the photophysical properties of pyrenes with substituents at the 2‐position significantly differ from those with substituents at the 1‐position.^28^ Moreover, previous studies demonstrated that strong π‐donors/acceptors are able to switch the energetic ordering of its HOMO/HOMO−1 and LUMO/LUMO+1, respectively, which results in greatly influenced photophysical and redox properties of these derivatives.^15^, ^29^, ^30^, ^31^, ^32^ Unsymmetrical substitution at these positions is straightforward via iridium‐catalyzed C−H borylation,^33^, ^34^, ^35^ and the introduction of julolidine‐like donors at the 2,7‐positions (**I,** Scheme 2) even resulted in an unusual green luminescence.^15^, ^29^, ^30^, ^31^, ^32^, ^36^

The K‐region (positions 4,5,9,10, Figure 1) has large HOMO/LUMO as well as HOMO−1/LUMO+1 contributions. Ottonelli and co‐workers showed^37^ that the contribution to the HOMO/LUMO increases in the order: 2,7<4,5,9,10<1,3,6,8, while contributions to the HOMO−1/LUMO+1 increase in the order 4,5,9,10<1,3,6,8<2,7. The K‐region possesses alkene‐like rather than aromatic character. Hence, reagents such as osmium tetroxide, which are known to react with alkene double bonds, selectively react with pyrene at the 4,5,9,10‐positions. Müllen and co‐workers reported a protocol for the asymmetric substitution at these sites and presented a few examples with emission into the orange region of the spectrum (613 nm in THF) (Scheme 1).^14^, ^38^

There are also reports on pyrenes that have donor and acceptor moieties on alternative positions, besides the typical three categories (vide supra). Hu and co‐workers developed unsymmetric pyrenes with donors at the 1,3‐positions and acceptors at the 5,9‐positions that emit in the blue‐green region (Scheme 1).^39^ Sutherland and co‐workers combined the 2,7‐ and 1,8‐positions with the K‐region (**V** and **VI**, Scheme 2), respectively, and obtained an S_1_←S_0_ absorbance up to 900 nm, as it was their objective to obtain strongly redshifted absorptions with high molar absorptivities. However, their derivatives are not emissive and thus studies are missing on the influence on the excited state properties and how additional K‐region substituents influence 2,7‐substituted pyrenes.^40^, ^41^

In our previous reports we showed that the influence of a second amine donor at the 2,7‐positions on the occupied orbitals is larger than that of a cyano or even Bmes_2_ acceptor on the unoccupied orbitals and thus emission from the D–π–D derivative (**I**, Scheme 2) is further redshifted than the D–π–A compound (**II**, Scheme 2). Therefore, our initial objective was to utilize two julolidine‐like donors at the 2,7‐positions and add additional acceptors to the pyrene core to obtain a new class of D–π–A pyrene derivative with unparalleled photophysical properties such as red to NIR emission and interesting redox behavior. Moreover, we were interested to see how much more the S_1_ state can be influenced by the addition of acceptors to the K‐region.

For the acceptor unit, we chose *n*‐azaacenes fused to the K‐region of pyrene that were reported by Mateo‐Alonso and co‐workers and which possess strong π‐accepting properties (**VII** and **VIII**, Scheme 2).^43^ N‐azaacenes have attracted much attention for their outstanding electronic properties and application in organic electronic devices and as anion radicals.^42^ In particular, pyrene‐fused azaacenes are even more stable than their N‐azaacene analogues and emit strongly in the blue‐to‐green region of the electromagnetic spectrum.^43^, ^44^

## Results and Discussion

### Synthesis and structural characterization

**Scheme 3:**
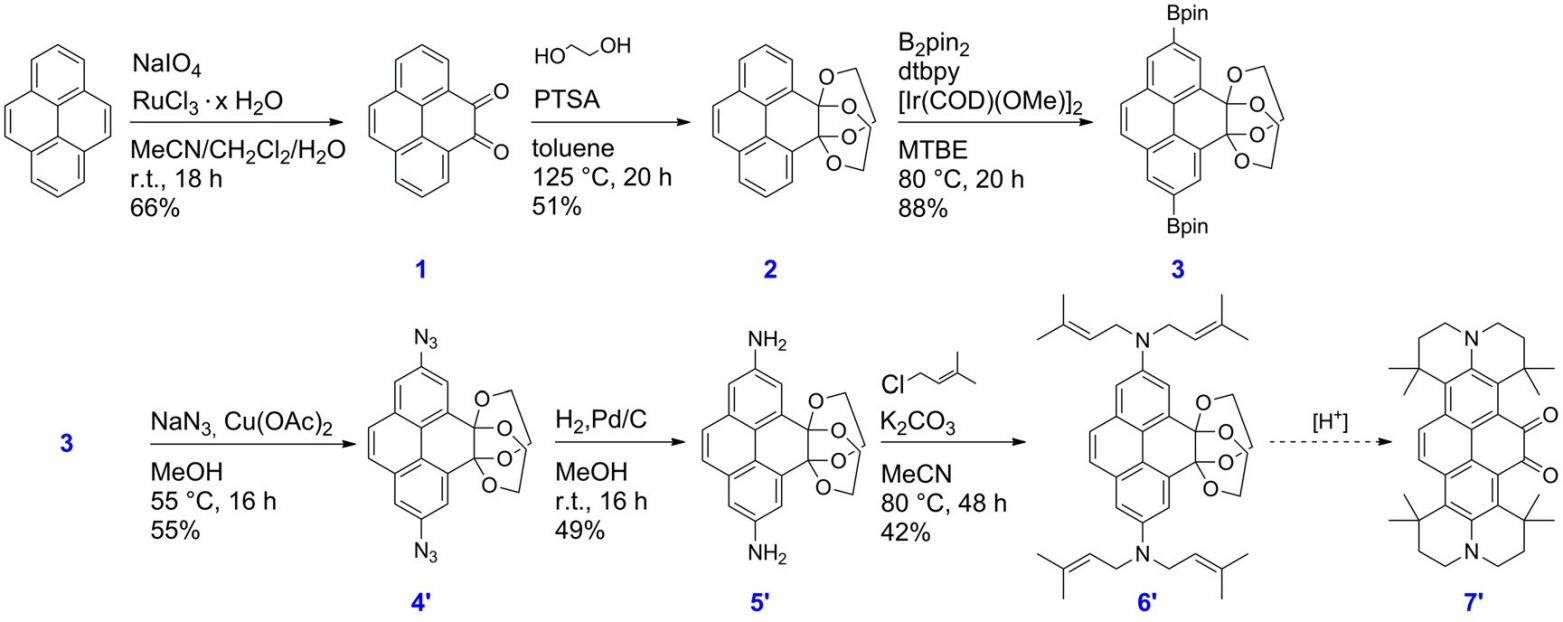
Synthesis of derivatives **1**–**3** and **4′‐7′**.

**Scheme 4:**
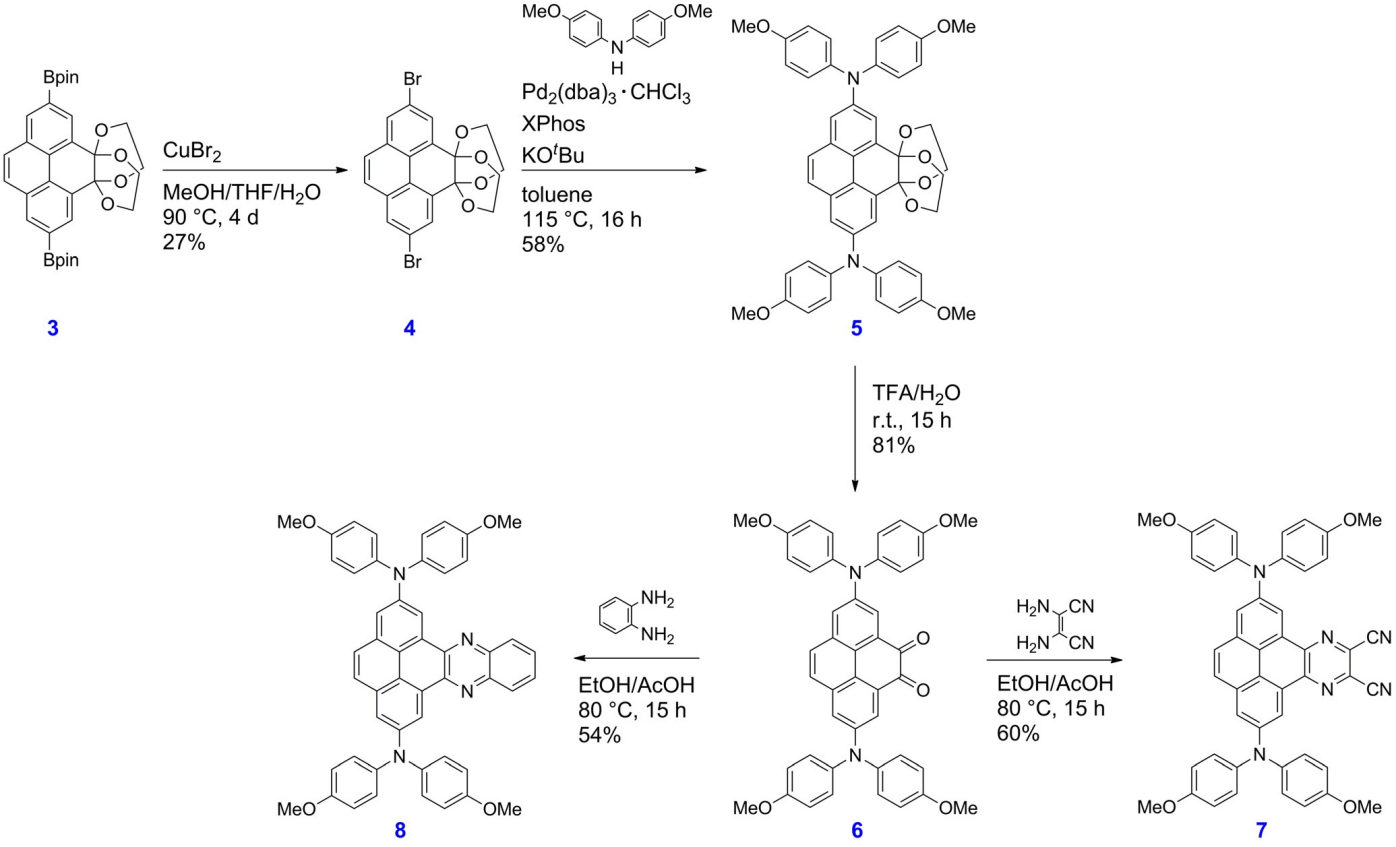
Synthesis of derivatives **3**–**8**.

The procedures used to synthesize the three key compounds are summarized in Schemes 3 and 4. At the beginning of this project, we aimed for derivative **7′** (Scheme 3) and its analogue with ketone moieties at all four K‐region positions (Scheme S1). The selective RuO_4_‐catalyzed oxidation of pyrene at its 4‐ and 5‐positions is the starting point for the synthesis route and gives the dione **1**. This one‐step reaction was reported by Harris and co‐workers in 2005 as an alternative to the toxic OsO_4_‐catalyzed oxidation of pyrene.^47^, ^48^ In this regard, RuO_4_ is generated in situ from RuCl_3_⋅3 H_2_O and NaIO_4_, and the oxidation takes place in a solvent mixture of MeCN, CH_2_Cl_2_ and H_2_O (1:1:1.25). In 1981, Sharpless and co‐workers reported that addition of MeCN to the RuO_4_‐catalyzed oxidation greatly improves the effectiveness of the reaction. However, this procedure suffers from several disadvantages. Thus, the reaction can only be performed on a small scale and, furthermore, there are various side products such as dialdehydes or acids, which Nowicka et al. identified in detail, that complicate the workup and reduce the yield.^49^ Bodwell and co‐workers recently introduced an improved RuO_4_‐catalyzed oxidation procedure to obtain **1**, which is scalable and eases the workup. This method includes the additive *N*‐methylimidazole, and the solvent MeCN is replaced by THF.^50^ In order to perform the Ir‐catalyzed C−H borylation,^33^, ^34^, ^35^ the ketone groups had to be protected as a diketal to prevent unwanted reactions with the Ir‐catalyst. The lack of reactivity of oxidized pyrene in other metal‐catalyzed reactions has been reported before.^51^, ^52^ Compound **2** was easily obtained by refluxing **1** in toluene with an excess of ethylene glycol for 20 h in the presence of *p*‐toluenesulfonic acid. The C−H borylation was performed according to our reported procedure^33^, ^34^, ^35^ and takes place at the 2,7‐positions selectively providing **3**.

The Bpin moieties were further converted to azide moieties using a procedure reported by Chang and co‐workers.^53^ They converted arylboronic acids to arylazides in MeOH by using 1.50 equivalents of NaN_3_ as the azide source and 5 mol % of Cu(OAc)_2_ as the catalyst. Subsequent reduction of the azido species **4′** with Pd/C and H_2_ gave the corresponding primary amine **5′**. The reaction was stirred at room temperature until monitoring by IR spectroscopy confirmed the complete disappearance of azide moieties (in the region 2000–2270 cm^−1^) and the appearance of primary amine bands at 3450, 3363 and 1620 cm^−1^ (Figure S26). The amine moieties were alkylated in MeCN with 1‐chloro‐3‐methylbutene in the presence of the base K_2_CO_3_, according to the approach used in our previously reported synthesis of **I**.^15^ The alkylation reaction was complete after 48 h and gave the desired product **6′** as a bright yellow solid (42 %). Unfortunately, the final step to obtain the desired derivative **7′** was unsuccessful. Our aim was to achieve ring closure and simultaneously deprotect the acetal groups in order to regenerate the two ketone moieties at the K‐region. However, using a diverse range of acids, the acetal protecting groups were removed first, which most likely generated a pyrene core that was too deactivated to permit the electrophilic ring closure to take place. Therefore, we decided to change our julolidine‐like donor moiety to a diarylamine. The new synthesis route is depicted in Scheme 4. Compound **3** was transformed into the corresponding dibromo species **4** by a bromodeboronation reaction.^54^ Thus, **3** and CuBr_2_ were suspended in a mixture of THF/MeOH/H_2_O (1:1:1) and the reaction mixture was stirred at 90 °C for 4 d. The Buchwald–Hartwig amination, which was performed to obtain **5** from **4** using Pd_2_(dba)_3_⋅CHCl_3_ as the catalyst precursor and XPhos as the ligand, was achieved in a yield of 58 %. The deprotection of **5** in a trifluoroacetic acid and water mixture (6:1) is straightforward, and compound **6** was obtained in 81 % yield. The cyclocondensation reactions between dione **6** and 2,3‐diaminomaleonitrile to give **7**, or benzene‐1,2‐diamine to give **8**, were performed in an ethanol/acetic acid mixture (1:1) at 80 °C for 15 h according to the procedure of Mateo‐Alonso and co‐workers.^43^, ^51^, ^55^

**Figure 2:**
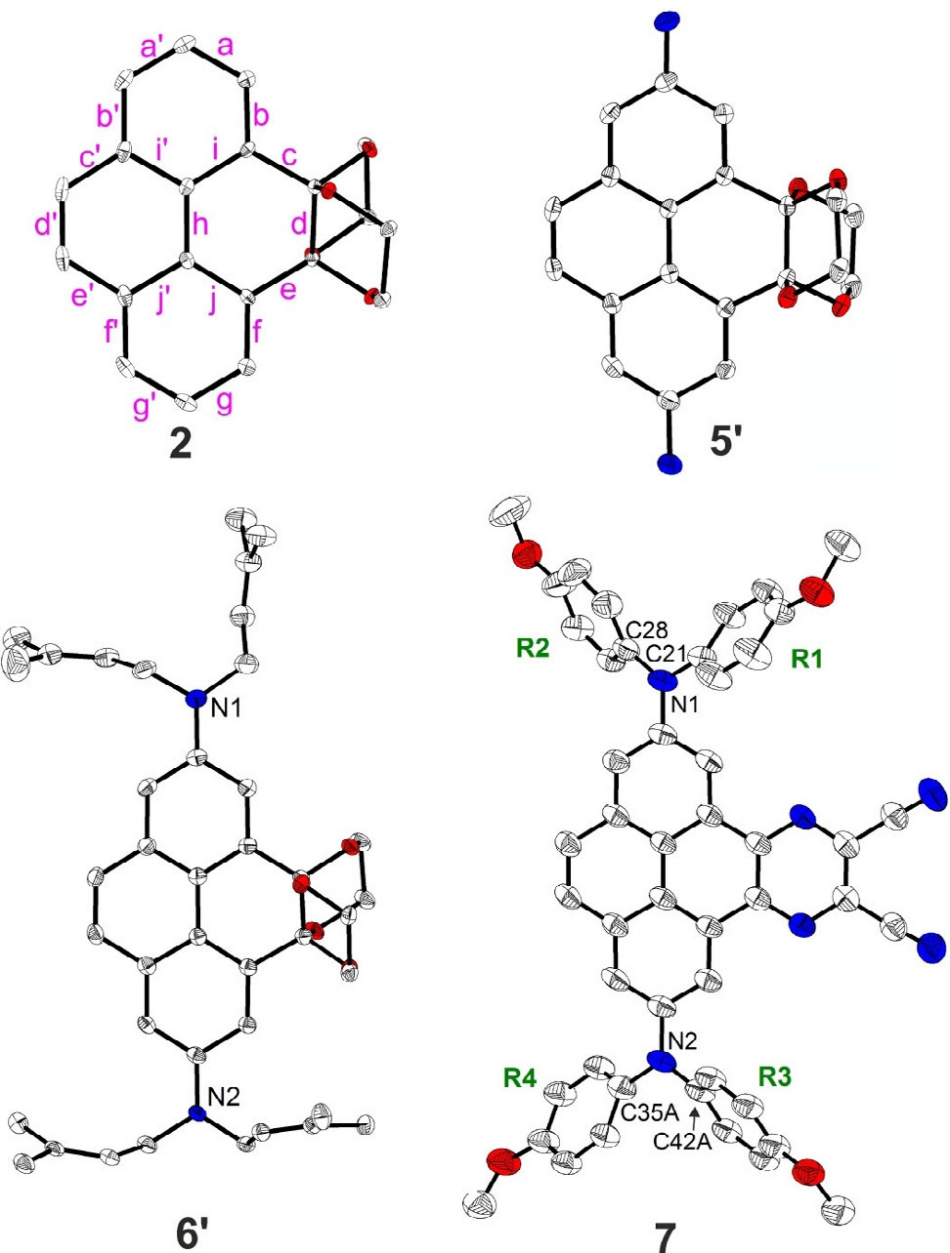
Solid‐state molecular structures of compounds **2**, **5′**, **6′**, and **7** as determined by single‐crystal X‐ray diffraction at 100 K. Hydrogen atoms, solvent molecules, and disordered parts of lower occupancies are omitted for clarity. Atomic displacement ellipsoids are shown at the 50 % probability level. Element color: carbon (white), nitrogen (blue), oxygen (red). Representative bond labeling is shown on compound **2**. Aryl rings are labeled R1 to R4 in **7**.

The solid‐state structures of compounds **2**, **5′**, **6′**, and **7** were determined via single‐crystal X‐ray diffraction (Figure 2). In compound **7**, the biphenyl unit of the pyrene moiety exhibits typical aromatic C−C bond lengths ranging from 1.391(4) to 1.418(3) Å (bonds **a**, **b**, **f**, **g**, **i**, and **j** and their symmetrical equivalents, Figure 2 and Table S2), as is also observed in pyrene. The **c′** and **e′** bonds on the unsubstituted site of the pyrene moiety are slightly longer (1.444(3) and 1.431(4) Å), and the **d′** bond (1.355(3) Å) is typical of a C=C double bond. This means that it can be viewed as a biphenyl unit constrained to be planar by a ‐CH=CH‐ group. This has also recently been observed for many other 2‐, and 2,7‐substituted pyrenes by Marder and co‐workers.^15^, ^29^, ^30^ Similar bond lengths have also been reported for both sides of the pyrene moiety of 2,7‐bis(dianisylamino)pyrene,^32^ the azaacene‐free analogue of our compound **7**. However, in **7** the **c**, **d**, and **e** bonds on the 4,5‐azaacene‐substituted side of the pyrene moiety are all longer than the equivalent bonds on the unsubstituted side, the **d** bond (1.426(3) Å) being shorter than the **c** and **e** bonds (1.451(3) and 1.453(3) Å) (Table S2). Hence, the incorporation of the azaacene moiety at the 4,5‐positions has a bond‐lengthening effect, and the **d** bond can no longer be compared with a C=C double bond but rather has aromatic character. Interestingly, the azaacene‐moiety also effects the central bond **h** which links the two phenyl rings, and is slightly longer (1.435(3) Å) than in the azaacene‐free analogue compound (1.416(2) Å).^32^

A similar, even more pronounced effect is observed in the unsymmetric compounds with acetals substituted at the 4,5‐positions of pyrene, that is, compounds **2**, **5′**, and **6′**. While the unsubstituted side of the pyrene core still shows bond distances similar to those of pyrene or compound **7**, the 4,5‐substituted side shows long bond distances for the **c**, **d**, and **e** bonds (1.515(2)–1.555(3) Å), which are typical of C−C single bonds (Table S2). The central **h** bond is also further elongated (1.440(3)–1.447(3) Å) with respect to **7**.

As is usually observed for aromatic amines, in compound **7** the N−C(pyrene) bond lengths are significantly shorter (1.406(3) and 1.415(3) Å) than the other N−C bonds of the amine moieties (1.422(3)–1.435(2) Å). Similar distances were also reported for the azaacene‐free pyrene with amines substituted at the 2‐ and 7‐ positions.^32^ The nitrogen atoms have a nearly ideal trigonal planar configuration with the sum of the C‐N‐C angles being between 359.0(6) and 360.0(3)°. The interplanar angles between the NC_3_ and pyrene planes (37.45(10) for N1 and 32.3(2)–33.2(4)° for the disordered groups bonded to N2) are in a similar range as those between NC_3_ and the terminal phenyl rings (35.2(4)–47.5(2)°) (Table S2). Again, this is in agreement with the NC_3_–pyrene angle (31°) which was reported for the analogous azaacene‐free 2,7‐substituted pyrene compound.^32^ The methoxyphenyl groups R3 and R4 of **7** are strongly disordered and show a higher degree of rotational freedom than the groups R1 and R2. Indeed, the interplanar angles between the NC_3_ planes and the terminal rings vary between 38.2(4) and 47.5(2)° for the disordered parts of R3 and between 35.2(4) and 39.0(6)° for those of R4. In addition, important intermolecular interactions involving the methoxyphenyl groups are only present for the R1 and R2 groups but are not observed for the R3 and R4 groups (Table S4).

**Figure 3:**
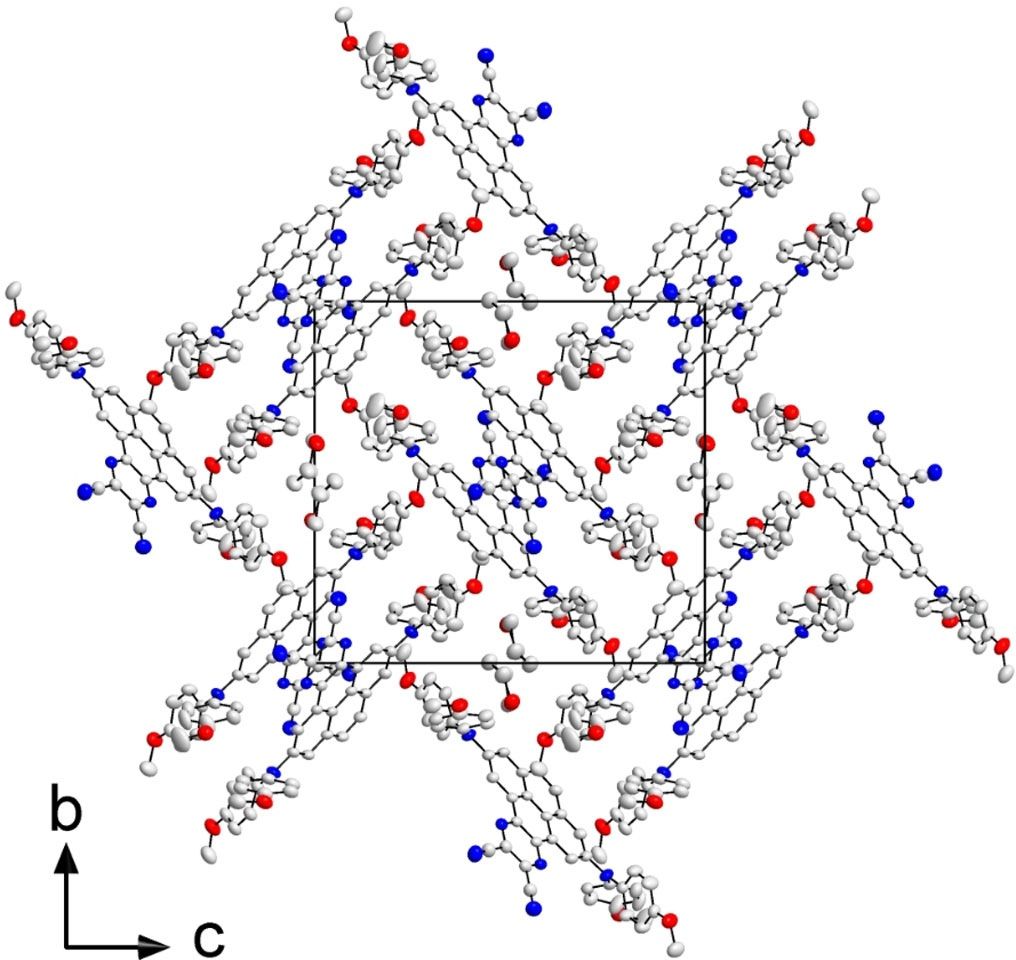
Crystal structure of compound **7** projected along the *a* axis, at 100 K. Hydrogen atoms and disordered parts of lower occupancies are omitted for clarity. Compound **7** crystallizes in a sandwich‐herringbone packing wherein the herringbone is made up of sandwich‐like diads with both edge‐to‐face and offset face‐to‐face interactions of π‐stacked molecules that have an interplanar distance of 3.41–3.47 Å. Atomic displacement ellipsoids are shown at the 50 % probability level. Element color: carbon (white), nitrogen (blue), oxygen (red).

In the crystal structure of **7**, the molecules form π‐stacked dimers related by inversion symmetry. Hence, a π‐stacking interaction with an interplanar separation of around 3.41–3.47 Å is present between the pyrene core and the azaacene group (Table S4). Dimers are arranged edge‐to‐face in a sandwich‐herringbone packing, which is typically observed for pyrene itself and its derivatives (Figure 3).^56^ Parallel and inverted molecules that are offset along the *a* axis exhibit C⋅⋅⋅C intermolecular interactions between their CN end groups. The molecular packing is further determined by the large steric demand of the amine moieties and the presence of tetrahydrofuran solvent molecules in the crystal lattice. Intermolecular C−H⋅⋅⋅N and H⋅⋅⋅H interactions are present between the R1 and R2 methoxyphenyl groups and the pyrene‐4,5‐azaacene core, while C−H⋅⋅⋅O interactions exist with the tetrahydrofuran molecule (Table S4). A Hirshfeld surface analysis was performed in order to quantify the nature and type of intermolecular interactions in **7**.^57^ The disorder of the methoxyphenyl groups was not taken into account, but only the parts of highest occupancies were considered in the analysis. Fingerprint analysis and its breakdown to the individual relative contributions,^58^ shows a major contribution from H⋅⋅⋅H interactions (42 %), followed by a significant amount from C⋅⋅⋅H (22 %), N⋅⋅⋅H (15 %), and O⋅⋅⋅H (10 %) interactions (Figures S44 and S45); 5 % C⋅⋅⋅C interactions and around 3.6 % N⋅⋅⋅C interactions are observed as well.

### Photophysical and redox properties

**Figure 4:**
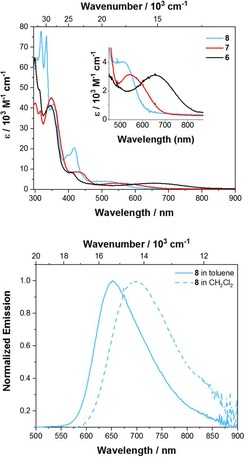
Absorption (top) of **6**, **7** and **8** recorded in toluene and emission (bottom) spectrum of **8** recorded in toluene and CH_2_Cl_2_.

The absorption spectra of derivatives **6**, **7** and **8** are depicted in Figure 4 and are generally similar to that of pyrene, in that the S_1_←S_0_ absorption is comparably weak with extinction coefficients of ***ϵ***=2 700–4 000 m ^−1^ cm^−1^. However, for 2,7‐substituted pyrenes, these are the largest extinction coefficients reported so far. Thus, the acceptor moieties at the K‐region increase how allowed this transition is. Furthermore, the S_1_←S_0_ absorption has a strong bathochromic shift in the order **8**<**7**<**6**, and remarkably broad, covering a range of 6 000 cm^−1^, with no vibrational progression, providing an indication of strong charge transfer (CT) character. In particular, dione **6** has a very pronounced bathochromic shift (*λ*_max_ (abs)=658 nm), which is significantly stronger compared to the analogous dione **VI** reported by Sutherland and co‐workers (Scheme 2).^40^ However, the donor moieties in their derivative are further separated from the pyrene core by alkyne and phenyl spacers.^59^ Hence, the donating ability through the 2,7‐positions is reduced and, as a result, a weaker CT character is obtained. Thus, derivative **VI** has a smaller bathochromic shift with *λ*_max_ (abs)=575 nm compared to derivative **6**. It is interesting to observe that the substituents at the 2,7‐positions in these derivatives have such a significant effect on the S_1_←S_0_ absorption, which is unusual. The CT character of these derivatives is even more evident when comparing them to their analogues without the donor moieties at the 2,7‐positions. Mateo‐Alonso and co‐workers reported^45^ that *λ*_max_ (abs)=455 nm for **VII**, the analogue of our derivative **7**, which demonstrates that the resulting CT character introduced via the additional donors causes a bathochromic shift of 3500 cm^−1^ of the S_1_←S_0_ absorption in our systems. Compound **VIII**, the analogue of our derivative **8,** has *λ*_max_ (abs)=435 nm according to Sahoo et al. Hence, the CT character introduced through our additional donors at the 2,7‐positions shifts the S_1_←S_0_ absorption by 3700 cm^−1^.

Compounds **6** and **7** are not emissive; however, **8** emits in the red to NIR region of the electromagnetic spectrum with *λ*_max_ (em)=652 nm in toluene (Table 1), which is intriguing for pyrenes as they usually emit in the blue region and, to the best of our knowledge, such a red‐shifted emission has not been reported before for monomeric pyrenes. Even pyrene‐fused azaacenes reported previously do not have such a red‐shifted emission.^55^ Furthermore, compound **8** exhibits significant solvatochromism, as the emission shifts bathochromically with increasing solvent polarity from toluene to CH_2_Cl_2_ by 1051 cm^−1^ (48 nm), confirming the CT nature of the lowest energy excited state and a significant change in dipole moment between ground state and excited state. The large CT nature of the excited state becomes more evident when comparing the emission of **8** with previously reported analogues that only possess a donor or acceptor moiety. Derivative **VIII** emits in the blue region with *λ*_max_ (em)=471 nm and compound **IV** (unfortunately, there is no report of an emission of compound **III**) emits in the green region with *λ*_max_ (em)=520 nm (both in CH_2_Cl_2_) while we observe a *λ*_max_ (em)=700 nm for **8** in CH_2_Cl_2_.^32^, ^46^ Nevertheless, the non‐radiative decay rates are significantly increased in CH_2_Cl_2_ (*k*_nr_=11×10^7^ s^−1^), which is fully in line with the energy gap law as the reorganization energy is enhanced in polar solvents.^61^ Therefore, the quantum yield is strongly decreased in the polar solvent. The apparent Stokes shifts are very large for pyrenes with values ranging from 3900 to 4900 cm^−1^ and are much larger compared to pyrenes that have D/A groups at the 2,7‐ or 1,3,6,8‐positions only.^15^, ^27^, ^28^, ^30^ However, the pyrene derivatives reported by Müllen and co‐workers that have D/A moieties at the K‐region exhibit even larger Stokes shifts ranging from 4500 to 5400 cm^−1^.^14^ Interestingly, the radiative decay rates are rather slow (*k*_r_=0.3–1.0×10^7^ s^−1^), which is a result of the strong CT character. However, such slow radiative rate constants are also typical of forbidden transitions (Strickler–Berg relation) and, thus, the lifetimes remain quite long (*τ_0_*=104–293 ns) in this derivative, which is a typical property of 2,7‐substituted pyrenes.^28^ There are not many reports on lifetimes of K‐region substituted pyrenes, but they are typically in the range of (*τ*_0_=13–16 ns), which we recently reported.^62^ Müllen and co‐workers do not give lifetimes for their K‐region D/A derivatives; however, the S_1_←S_0_ absorptions are significantly more allowed with *ϵ*>7000 m ^−1^ cm^−1^ and, therefore, shorter lifetimes than for 2,7‐substituted pyrenes can be assumed.^14^, ^63^ Compared to compound **8**, derivative **7** has a more pronounced CT nature, hence its energy gap is even smaller and, thus, it is possible that the non‐radiative decay rates are largely increased, so that fluorescence becomes too weak to detect.

**Table 1:** Selected photophysical data of the derivatives **6**, **7**, and **8** recorded under argon at room temperature.

Cpd	Medium	*λ*_abs_ [nm] (*ϵ* [10^3^ m ^−1^ cm^−1^])	*λ*_em_ [nm]^[a]^	Apparent Stokes^60^ shift [cm^−1^]	*τ* [ns]^[b]^	φ	*τ*_0_ [ns]	*k*_r_ [10^7^ s^−1^]	*k*_nr_ [10^7^ s^−1^]
**6**	toluene	658 (2.7), 408 (7.0), 344 (41), 299 (31)	–	–	–	–	–	–	–
**7**	toluene	542 (3.1), 427 (8.5), 348 (48), 300 (42)	–	–	–	–	–	–	–
**8**	toluene	519 (4.0), 417 (21), 399 (17), 354 (42), 333 (76), 317 (78)	652	3930	32.3	0.31	104	1.0	2.1
**8**	CH_2_Cl_2_	521, 416, 397, 352, 332, 316	700	4908	8.8	0.03	293	0.3	11.0
**8**	solid	‐	679	–		<0.01

[a] Excited at the respective *λ*_max_ (abs) of S_1_←S_0_.

### Electrochemistry

**Figure 5:**
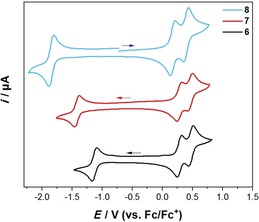
Cyclic voltammograms of **6**, **7**, and **8** in CH_2_Cl_2_/0.1 m [*n*Bu_4_N][PF_6_] with a scan rate of 250 mV s^−1^.

In order to determine the impact of the acceptor groups on the K‐region in combination with the donors at the 2,7‐positions, cyclic voltammetry was performed. The cyclic voltammograms are shown in Figure 5 and the respective reduction and oxidation potentials are given in Table 2. All derivatives exhibit one reversible reduction, whereas compound **6** has the lowest reduction potential with *E*_1/2_=−1.13 V and **8** the highest with *E*_1/2_=−1.84 V vs. Fc/Fc^+^. Hence, the accepting strength of our derivatives is quite strong and decreases in the order **6**>**7**>**8**. Furthermore, all three derivatives can be reversibly oxidized twice; derivative **8** is the easiest to be oxidized with *E*_1/2_=0.17 and 0.39 V, and derivatives **6** and **7** have the same first (*E*_1/2_=0.28) and second (0.45 V) oxidation potentials versus Fc/Fc^+^. Hence, our derivatives possess quite strong donors and the donating strength in **8** is only minimally influenced by the additional acceptor as compound **III** has oxidation potentials of *E*_1/2_=0.14 and 0.38 V vs. Fc/Fc^+^. A similar trend is observed in our derivative **6**, as the reduction potential is comparable to that of the analogous derivative **VI**, which was reported by Sutherland^40^ (Table 3). Consequently, the different donating abilities at the 2,7‐positions do not influence the accepting effect of acceptors at the K‐region. This observation is also in line with the reduction potential of our derivative **7**, which is very similar to that of compound **VII** which has no donors at the 2,7‐positions (Table 3).

**Table 2:** Cyclic voltammetry results for **6**, **7**, and **8** in CH_2_Cl_2_/0.1 m [*n*Bu_4_N][PF_6_] relative to the Fc/Fc^+^ couple.

Cpd	*E*_1/2_ [V] [red]^1^	*E*_1/2_ [V] [ox]^1^	*E*_1/2_ [V] [ox]^2^
**6**	−1.13	0.28	0.47
**7**	−1.42	0.28	0.45
**8**	−1.84	0.17	0.39

**Table 3:** Selected optical and electrochemical properties of the pyrene derivatives **I** to **VIII**.

	*λ*_abs_ [nm]	*λ*_em_ [nm]	*E*_1/2_ [V] [red]^[a]^	*E*_1/2_ [V] [ox]^1[a]^	*E*_1/2_ [V] [ox]^2[a]^
I^15^	475 (hexane)	511 (hexane)	–	−0.18 (CH_2_Cl_2_)	+0.26 (CH_2_Cl_2_)
	481 (THF)	525 (THF)			
II^15^	450 (hexane)	464 (hexane)	−2.51 (THF)	+0.29 (THF)	
	462 (THF)	497 (THF)			
III^32^	–	–	–	+0.14 (CH_2_Cl_2_)	+0.38 (CH_2_Cl_2_)
IV^28^	453 (toluene)	482 (toluene)	–	–	–
V^41^	630 (CHCl_3_)	–	−0.67 (CHCl_3_)	+0.39 (CHCl_3_)	–
VI^40^	575 (CHCl_3_)	–	−1.04 (CHCl_3_)	+0.33 (CHCl_3_)	–
VII^45^	455 (CH_2_Cl_2_)	538 (CH_2_Cl_2_)	−1.43 (THF)	–	–
VIII^46^	435 (CH_2_Cl_2_)	471 (CH_2_Cl_2_)	–	–	–

[a] Redox potentials were measured with the addition of 0.1 m [*n*Bu_4_N][PF_6_] and are referenced vs. the Fc/Fc^+^ couple.

### Spectroelectrochemistry

**Figure 6:**
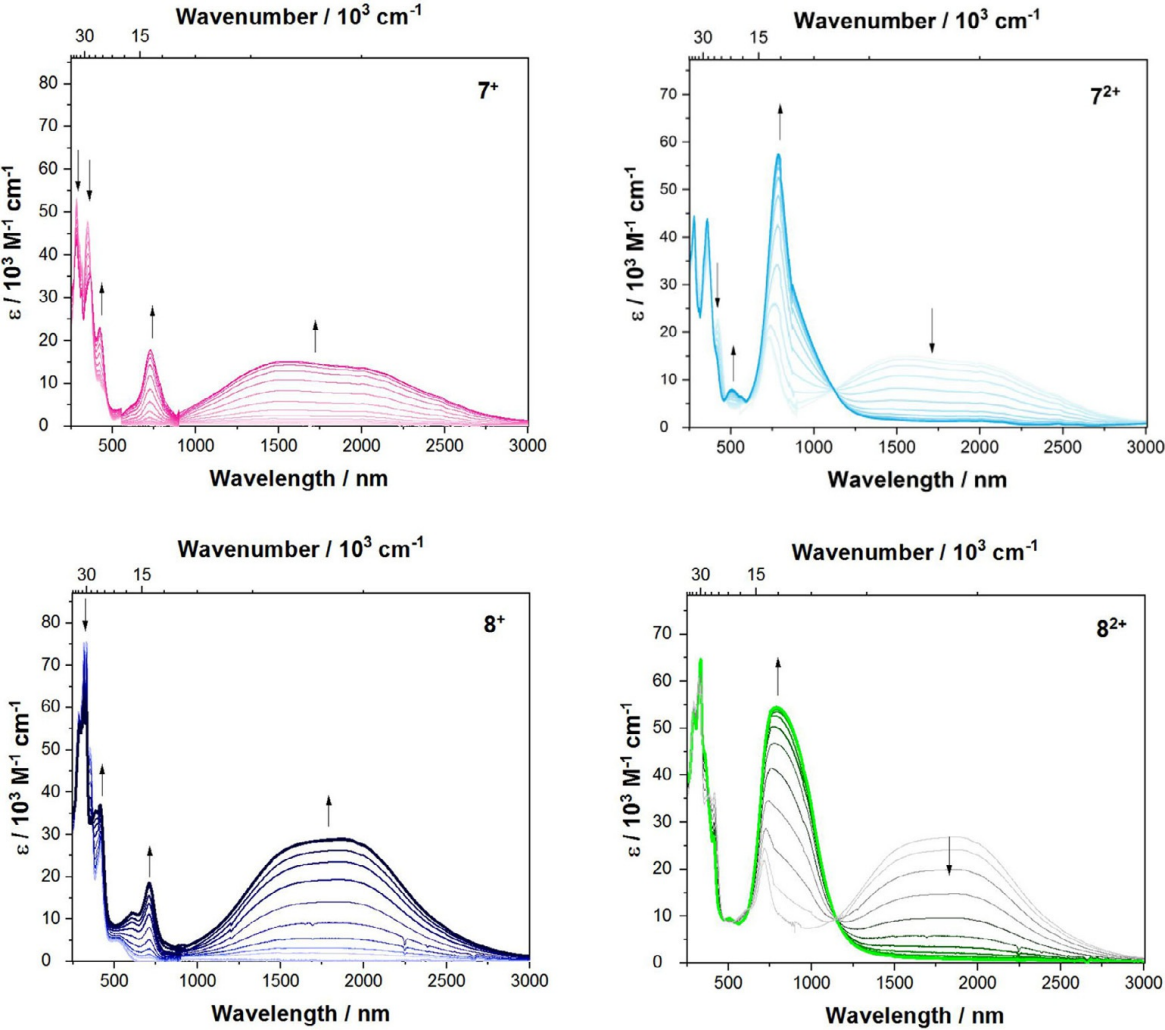
Spectroelectrochemical measurements of the stepwise oxidation process of **7** and **8** in CH_2_Cl_2_/0.1 m [*n*Bu_4_N][PF_6_]. Top left: absorption spectrum of **7^+^** (magenta); top right: of **7^2+^** (cyan); bottom left: of **8^+^** (dark blue) and bottom right: of **8^2+^** (green).

To gain further information about the electronic structure of the corresponding pyrene **7^+^**, **7^2+^** and **8^+^**, **8^2+^** derivatives, we performed UV/Vis/NIR spectroelectrochemical measurements in CH_2_Cl_2_/0.1 m [*n*Bu_4_N][PF_6_] (Figure 6).

**Figure 7:**
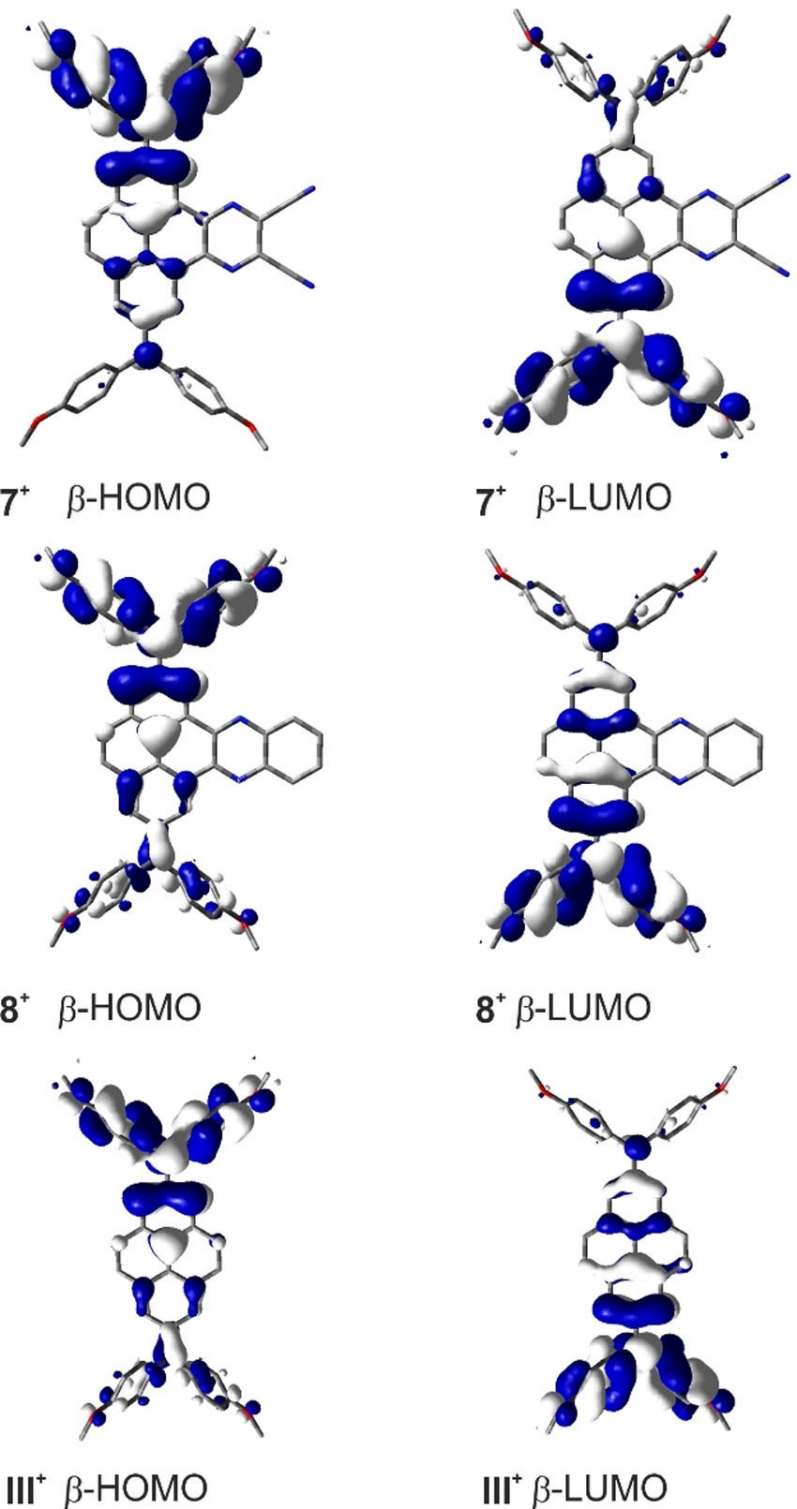
The UDFT computed MOs of **7^+^**, **8^+^**, and **III^+^** that are responsible for the lowest energy excitation.

Upon oxidation to the respective monocations **7^+^** and **8^+^**, a band rises in the NIR that is very broad, covering a range of around 7000 cm^−1^. The observed plateau may be caused by two overlapping peaks of a vibronic progression. Thus, the peak maximum depends on the relative intensities of the first and second overtones. For **7^+^**, the lowest energy band has a maximum at ${\tilde{v}}_{max}^{IVCT}$ =6400 cm^−1^ (1 563 nm, *ϵ*=15160 m ^−1^ cm^−1^), but for **8^+^**, the maximum is ${\tilde{v}}_{max}^{IVCT}$ =5350 cm^−1^ (1 869 nm, *ϵ*=28830 m ^−1^ cm^−1^) with some asymmetry in its shape (Figure S1). The absorption spectra of the monocations **7^+^** and **8^+^** are very similar to that of compound **III^+^**, which was reported by Ito and co‐workers, for which the lowest energy band has a maximum at 5 260 cm^−1^ (1900 nm) and is also slightly asymmetric.^32^ In general, delocalized (Robin‐Day class‐III) derivatives possess an asymmetric and narrow lowest energy band, while in localized (Robin‐Day class‐II) derivatives the lowest energy band results from an intervalence charge transfer (IV‐CT) with a well‐defined symmetric Gaussian shape.^64^ For that reason, Ito and co‐workers concluded that compound **III^+^** is a fully delocalized Robin‐Day class‐III derivative and assumed an electronic coupling *V* of $\frac{{\tilde{v}}_{max}^{IVCT}}{2}$ =2608 cm^−1^.^32^ However, our TD‐DFT computations on the monocations **7^+^** and **8^+^**, with a specially adjusted functional (BLYP with 35 % exact HF exchange^65^), show that the lowest energy excitation is a result of the promotion of an electron from the β‐HOMO to the β‐LUMO orbital and these orbitals show the expected phase behavior for localized Robin‐Day class‐II compounds (Figure 7). Further analysis of this lowest energy band enables the calculation of the electronic coupling *V* between the localized mixed valence states according to the Mulliken–Hush theory^66^ (Equation 1) where *μ*_ab_ is the transition moment (evaluated by integration of the IV‐CT band), and the diabatic dipole moment difference Δ*μ*_12_ was evaluated using the DFT calculated adiabatic dipole moment difference Δ*μ*_ab_.^67^ According to these calculations, both derivatives possess a large dipole moment change between their ground and excited states of Δ*μ*_ab_=42 D (**7^+^**) and 43 D (**8^+^**), which is typical for Robin‐Day class‐II compounds.

$$V=\frac{\mu_{ab}{\tilde{v}}_{max}^{IVCT}}{\Delta \mu_{12}}\;\;\mathrm{with}\;\;\Delta \mu_{12}=\sqrt{{\Delta \mu_{ab}}^{2}+4\mu_{ab}^{2}}\mathrm{ZS}>\left(1\right)$$

Hence, the electronic coupling *V* between the localized mixed valence states yields *V=*1279 cm^−1^ for **7^+^** and 1278 cm^−1^ for **8^+^**. Thus, the electronic coupling *V* of both derivatives is smaller than $\frac{{\tilde{v}}_{max}^{IVCT}}{2}$ , which shows that these compounds are class‐II compounds, but lie close to the borderline between localized class‐II and delocalized class‐III compounds, as *V* is quite large. This result is also in line with the observed broad and asymmetric band shape. Upon further oxidation to the respective dications, the IV‐CT band decreases and the band at 790–792 nm (12 660–12 630 cm^−1^) rises significantly (**7^2+^**: *ϵ_max_*=57 000 m ^−1^ cm^−1^, **8^2+^**: *ϵ_max_*=54 000 m ^−1^ cm^−1^). Furthermore, both spectra show bands rising at 727 nm (13 800 cm^−1^) and 415–421 nm (24 000 cm^−1^) while the bands at 320–350 nm (29 000–31 000 cm^−1^) decrease, which is also very similar to the observations made for the oxidation to the monocation of compound **III**.^32^

Consequently, the acceptor moieties at the K‐region of the pyrene bridge do not have a large influence on the electronic coupling between the two amine donor moieties and thus we prefer to classify compounds **7^+^** and **8^+^** as being at the borderline between class‐II and class‐III.

### DFT and TD‐DFT calculations

To rationalize the observed trends and properties we performed DFT and TD‐DFT calculations. The ground state structures were first optimized in the gas‐phase at the B3LYP/6‐31+G* level of theory. Previous studies^28^ have shown that range‐separated hybrid functionals are necessary to obtain a reliable picture of the nature and relative energetic ordering of the excited states in pyrenes. We have thus used the CAM‐B3LYP functional for the subsequent TD‐DFT calculations.

**Figure 8:**
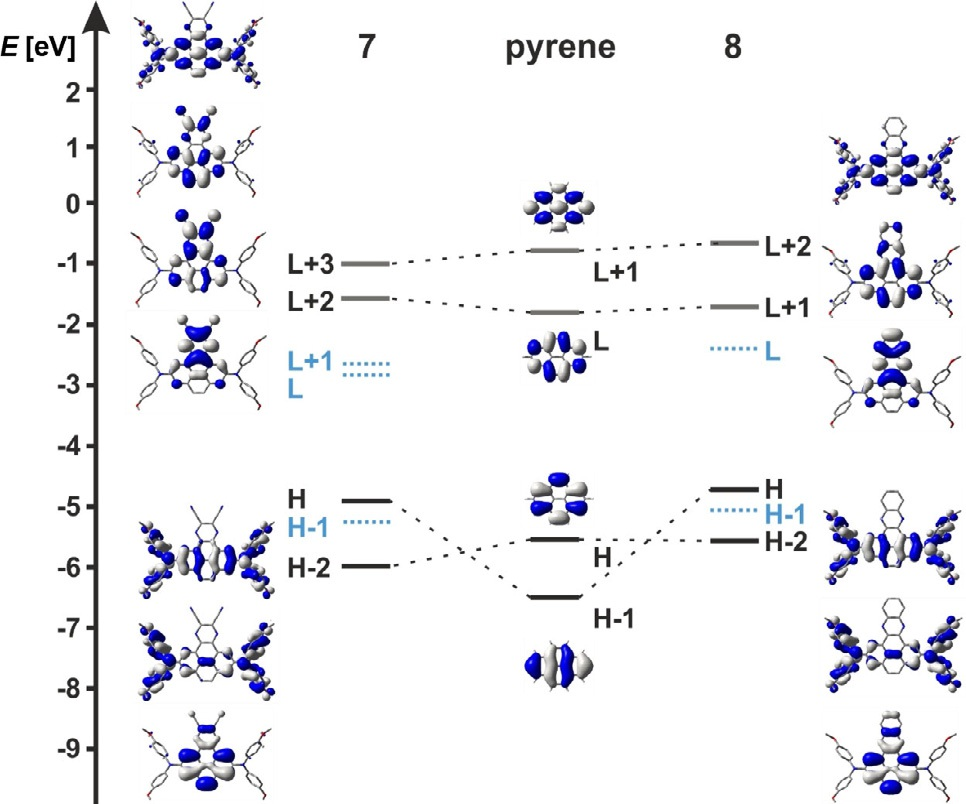
Molecular orbital diagram with orbital depictions of pyrene, **7** and **8**.

The nitrogen 2p_*z*_ orbitals in both pyrene derivatives **7** and **8** mix very efficiently with the HOMO‐1 of the pyrene core. This leads to a drastic destabilization of the HOMO−1 (black, Figure 8) by ca. 1.51 eV in **7** and 1.82 eV in **8,** which consequently switches the order of the HOMO and HOMO−1 orbitals. The pyrene‐like HOMO (black, Figure 8), on the other hand, mixes with the acceptor unit at the K‐region. Hence, the pyrene bridge and the acceptor unit are fully delocalized and thus, this orbital (now HOMO−2) is stabilized by around 0.54 eV in **7** and 0.08 eV in **8**. A new orbital (blue, Figure 8) of non‐bonding character with large coefficients at the nitrogens of the dianisylamine donors is energetically positioned between the new HOMO and HOMO−2 orbitals, which was also observed for derivative **IX**. The LUMOs of both **7** and **8** are greatly delocalized over the acceptor units and the pyrene bridge. Hence, these orbitals are considerably stabilized, with the LUMO of **7** stabilized by 1.08 eV, and of **8** by 0.52 eV compared to the one in pyrene. This pronounced stabilization of the LUMOs in **7** and **8** is reflected in our cyclic voltammetry studies by their remarkably low reduction potentials of −1.42 V and −1.84 V vs. Fc/Fc^+^, respectively. The pyrene LUMO and LUMO+1‐like orbitals (grey, Figure 8) are not significantly affected in **7** (now LUMO+2 and LUMO+3, respectively) and **8** (now LUMO+1 and LUMO+2, respectively). The pyrene LUMO‐like orbital is delocalized over the acceptor unit and the pyrene core, while the pyrene LUMO+1‐like one is delocalized over the dianisylamine donors and the pyrene core.

The TD‐DFT calculations show that the nature of the S_1_←S_0_ transition changes in such a way that it is no longer a nearly 50:50 weighted configuration interaction of HOMO−1→LUMO and HOMO→LUMO+1 as in pyrene (Tables 4 and 5). In **7** and **8**, the S_1_←S_0_ transitions are nearly pure HOMO→LUMO transitions, which have significant CT nature (Figure 8). They are strongly bathochromically shifted in the order **7** >**8** with low oscillator strengths, which matches well with the absorption maxima and extinction coefficients that were measured for this band (**7**: *λ*_max_ (abs)=542 nm, *ϵ*=3100 m ^−1^ cm^−1^ and **8**: *λ*_max_ (abs)=519 nm, *ϵ*=4000 m ^−1^ cm^−1^).

**Table 4:** TD‐DFT results of the compounds **7^+^**, **8^+^**, and **III^+^** using UBLYP with 35 % exact exchange admixture, an SVP basis set and a polarizable continuum model accounting for solvent effects (CH_2_Cl_2_).

	*S*_n_	*E* [eV] (*E* [nm])	*f*	Configuration (major contribution>10 %)
**7^+^**	S_1_	0.96 (1286)	0.383	β‐H→β‐L (97 %)
	S_2_	1.55 (799)	0.020	β‐H‐1→β‐L (94 %)
	S_3_	1.84 (675)	0.271	β‐H‐4→β‐L (97 %)
	S_4_	2.01 (616)	0.007	β‐H‐3→β‐L (18 %), α‐H→α‐L+1 (11 %), β‐H‐1→β‐L+2 (10 %)
	S_5_	2.13 (582)	0.137	β‐H‐3→β‐L (56 %), α‐H→α‐L+1 (14 %)
**8^+^**	S_1_	0.87 (1429)	0.505	β‐H→β‐L (97 %)
	S_2_	1.37 (902)	0.005	β‐H‐1→β‐L (95 %)
	S_3_	1.91 (651)	0.231	β‐H‐4→β‐L (91 %)
	S_4_	1.95 (637)	0.074	β‐H‐2→β‐L (68 %)
	S_5_	2.07 (599)	0.047	β‐H‐2→β‐L (15 %), α‐H‐2→α‐L+1 (11 %), α‐H→α‐L (11 %)
**III^+^**	S_1_	0.87 (1419)	0.478	β‐H→β‐L (97 %)
	S_2_	1.13 (1102)	0.003	β‐H‐1→β‐L (98 %)
	S_3_	1.92 (646)	0.265	β‐H‐4→β‐L (98 %)
	S_4_	1.98 (626)	0.033	β‐H‐3→β‐L (31 %), α‐H‐2→α‐L (27 %), β‐H‐1→β‐L+1 (23 %)
	S_5_	2.11 (587)	0.087	β‐H‐3→β‐L (53 %), α‐H‐2→α‐L (18 %)

**Table 5:** TD‐DFT results (CAM‐B3LYP/6–31+G*) for the five first vertical transitions of pyrene, **7** and **8**.

	FC‐S_n_	*E* [eV] (*E* [nm])	*f*	Configuration (major contribution >10 %)
pyrene	S_1_	3.99 (311)	0.000	H‐1→L (49 %), H→L+1 (50 %)
	S_2_	4.02 (309)	0.323	H→L (67 %), H‐1→L+1 (23 %)
	S_3_	4.90 (253)	0.000	H‐2→L (21 %), H→L+2 (67 %)
	S_4_	5.10 (243)	0.000	H‐2→L (66 %), H→L+2 (19 %)
	S_5_	5.11 (243)	0.414	H‐1→L (50 %), H→L+1 (49 %)
**7**	S_1_	2.69 (461)	0.071	H→L (90 %)
	S_2_	2.76 (449)	0.047	H→L+1 (84 %)
	S_3_	3.40 (365)	0.059	H‐1→L (66 %), H‐2→L (18 %)
	S_4_	3.43 (361)	0.005	H‐1→L+1 (79 %)
	S_5_	3.57 (348)	0.167	H‐2→L (72 %), H‐1→L (20 %)
**8**	S_1_	2.93 (423)	0.120	H→L (90 %)
	S_2_	3.19 (398)	0.006	H→L+1 (66 %), H‐2→L (12 %)
	S_3_	3.63 (342)	0.390	H‐2→L (75 %), H→L+1 (15 %)
	S_4_	3.64 (341)	0.002	H‐8→L (90 %)
	S_5_	3.76 (330)	0.000	H‐1→L (82 %)

## Conclusions

We have reported the synthesis and structural characterization of new pyrene derivatives with donor moieties at the 2,7‐positions and an acceptor group at the K‐region. In general, unsymmetrically substituted pyrene derivatives remain rare due to their challenging synthesis. The influence of the donors at the 2,7‐positions and the acceptors at the K‐region on the photophysical and electrochemical properties of the compounds is remarkable. The new derivatives possess very broad absorptions with maxima at 519–658 nm, and tails up to 800 nm, and compound **8** emits in the red to NIR region, which has not previously been reported for monomeric pyrenes to that extent. The intrinsic lifetimes remain rather long (*τ_0_*=104–293 ns), which is a typical property of 2,7‐substituted pyrenes, whereas the excited states of most K‐region substituted pyrenes have lifetimes (*τ_0_*) of only around 13–16 ns. Cyclic voltammetry studies reveal two reversible one‐electron oxidations and one reversible reduction for all three derivatives **6**, **7** and **8**, with very low potentials showing the unique donating and accepting properties of our derivatives. Spectroelectrochemical measurements suggest a strong electronic coupling between the substituents at the 2,7‐positions for **7** and **8**. Our DFT and TD‐DFT calculations indicate that these properties are the result of the very strong donors and acceptors which couple very well with the pyrene orbitals, resulting in a reordering of the occupied orbitals. Consequently, the S_1_ state of these derivatives has strong CT nature giving rise to unparalleled properties.

## Experimental Section

### General considerations

The catalyst precursors [Ir(COD)(OMe)]_2_ 68 and Pd_2_(dba)_3_⋅CHCl_3_ 69 were prepared according to literature procedures, B_2_pin_2_ was a gift from AllyChem Co. Ltd. while other starting materials were purchased from commercial sources and used as received. Solvents used for synthesis were HPLC grade, further treated to remove trace water using a commercial solvent purification system from Innovative Technology Inc. and deoxygenated using the freeze‐pump‐thaw method.

The ^1^H, ^13^C{^1^H}, ^11^B{^1^H}, ^15^N HMBC NMR spectra were recorded at room temperature in CDCl_3_ or CD_2_Cl_2_ solution either on a Bruker Avance I HD 500 (^1^H, 500 MHz; ^13^C, 125 MHz; ^11^B, 160 MHz), a Bruker Avance III HD 300 (^1^H, 300 MHz; ^13^C, 75 MHz; ^11^B, 96 MHz) or a Bruker Avance 200 (^1^H, 200 MHz). ^1^H NMR and ^13^C spectra are referenced to the residual protonated solvent CHCl_3_ (^1^H, *δ*=7.26, CDCl_3_) and CHDCl_2_ (^1^H, *δ*=5.32, CD_2_Cl_2_). ^11^B{^1^H} NMR signals were referenced to external BF_3_⋅OEt_2_ and ^15^N HMBC signals were referenced to MeNO_2_+10 %CDCl_3_. Chemical shifts are listed in parts per million (ppm) and coupling constants in Hertz (Hz).

HRMS were recorded using a Thermo Scientific Exactive Plus Orbitrap MS system with either an Atmospheric Sample Analysis Probe (ASAP) or by Electrospray Ionization (ESI). Elemental analyses were performed on an Elementar vario MICRO cube elemental analyzer.

Cyclic voltammetry experiments were performed using a Gamry Instruments Reference 600 potentiostat. A standard three‐electrode cell configuration was employed using a platinum disk working electrode, a platinum wire counter electrode, and a silver wire, separated by a *Vycor* tip, serving as the reference electrode. Formal redox potentials are referenced to the ferrocene/ferrocenium ([Cp_2_Fe]^+/0^) redox couple by using decamethylferrocene ([Cp*_2_Fe]; *E*_1/2_=−0.532 V in CH_2_Cl_2_) as an internal standard. Tetra‐*n‐*butylammonium hexafluorophosphate ([*n*Bu_4_N][PF_6_]) or [*n*Bu_4_N][Al(OC(CF_3_)_3_)_4_] were employed as supporting electrolytes. Compensation for resistive losses (*iR* drop) was employed for all measurements.

Crystals suitable for single‐crystal X‐ray diffraction were selected, coated in perfluoropolyether oil, and mounted on MiTeGen sample holders. Diffraction data were collected on a Bruker X8 Apex II 4‐circle diffractometer with a CCD area detector using Mo‐Kα radiation monochromated by graphite (**2**, **6′**, **11**, **12**) or multi‐layer focusing mirrors (**5′**, **10**). Diffraction data of **7** were collected on a Bruker D8 Quest 4‐circle diffractometer with a CMOS area detector (Photon II) and multi‐layer mirror monochromated Mo‐Kα radiation. The crystals were cooled using Oxford Cryostream or Bruker Kryoflex II low‐temperature devices. Data were collected at 100 K. The images were processed and corrected for Lorentz‐polarization effects and absorption as implemented in the Bruker software packages. The structures were solved using the intrinsic phasing method (SHELXT)^70^ and Fourier expansion technique. All non‐hydrogen atoms were refined in anisotropic approximation, with hydrogen atoms “riding” in idealized positions, by full‐matrix least squares against F^2^ of all data, using SHELXL^70^ software. In compound **11**, the coordinates of the hydrogen atom of the water molecule, which lies on a two‐fold rotation axis, were refined freely, but restraints were applied to the O‐H and H‐H distances. In compound **5′** the coordinates of the hydrogen atoms bonded to nitrogen were refined freely. The methoxyphenyl groups as well as the tetrahydrofuran solvent molecule are strongly disordered in compound **7**. Hence, several restraints had to be applied in the refinement. Diamond^71^ software was used for graphical representation. Hirshfeld surfaces were calculated and analyzed using the Crystal Explorer^72^ program. Other structural information was extracted using Mercury^73^ and OLEX2^74^ software. Crystal data and experimental details are listed in Table S1. CCDC https://www.ccdc.cam.ac.uk/services/strctures?id=doi:10.1002/chem.201904219 contain the supplementary crystallographic data for this paper. These data are provided free of charge by http://www.ccdc.cam.ac.uk/.

### General photophysical measurements

All photophysical measurements were carried out under an argon atmosphere. All solution state measurements were performed in standard quartz cuvettes (1 cm *x* 1 cm cross section). UV/Vis absorption spectra were recorded using an Agilent 1100 diode array UV/Vis spectrophotometer. Excitation, emission, lifetime and quantum yield measurements were recorded using an Edinburgh Instruments FLSP920 spectrophotometer equipped with a 450 W Xenon arc lamp, double monochromators for the excitation and emission pathways, and a red‐sensitive photomultiplier (PMT‐R928P) and a near‐IR PMT as detectors. The measurements were made in right‐angle geometry mode and all spectra were fully corrected for the spectral response of the instrument. All solutions used in photophysical measurements had a concentration lower than 10^−5^ m.

### Fluorescence quantum yield measurements

Fluorescence quantum yields of the samples were measured using a calibrated integrating sphere (150 mm inner diameter) from Edinburgh Instruments combined with the FLSP920 spectrophotometer described above. For solution‐state measurements, the longest wavelength absorption maximum of the compound in the respective solvent was chosen for the excitation. In order to exclude self‐absorption, the emission spectra were measured with dilute samples (ca. 0.1 OD at the excitation wavelength).

### Fluorescence lifetime measurements

Lifetime measurements were conducted using the time‐correlated single‐photon counting method (TCSPC) on the FLSP920 spectrophotometer equipped with a high‐speed photomultiplier tube positioned after a single emission monochromator. Measurements were made in right‐angle geometry mode, and the emission was collected through a polarizer set to the magic angle. Solutions were excited with either a 315 (pulse width 932.5 ps), 376 (pulse width 72.6 ps) or a 472 nm (pulse width 90.6 ps) pulsed diode laser at repetition rates of 1–5 MHz and were recorded at emission maxima. Decays were recorded to 10 000 counts in the peak channel with a record length of at least 4 000 channels. The band‐pass of the monochromator was adjusted to give a signal count rate of <20 kHz. Iterative reconvolution of the IRF with one decay function and nonlinear least‐squares analysis were used to analyze the data. The quality of all decay fits was judged to be satisfactory, based on the calculated values of the reduced *χ* ^2^ and Durbin‐Watson parameters and visual inspection of the weighted and autocorrelated residuals.

### Spectroelectrochemical measurements

Spectroelectrochemical experiments in reflection mode were performed using an Agilent Cary 5000 Spectrophotometer in combination with a designed sample compartment consisting of a cylindrical PTFE cell with an Infrasil wedge window with an angle of 0.5° and an adjustable three‐in‐one electrode (6 mm platinum disc working electrode, 1 mm platinum counter electrode and pseudo reference electrode). The potentials were adjusted with a Gamry 600 potentiostat and all experiments were performed at room temperature under an argon atmosphere.

### Theoretical studies

All calculations (DFT and TD‐DFT) were carried out with the Gaussian 09 (Rev. E.01) program package^75^ and were performed on a parallel cluster system. GaussView 5.0.9 was used to visualize the results, to measure calculated structural parameters, and to plot orbital surfaces (isovalue: ±0.02 [*e a*_0_^−3^]^1/2^). The ground‐state geometries were optimized using the B3LYP functional^76^ in combination with the 6‐31G(d) basis set.^77^ The optimized geometries were confirmed to be local minima by performing frequency calculations and obtaining only positive (real) frequencies. Based on these optimized structures, the lowest‐energy gas‐phase vertical transitions were calculated (singlets, 10 states) by TD‐DFT, using the Coulomb‐attenuated functional CAM‐B3LYP^78^ in combination with the 6‐31G+(d,p) basis set. DFT calculations on the monocations of **7** and **8** were carried out, using UBLYP with 35 % exact‐exchange admixture, the SVP basis set and a polarizable continuum model accounting for solvent effects. The time dependent (TD‐DFT) calculations were performed at the same level of theory.^65^, ^79^

### Synthesis of the pyrene derivatives

Pyrene‐4,5‐dione (**1**): In a round bottom flask pyrene (10.0 g, 49.4 mmol, 1.00 equiv) and RuCl_3_ **⋅** *x* H_2_O (987 mg, 4.74 mmol, 0.096 equiv) were dissolved in 200 mL of MeCN. NaIO_4_ (42.3 g, 198 mmol, 4.00 equiv) was dissolved in 250 mL of hot water and carefully added to the pyrene solution. Another 200 mL of CH_2_Cl_2_ were added and the reaction mixture was vigorously stirred for 18 h at room temperature. After filtration of the suspension through Celite, the filtrate was extracted with CH_2_Cl_2_ (3×100 mL) and the combined organic phases were washed with saturated aqueous Na_2_S_2_O_3_ and H_2_O and then dried over Na_2_SO_4._ After removing the solvent under reduced pressure the product was obtained as a dark orange solid (7.56 g, 66 %). The spectroscopic data match those reported previously.^47^, ^50^, ^80^ ^1^H NMR (200 MHz, CDCl_3_): *δ*=8.51 (dd, *J=*7 Hz, *J=*1 Hz, 2 H), 8.19 (dd, *J=*8 Hz, *J=*1 Hz, 2 H), 7.87 (s, 2 H), 7.77 (t, *J=*8 Hz, 2 H) ppm.

**Pyrene‐4,5‐di(ethyleneglycol)ketal (2)**: Pyrene‐4,5‐dione **(1)** (4.92 g, 21.2 mmol, 1.00 equiv) was suspended in 200 mL of toluene and ethylene glycol (148 mL, 2.65 mol, 125 equiv) and *p*‐toluenesulfonic acid (1.81 g, 9.53 mmol, 0.45 equiv) were added. The reaction mixture was refluxed at 125 °C for 20 h. Toluene was removed under reduced pressure and 400 mL of water. The crude product was isolated by filtration, washed with water and purified by flash chromatography (silica, cyclohexane/ethyl acetate 9:1). The product was isolated as a white solid (3.46 g, 10.8 mmol, 51 %). The spectroscopic data match those reported previously.^81^ ^1^H NMR (500 MHz, CD_2_Cl_2_): *δ*=7.98 (dd, *J=*7 Hz, *J=*1 Hz, 2 H), 7.97 (dd, *J=*8 Hz, *J=*1 Hz, 2 H), 7.82 (s, 2 H), 7.73 (dd, *J=*8 Hz, *J=*7 Hz, 2 H), 4.29 (br, 4 H), 3.75 (br, 4 H) ppm. ^13^C{^1^H} NMR (125 MHz, CD_2_Cl_2_): *δ*=133.5, 131.8, 129.2, 127.6, 127.1, 126.9, 124.6, 93.9, 62.3. HRMS (ASAP^+^): *m*/*z* calcd for [C_20_H_16_O_4_+H]^+^: 321.1121; found: [*M*+H]^+^ 321.1115 (IΔI=1.87 ppm). Elemental analysis calcd (%) for C_20_H_16_O_4_: C 74.99, H 5.03; found: C 74.90, H 5.08.

**2,7‐Bis(Bpin)‐4,5‐di(ethyleneglycol)ketal‐pyrene (3)**: In an argon‐filled glovebox, a Young's tube was filled with pyrene‐4,5‐di(ethyleneglycol)ketal **(2)** (2.91 g, 9.08 mmol, 1.00 equiv), B_2_pin_2_ (4.61 g, 18.2 mmol, 2.00 equiv), [Ir(COD)(OMe)]_2_ (301 mg, 0.45 mmol, 0.05 equiv), 4,4′‐di‐*tert*‐butyl‐2,2′‐dipyridine (dtbpy) (260 mg, 0.91 mmol, 0.10 equiv) and 50 mL of MTBE. The Young's tube was sealed, and the reaction mixture was stirred at 80 °C for 20 h. After cooling to room temperature, the crude product was passed through a pad of silica using toluene as the eluent. The solvent was removed under reduced pressure and the crude product was purified via flash chromatography (silica, cyclohexane/ethyl acetate 95:5). The product was obtained as a white solid (4.58 g, 8.00 mmol, 88 %). ^1^H NMR (300 MHz, CD_2_Cl_2_): *δ*=8.40 (d, *J=*1 Hz, 2 H), 8.28 (d, *J=*1 Hz, 2 H), 7.85 (s, 2 H), 4.33–4.28 (m, 4 H), 3.76–3.71 (m, 4 H), 1.41 (s, 24 H) ppm. ^13^C{^1^H} NMR (75 MHz, CD_2_Cl_2_): *δ*=136.6, 133.0, 131.6, 129.7, 128.6, 127.4, 94.0, 84.6, 62.3, 25.2 ppm (one C not observed, likely that attached to B). ^11^B{^1^H} NMR (96 MHz, CD_2_Cl_2_): *δ*=31.5 ppm. HRMS (ASAP^+^): *m*/*z* calcd for [C_32_H_38_B_2_O_8_+H]^+^ 573.2826; found: [*M*+H]^+^ 573.2799 (IΔI=4.71 ppm). Elemental analysis calcd (%) for C_32_H_38_B_2_O_8_: C 67.16, H 6.69; found: C 66.89, H 6.97.

**2,7‐Bis(bromo)‐4,5‐di(ethyleneglycol)ketal‐pyrene (4)**: The compound 2,7‐bis(Bpin)‐4,5‐di(ethyleneglycol)ketal‐pyrene **(3)** (4.58 g, 7.99 mmol, 1.00 equiv) was diluted in 100 mL of THF and heated to 50 °C. Afterwards, 80 mL of MeOH was added and the heating bath temperature was elevated to 90 °C. A solution of CuBr_2_ (8.90 g, 40.0 mmol, 6.00 equiv) in 80 mL of H_2_O was slowly added to this reaction mixture via a dropping funnel. The reaction mixture was stirred at 90 °C for 96 h and an orange solid precipitated. The precipitate was isolated by filtration and washed with water and EDTA (0.1 m) solution. The crude product was purified via flash chromatography (silica, hexane/ethyl acetate 4:1) to give a white solid (1.02 g, 2.13 mmol, 27 %). ^1^H NMR (500 MHz, CDCl_3_): *δ*=8.08 (s, 4 H), 7.71 (s, 2 H), 4.32 (br, 4 H), 3.78 (br, 4 H) ppm. ^13^C{^1^H} NMR (125 MHz, CDCl_3_): *δ*=135.0, 133.1, 131.5, 127.9, 127.1, 125.1, 121.9, 93.2, 62.1 ppm. HRMS (APCI^+^): *m*/*z* calcd for [C_20_H_14_Br_2_O_4_+H]^+^ 476.9332; found: [*M*+H]^+^ 476.9330 (IΔI=0.42 ppm).

**2,7‐Bis(DPA)‐4,5‐di(ethyleneglycol)ketal‐pyrene (5)**: In an argon‐filled glovebox, a Young's tube was filled with 2,7‐bis(bromo)‐4,5‐di(ethyleneglycol)ketal‐pyrene **(4)** (3.00 g, 6.27 mmol, 1.00 equiv), bis(4‐methoxyphenyl)amine (3.02 g, 13.2 mmol, 2.10 equiv), Pd_2_(dba)_3_ **⋅**CHCl_3_ (130 mg, 126 μmol, 0.02 equiv), XPhos (120 mg, 251 μmol, 0.04 equiv), KO*t*Bu (2.11 g, 18.8 mmol, 3.00 equiv) and toluene (200 mL), which was then sealed. Afterwards, the reaction mixture was stirred at 115 °C for 15 h. The solvent was removed under reduced pressure and the crude product was purified by flash chromatography (Biotage® KP‐NH, cyclohexane/CH_2_Cl_2_ 4:1 → 4:1). Product **(5)** was obtained as a yellow solid (2.83 g, 3.65 mmol, 58 %). ^1^H NMR (300 MHz, CD_2_Cl_2_): *δ*=7.49 (d, *J=*2 Hz, 2 H), 7.31 (s, 2 H), 7.24 (d, *J=*2 Hz, 2 H), 7.14–7.09 (m, 8 H), 6.90–6.86 (m, 8 H), 4.11 (br, 4 H), 3.80 (s, 12 H), 3.65 (br, 8 H) ppm. ^13^C{^1^H} NMR (75 MHz, CD_2_Cl_2_): *δ*=156.6, 147.6, 141.3, 133.3, 132.0, 127.0, 126.8, 121.3, 119.2, 118.0, 115.1, 93.9, 62.1, 55.9 ppm. ^15^N (from ^15^N ^1^H HMBC) NMR (300 MHz, CD_2_Cl_2_): −285.8 ppm. HRMS (APCI^+^): *m*/*z* calcd for [C_48_H_42_N_2_O_8_+H]^+^ 775.3014; found: [*M*+H]^+^ 775.3000 (|Δ|=1.80 ppm). Elemental analysis calcd (%) for C_48_H_42_N_2_O_8_: C 74.40, H 5.46, N 3.62; found: C 74.00, H 6.07, N 3.34.

**2,7‐Bis(DPA)‐4,5‐dione (6)**: In a round bottom flask, 2,7‐bis(DPA)‐4,5‐di(ethyleneglycol)ketal‐pyrene **(5)** (500 mg, 645 μmol) was suspended in 10 mL of water. Then, 60 mL of TFA was added dropwise and the reaction mixture was stirred at room temperature for 15 h. The solution was then neutralized with an aqueous solution of NaHCO_3_ and extracted with ethyl acetate. The organic phases were dried over NaSO_4_, the resulting crude product was washed with methanol and the solvents were removed under reduced pressure. The product 2,7‐bis(DPA)‐4,5‐dione **(6)** was obtained as a dark green solid (360 mg, 534 μmol, 81 %). ^1^H NMR (300 MHz, CD_2_Cl_2_): *δ*=7.88 (d, *J=*3 Hz, 2 H), 7.43 (d, *J=*3 Hz, 2 H), 7.39 (s, 2 H), 7.15–7.10 (m, 8 H), 6.93–6.88 (m, 8 H), 3.82 (s, 12 H) ppm. ^13^C{^1^H} NMR (75 MHz, CD_2_Cl_2_): *δ*=181.1, 157.2, 148.1, 140.2, 132.9, 130.3, 127.5, 127.2, 123.5, 123.1, 122.7, 115.4, 55.9 ppm. ^15^N (from ^15^N ^1^H HMBC) NMR (300 MHz, CD_2_Cl_2_): −284.9 ppm. HRMS (ASAP^+^): *m*/*z* calcd for [C_44_H_34_N_2_O_6_+H]^+^ 687.2417; found [*M*+H]^+^ 687.2479 (|Δ|=9.02 ppm). Elemental analysis calcd (%) for C_44_H_34_N_2_O_6_: C 76.95, H 4.99, N 4.08; found: C 77.00, H 4.96, N 4.25.

**2,7‐Bis(DPA)‐4,5‐azaacene‐CN (7)**: In a round bottom flask, 2,7‐bis(DPA)‐4,5‐dione **(6)** (200 mg, 291 μmol), 2,3‐diaminomaleonitrile (188 mg, 1.74 mmol), ethanol (35 mL) and acetic acid (35 mL) were stirred at 80 °C for 15 h. The solution was then neutralized with an aqueous solution of NaHCO_3_. The precipitate was collected via filtration and washed with water and ethanol. Product **(7)** was obtained as a black solid (134 mg, 176 μmol, 60 %). ^1^H NMR (300 MHz, CD_2_Cl_2_): *δ*=8.74 (d, *J=*2 Hz, 2 H), 7.80 (d, *J=*2 Hz, 2 H), 7.67 (s, 2 H), 7.21–7.16 (m, 8 H), 6.93–6.88 (m, 8 H), 3.83 (s, 12 H) ppm. ^13^C{^1^H} NMR (75 MHz, CD_2_Cl_2_): *δ*=157.0, 148.2, 143.9, 140.9, 132.0, 130.1, 127.7, 127.1, 126.9, 122.8, 121.8, 117.5, 115.4, 114.4, 55.9 ppm. HRMS (APCI^+^): *m*/*z* calcd for [C_48_H_34_N_6_O_4_+H]^+^ 759.2714; found: [*M*+H]^+^ 759.2699 (|Δ|=1.98 ppm). Elemental analysis calcd (%) for C_48_H_34_N_6_O_4_: C 75.98, H 4.52, N 11.08; found: C 75.73, H 4.74, N 10.73

**2,7‐Bis(DPA)‐4,5‐azaacene‐Ph (8)**: In a round bottom flask, 2,7‐bis(DPA)‐4,5‐dione **(6)** (200 mg, 291 μmol), 1,2‐diamino‐benzene (189 mg, 1.74 mmol), ethanol (35 mL) and acetic acid (35 mL) were stirred at 80 °C for 15 h. The solution was then neutralized with an aqueous solution of NaHCO_3_. The precipitate was collected via filtration and washed with water and ethanol. Product **(8)** was obtained as a red solid (120 mg, 158 μmol, 54 %). ^1^H NMR (500 MHz, CDCl_3_): *δ*=9.17 (d, *J=*2, 2 H), 8.25–8.20 (m, 2 H), 7.79–7.74 (m, 2 H), 7.69 (d, *J=*2 Hz, 2 H), 7.65 (s, 2 H), 7.24–7.17 (m, 8 H), 9.94–6.86 (m, 8 H), 3.84 (s, 12 H) ppm. ^13^C NMR (125 MHz, CDCl_3_): *δ*=156.0, 147.2, 143.4, 142.3, 141.6, 132.0, 130.0, 129.7, 129.6, 127.1, 126.5, 121.6, 121.3, 118.1, 115.0, 55.7 ppm. HRMS (APCI^+^): *m*/*z* calcd for [C_50_H_38_N_4_O_4_+H]^+^ 759.2966; found: [*M*+H]^+^ 759.2963 (|Δ|=0.40 ppm). Elemental analysis calcd (%) for C_50_H_38_N_4_O_4_: C 79.14, H 5.05, N 7.38; found: C 78.82, H 5.07, N 7.53.

### Syntheses from the initially planned route

**2,7‐Bis(azido)‐4,5‐di(ethyleneglycol)ketal‐pyrene (4’)**: Compound **3** (6.62 g, 11.6 mmol, 1.00 equiv), NaN_3_ (2.26 g, 34.8 mmol) and Cu(OAc)_2_ (232 mg, 1.16 mmol) were suspended in 400 mL of MeOH and stirred in a preheated oil bath at 55 °C for 48 h. The green precipitate was collected and washed with EDTA solution, water and MeOH. The crude product was purified via column chromatography (silica, hexane/ethyl acetate 4:1) giving the product **4′** as a pale yellow solid (2.58 g, 55 %). ^1^H NMR (200 MHz, CD_2_Cl_2_): *δ*=7.75 (s, 2 H), 7.64 (d, *J=*2 Hz, 2 H), 7.56 (d, *J=*2 Hz, 2 H), 4.32–4.24 (m, 4 H), 3.76–3.68 (m, 4 H) ppm. ^13^C{^1^H} NMR (125 MHz, CD_2_Cl_2_): *δ*=139.7, 135.6, 132.7, 127.5, 124.0, 118.0, 116.8, 93.5, 62.3 ppm. ^15^N (from ^15^N ^1^H HMBC) NMR (500 MHz, CD_2_Cl_2_): *δ*=−288.1 ppm. HRMS (ASAP^+^): *m*/*z* calcd for [C_20_H_14_N_6_O_4_+H]^+^ 403.1149; found: [*M*+H]^+^ 403.1145 (|Δ|=0.99 ppm).

**2,7‐Bis(amino)‐4,5‐di(ethyleneglycol)ketal‐pyrene (5’)**: To a Schlenk flask, **4′** (1.03 g, 2.56 mmol) and 10 % Pd/C (37.6 mg) were added under an inert atmosphere. After the addition of 200 mL of MeOH, the reaction mixture was frozen in liquid N_2_. Successively, the flask was evacuated and refilled with an excess of hydrogen and evacuated again. The procedure was repeated two more times and, after the last hydrogen flush was performed, the reaction mixture was thawed and stirred at r.t. for 16 h. CH_2_Cl_2_ was added to the crude product and the suspension was then filtered. Afterwards, the solvent was removed under reduced pressure and the crude product was purified via column chromatography (silica, hexane/ethyl acetate 3:7). The final product **5′** was obtained as a pale yellow solid (440 mg, 49 %). ^1^H NMR (300 MHz, CD_3_CN): *δ*=7.47 (s, 2 H), 7.25 (d, *J=*2 Hz, 2 H), 7.00 (d, *J=*2 Hz, 2 H), 4.41 (s, 4 H), 4.20–4.15 (m, 4 H), 3.69 (br, 4 H) ppm. ^13^C{^1^H} NMR (75 MHz, CD_3_CN): *δ*=147.0, 134.0, 132.3, 126.8, 120.2, 114.8, 111.2, 94.6, 62.6 ppm. ^15^N (from ^15^N ^1^H HMBC) NMR (300 MHz, CD_3_CN): *δ*=−324.1. HRMS (ASAP^+^): *m*/*z* calcd for [C_20_H_18_N_2_O_4_+H]^+^ 351.1339; found: [*M*+H]^+^ 351.1324 (|Δ|=4.27 ppm). Elemental analysis calcd (%) for C_20_H_18_N_2_O_4_: C 68.56, H 5.18, N 8.00; found: C 68.51, H 5.18, N 7.26.

**2,7‐Bis(amino)‐*N*** ^**2**^,***N*** ^**2**^,***N*** ^**7**^,***N*** ^**7**^ **‐tetrakis(3‐methylbut‐2‐en‐1‐yl)‐4,5‐di(ethyleneglycol)ketal‐pyrene (6’)**: To a Young's tube, compound **5′** (686 mg, 1.96 mmol), K_2_CO_3_ (1.35 g, 9.80 mmol), molecular sieves 4 Å (0.50 g) and 1‐chloro‐3‐methylbut‐2‐ene (927 μL, 8.23 mmol) were suspended in 60 mL MeCN. The tube was sealed, and the reaction mixture was stirred at 80 °C in an oil bath for 48 h. After cooling to r.t., the precipitate was collected by filtration and washed with ethyl acetate. The solvent was removed from the filtrate under reduced pressure and the remaining brown oil was purified by column chromatography (silica, hexane/ethyl acetate 96:4), which yielded product **6′** as a yellow solid (518 mg, 42 %). ^1^H NMR 500 MHz, CD_2_Cl_2_): *δ*=7.49 (s, 2 H), 7.38 (s, 2 H), 7.00 (s, 2 H), 5.30 (m, 4 H), 4.22 (br, 4 H), 4.03 (d, *J=*6 Hz, 8 H), 3.74 (br, 4 H), 1.79 (s, 12 H), 1.76 (s, 12 H) ppm. ^13^C{^1^H} NMR (125 MHz, CD_2_Cl_2_): *δ*=147.6, 134.8, 132.6, 131.7, 126.8, 122.1, 118.6, 112.9, 110.1, 94.5, 62.3, 49.1, 25.9, 18.1 ppm. HRMS (ASAP^+^): *m*/*z* calcd for [C_40_H_50_N_2_O_4_+H]^+^ 623.3843; found [*M*+H]^+^ 623.3839 (|Δ|=0.64 ppm).

## Conflict of interest

The authors declare no conflict of interest.

## Supporting information

As a service to our authors and readers, this journal provides supporting information supplied by the authors. Such materials are peer reviewed and may be re‐organized for online delivery, but are not copy‐edited or typeset. Technical support issues arising from supporting information (other than missing files) should be addressed to the authors.

Supplementary
