# Supplementary material for: Synthesis, Photophysical and Electronic Properties of New Red‐to‐NIR Emitting Donor–Acceptor Pyrene Derivatives
Source: Chemistry. 2019 Nov 19;26(2):438–53. doi: 10.1002/chem.201904219 (PMC6973242; doi:10.1002/chem.201904219)
Supplement: Supplementary file 1 — Supplementary [file CHEM-26-438-s001.pdf]

CHEMISTRY
A European Journal

Supporting Information

Synthesis, Photophysical and Electronic Properties of New Red-to-NIR Emitting Donor–Acceptor Pyrene Derivatives

Julia Merz,[a] Maximilian Dietz,[a] Yvonne Vonhausen,[a] Frederik Wöber,[a] Alexandra Friedrich,[a] Daniel Sieh,[a] Ivo Krummenacher,[a] Holger Braunschweig,[a] Michael Moos,[b] Marco Holzapfel,[b] Christoph Lambert,[b] and Todd B. Marder*[a]

chem_201904219_sm_miscellaneous_information.pdf

Electronic Supporting Information

Contents

Synthesis of the derivatives **9-15**	S1-S2
NMR Spectra	S3-S20
Single-crystal X-ray diffraction	S21-S34
Orbital Depictions of Compound **6**, **7’** and **15**	S35
TD-DFT Results	S36-S39
Cartesian Coordinates	S40-S48
References	S49

Synthesis of der derivatives 9-14

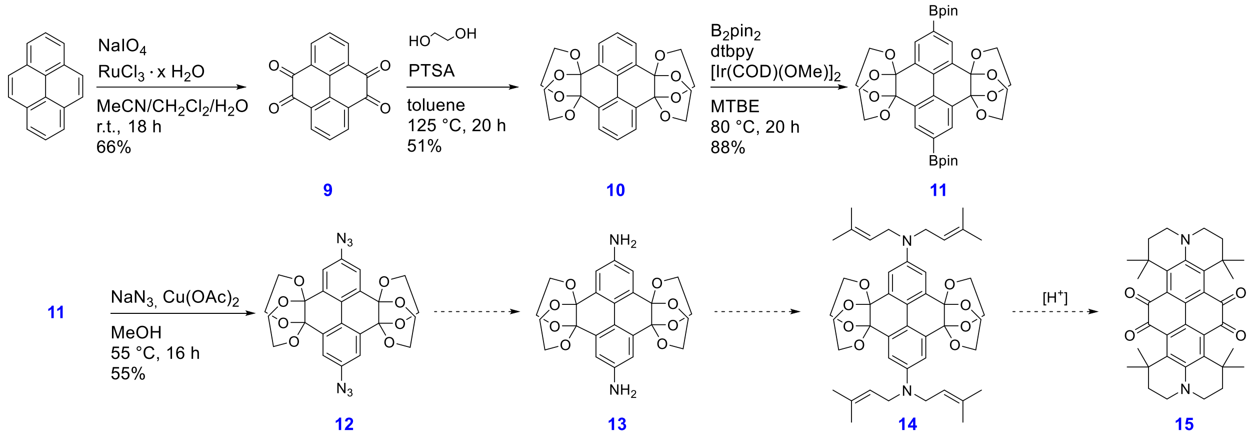

Scheme S1. Synthesis of the derivatives **9-14**.

Pyrene-4,5,9,10-tetra(ethyleneglycol)ketal (10)

In a round bottom flask fitted with a reflux condenser, pyrene-4,5,9,10-tetraone (**9**) (3.12 g, 11.9 mmol, 1.00 eq) was suspended in toluene (400 mL) followed by the addition of ethylene glycol (166 mL, 2.96 mol, 249.00 eq) and *p*-toluenesulfonic acid (2.04 g, 10.7 mmol, 0.90 eq). The reaction mixture was refluxed at 125 °C for 20 h. Toluene was removed under reduced pressure and 400 mL of water was added to the mixture. The crude product was isolated by filtration, washed with water and further purified by flash chromatography (silica, toluene/CH_2Cl_2/NEt_3 1:1:0.1) to give the title compound as a white solid (3.46 g, 67%).

^{1}H NMR (500 MHz, CD_2Cl_2): δ = 7.75 (d, *J* = 8 Hz, 4 H), 7.51 (t, *J* = 8 Hz, 2 H), 4.18 (br, 8 H), 3.65 (br, 8 H) ppm.

^{13}C{^{1}H} NMR (125 MHz, CD_2Cl_2): δ = 133.4, 129.8, 129.1, 127.6, 92.8, 62.3 ppm.

HRMS (ASAP$^+$): *m/z* calcd. for $[C_{24}H_{22}O_8+H]^+$ 439.1387; found $[M+H]^+$ 439.1365 (|Δ| = 5.00 ppm).

Elemental analysis calcd. (%) for $C_{24}H_{22}O_8$: C 65.75, H 5.06; found: C 65.88, H 5.25.

2,7-Bis(Bpin)-4,5,9,10-tetra(ethyleneglycol)ketal-pyrene (11)

In an argon-filled glovebox, pyrene-4,5,9,10-tetra(ethyleneglycol)ketal (**10**) (4.21 g, 9.60 mmol, 1.00 eq), B_2pin_2 (5.35 g, 21.1 mmol, 2.20 eq), $[Ir(COD)(OMe)]_2$ (318 mg, 480 mmol, 0.05 eq), dtbpy (275 mg, 960 mmol, 0.10 eq) and 40 mL THF were added to a Young's tube. After the tube was sealed and taken out of the glovebox, the reaction mixture was heated at 70 °C for 18 h. After cooling to room temperature, the mixture was passed through a pad of silica using CH_2Cl_2/NEt_3 (95:5). The solvent was removed under reduced pressure and the crude product was purified by dissolving it in CH_2Cl_2 and precipitating it with hexane (4.17 g, 63%).

^{1}H NMR (300 MHz, CD_2Cl_2): δ = 8.11 (s, 4H), 4.21 (br, 8 H), 3.65 (br, 8 H), 1.36 (s, 24 H).

^{13}C{^{1}H} NMR (75 MHz, CD_2Cl_2): δ = 133.7, 132.9, 131.4, 92.9, 84.5, 62.0, 25.1 (one C not observed, likely that attached to B).

^{11}B{^{1}H} NMR (96 MHz, CD_2Cl_2): δ = 30.6.

HRMS (ASAP$^+$): *m/z* calcd. for $[C_{36}H_{44}B_2O_{12}+H]^+$ 691.3092; found $[M+H]^+$ 691.3065 (|Δ| = 3.91 ppm).

Elemental analysis calcd. (%) for $C_{36}H_{44}B_2O_{12}$: C 62.63, H 6.42; found: C 62.39, H 6.58.

2,7-Bis(azido)-4,5,9,10-tetra(ethyleneglycol)ketal-pyrene (12)

Compound **11** (4.17 g, 6.04 mmol, 1.00 eq), NaN_3 (1.18 g, 18.1 mmol, 3.00 eq) and $Cu(OAc)_2$ (121 mg, 604 µmol, 0.10 eq) were suspended in MeOH (700 mL) and stirred at 55 °C for 48 h. The green precipitate was collected by filtration and washed with an EDTA (0.1 M) solution, water and MeOH. The crude product was purified *via* column chromatography (silica, hexane/EtOAc 3:1) giving the desired compound as a pale yellow solid (1.67 g, 53%).

^{1}H NMR (200 MHz, CD_2Cl_2): δ = 7.41 (s, 4 H), 4.20 (br, 8 H), 3.66 (br, 8 H) ppm.

^{13}C{^{1}H} NMR (125 MHz, CD_2Cl_2): δ = 141.8, 135.3, 125.5, 118.2, 92.5, 62.0 ppm.

^{15}N NMR (500 MHz, CD_2Cl_2): δ = -287.0 ppm.

HRMS (ASAP$^+$): *m/z* calcd. for 521.1415 $[C_{24}H_{20}N_6O_8+H]^+$; found 521.1415 $[M+H]^+$ (|Δ| = 0.00 ppm).

Spectroelectrochemistry

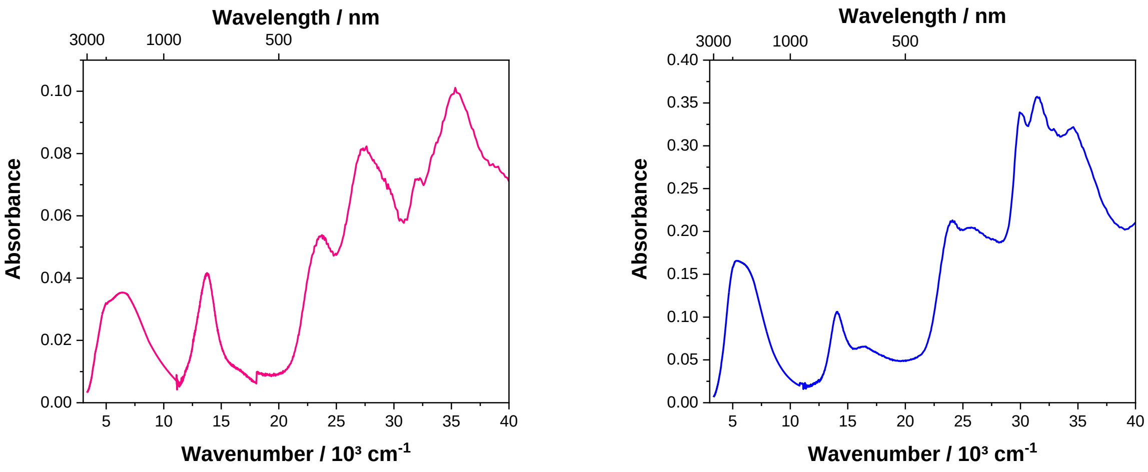

Figure S1. Absorption spectra of **7**$^+$ (left) and **8**$^+$ (right) obtained *via* spectroelectrochemical measurements in CH_2Cl_2/0.1 M [*n*-Bu_4N][PF_6].

NMR Spectra

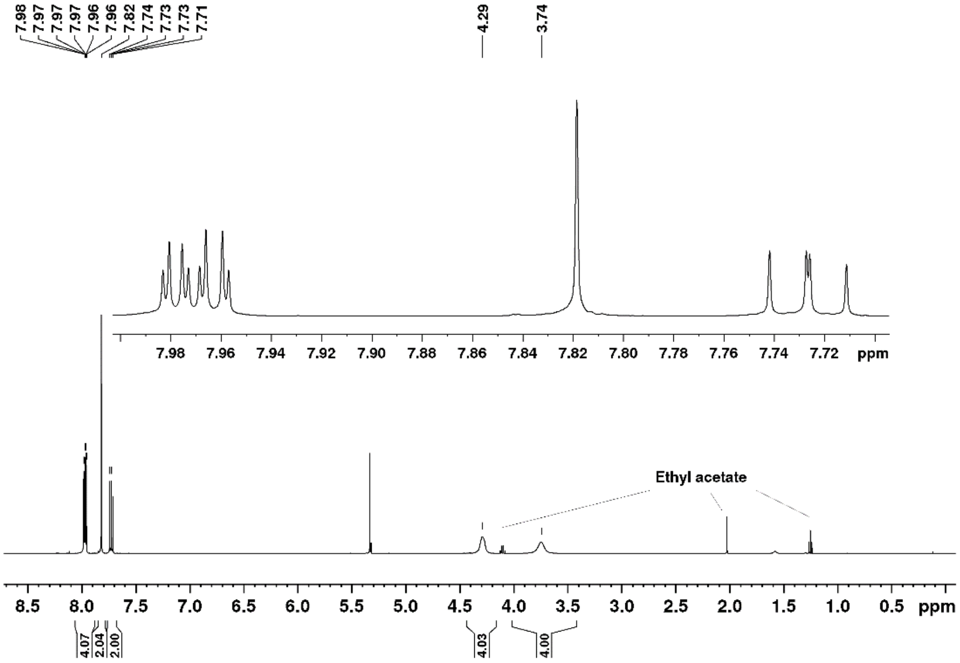

Figure S2. ^{1}H NMR spectrum of compound **2** recorded in CD_2Cl_2 at 500 MHz.

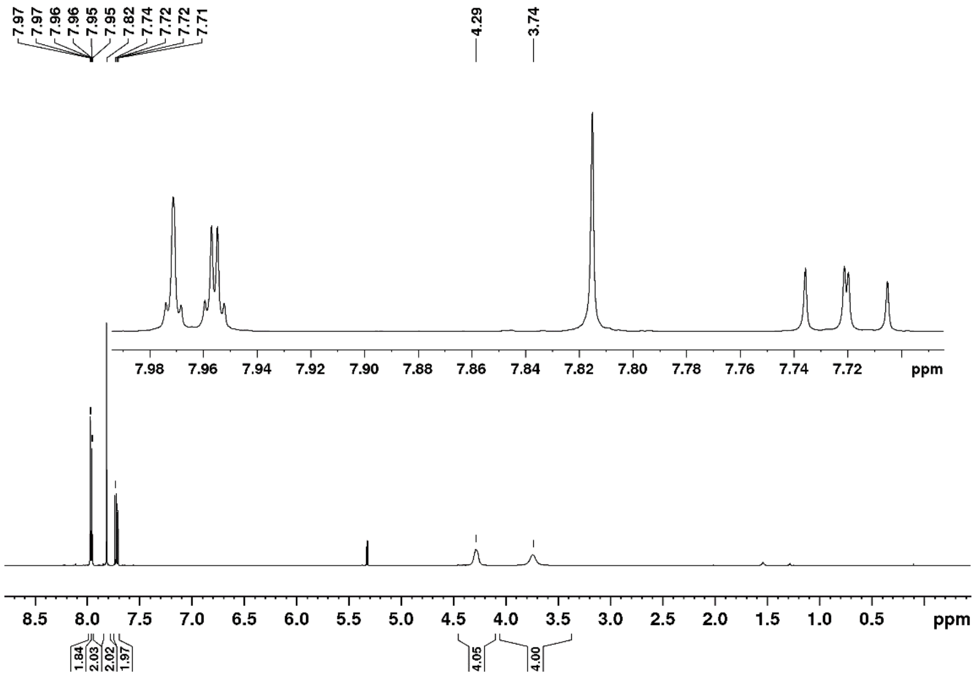

Figure S3. ^{1}H NMR spectrum of compound **2** recorded in CD_2Cl_2 at 500 MHz.

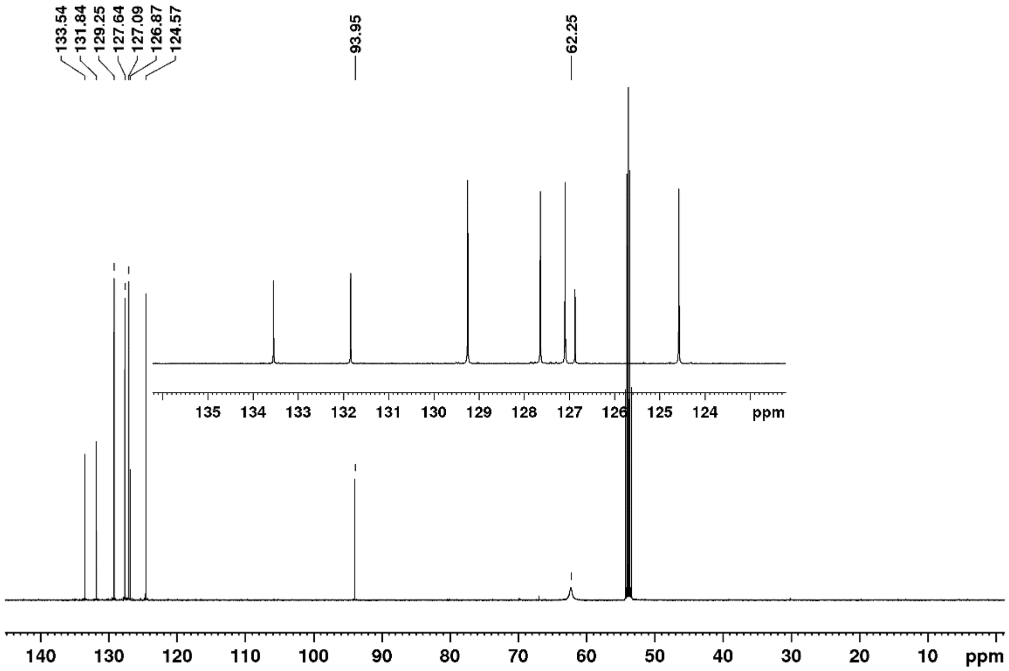

Figure S4. $^{13}C\{^{1}H\}$ NMR spectrum of compound **2** recorded in CD_2Cl_2 at 125 MHz.

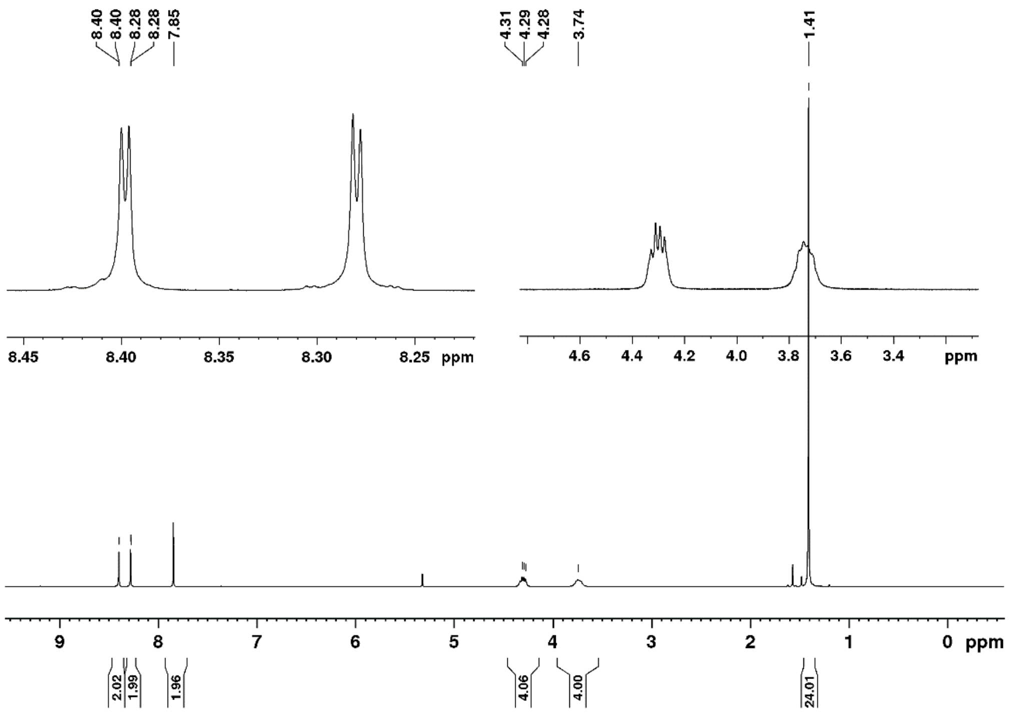

Figure S5. ^{1}H NMR spectrum of compound **3** recorded in CD_2Cl_2 at 300 MHz.

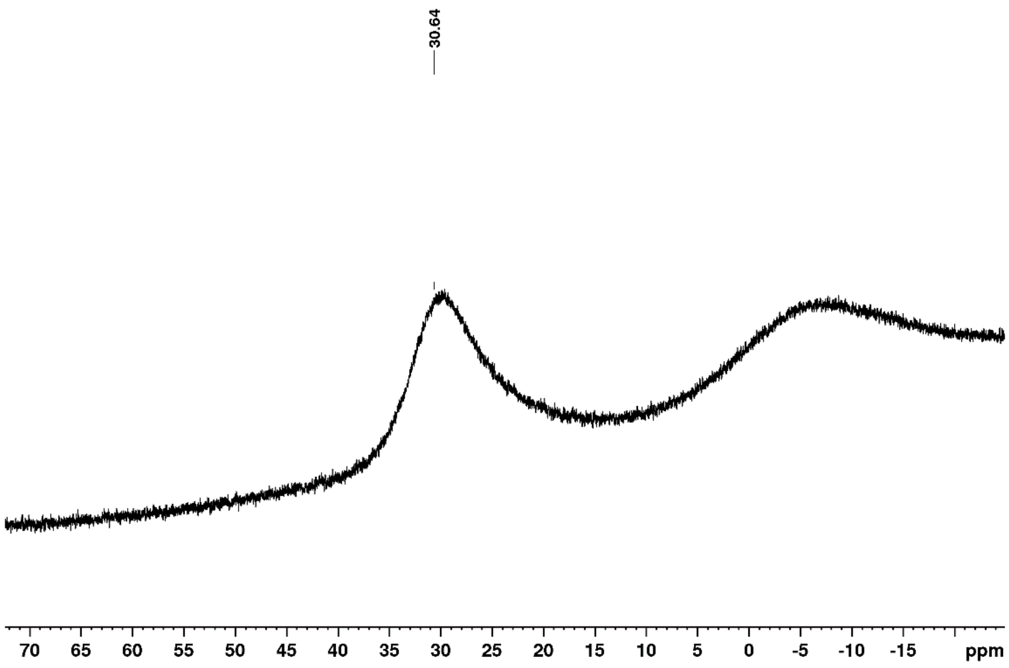

Figure S6. $^{11}B\{^{1}H\}$ NMR spectrum of compound **3** recorded in CD_2Cl_2 at 96 MHz.

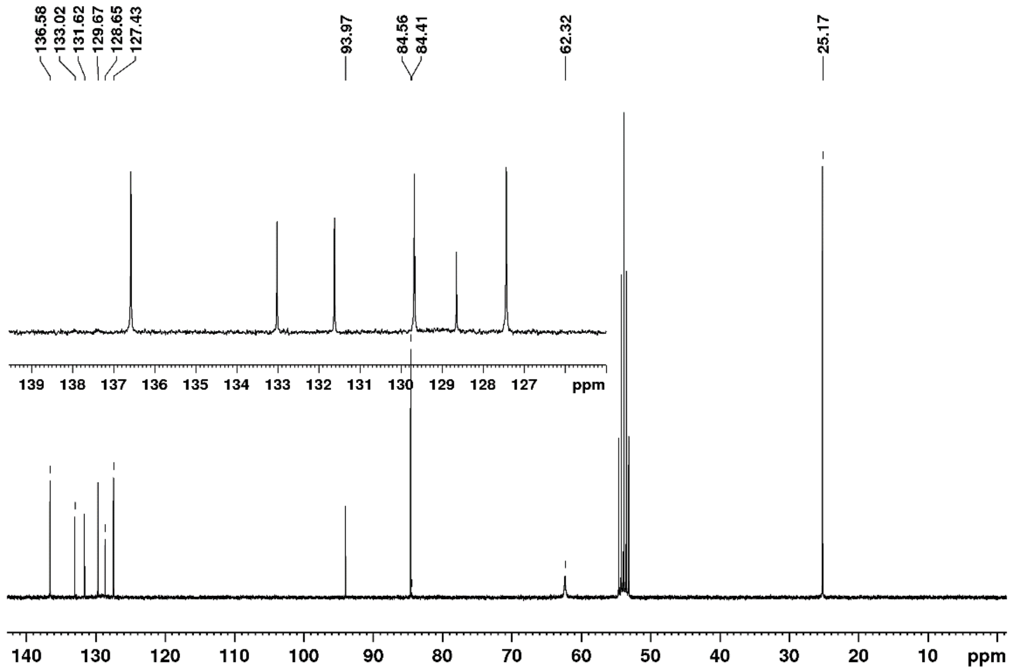

Figure S7. $^{13}C\{^1H\}$ NMR spectrum of compound **3** recorded in CD_2Cl_2 at 75 MHz.

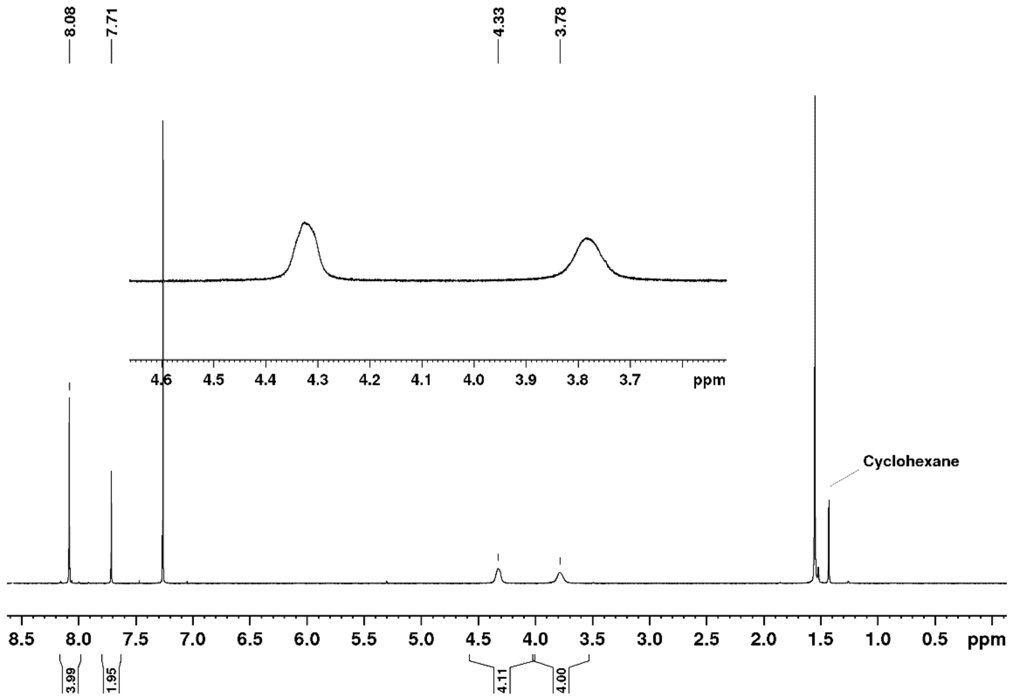

Figure S8. ^1H NMR spectrum of compound **4** recorded in $CDCl_3$ at 500 MHz.

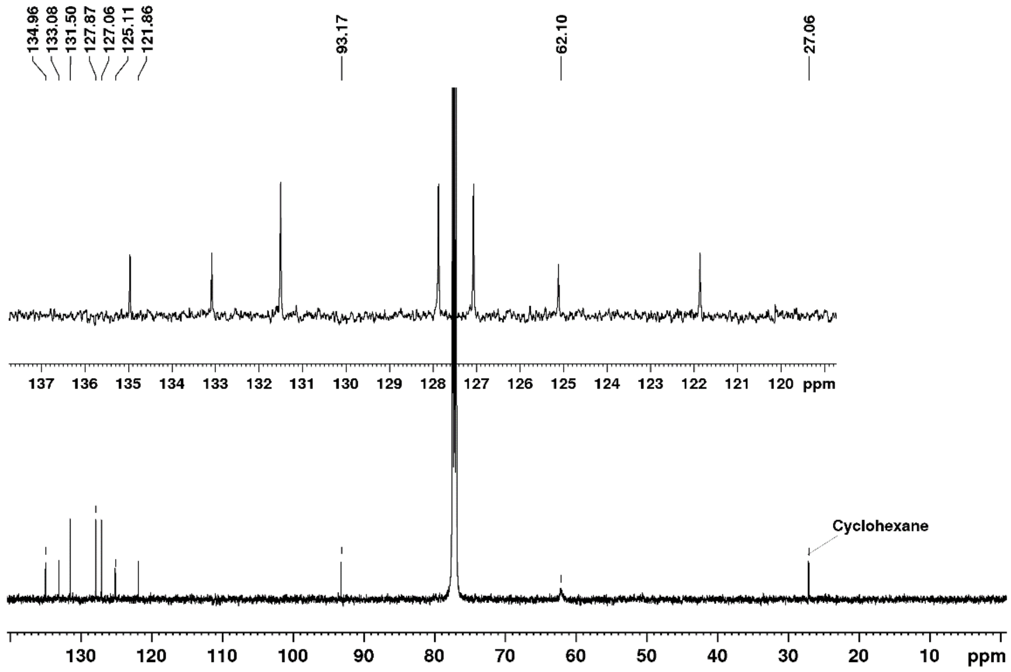

Figure S9. $^{13}C\{^1H\}$ NMR spectrum of compound **4** recorded in CD_2Cl_2 at 125 MHz.

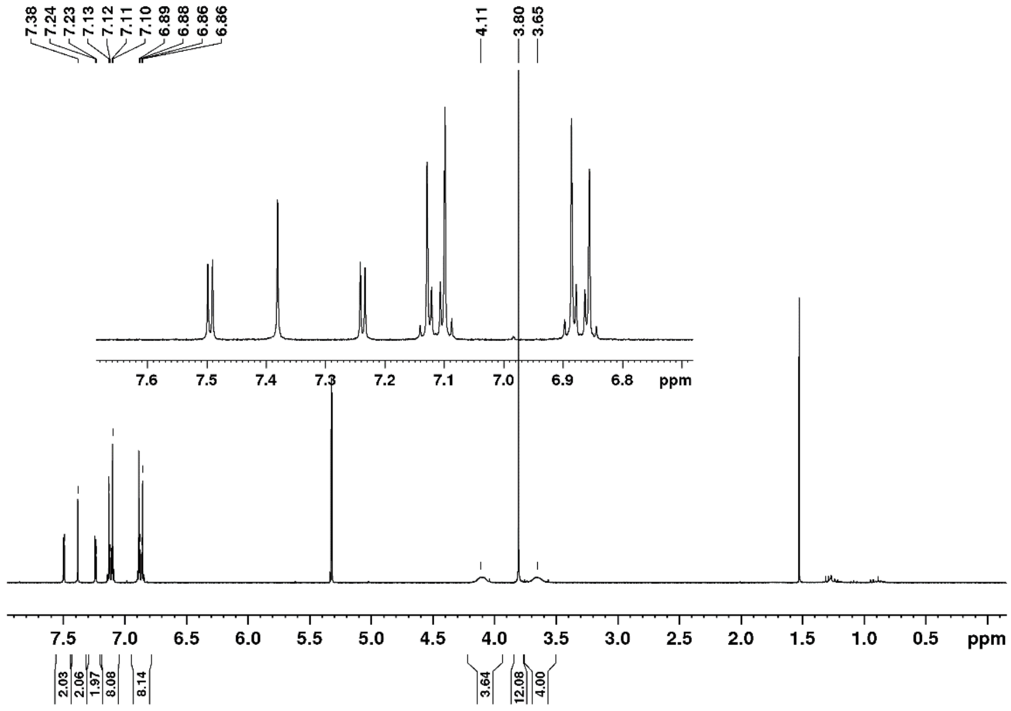

Figure S10. ^1H NMR spectrum of compound **5** recorded in CD_2Cl_2 at 500 MHz.

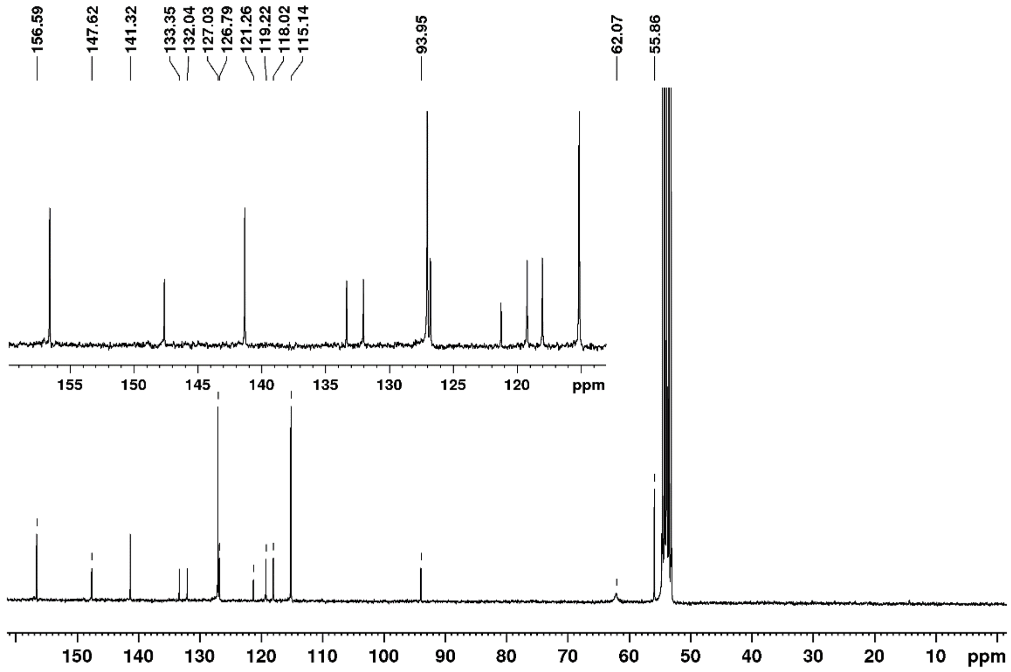

Figure S11. $^{13}C\{^{1}H\}$ NMR spectrum of compound **5** recorded in CD_2Cl_2 at 125 MHz.

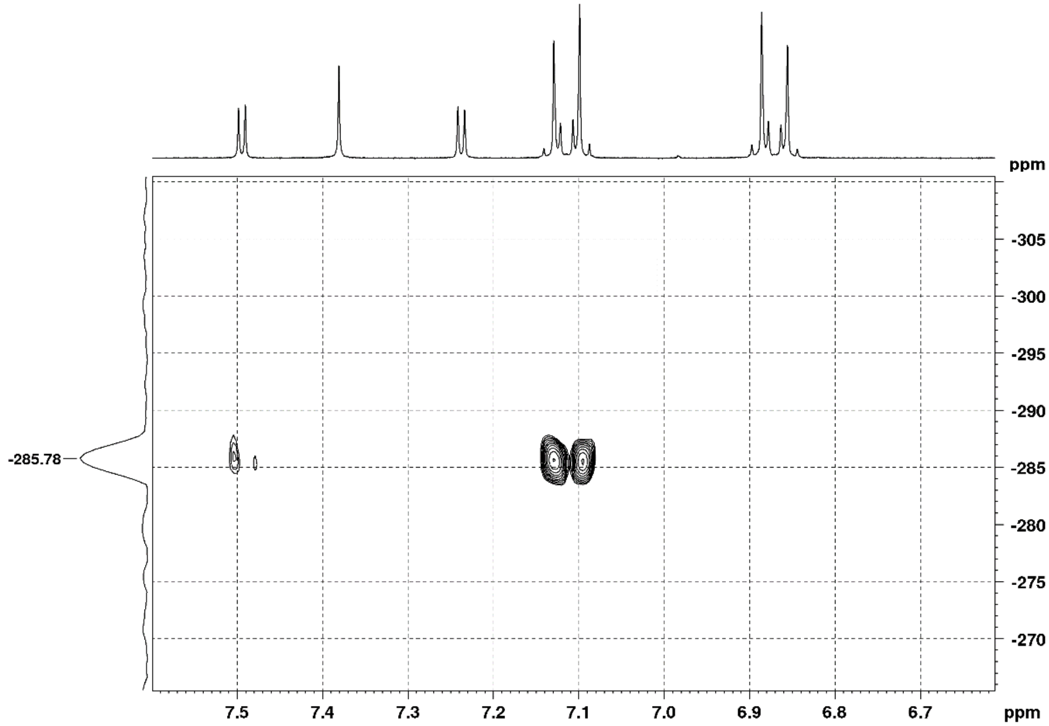

Figure S12. ^{15}N ^{1}H HMBC NMR spectrum of compound **5** recorded in CD_2Cl_2 at 500 MHz.

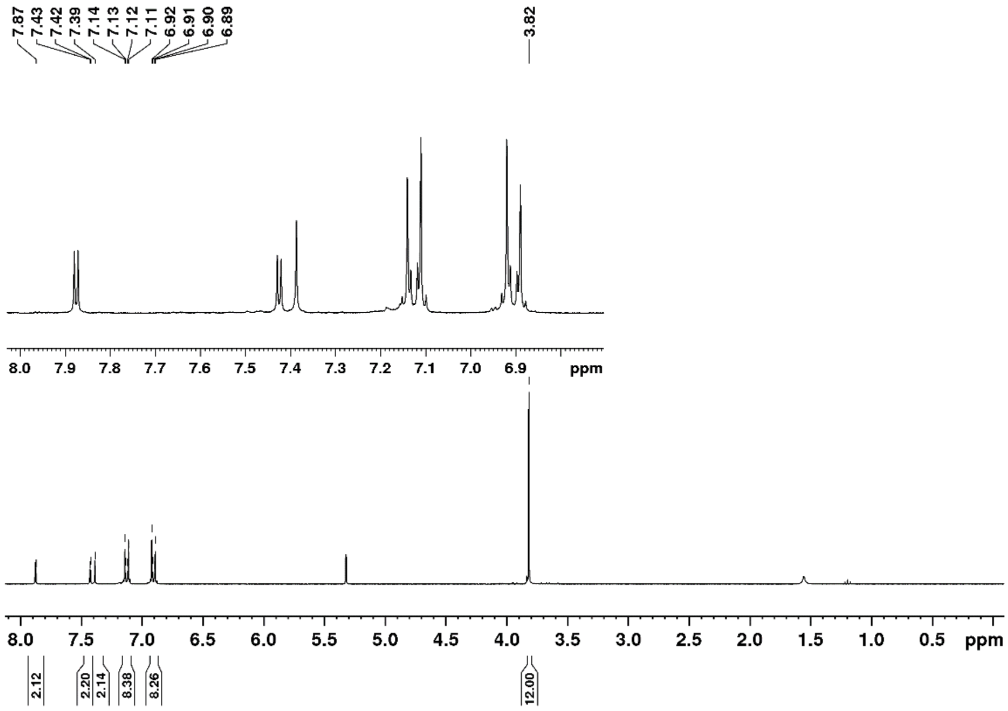

Figure S13. ^{1}H NMR spectrum of compound **6** recorded in CD_2Cl_2 at 500 MHz.

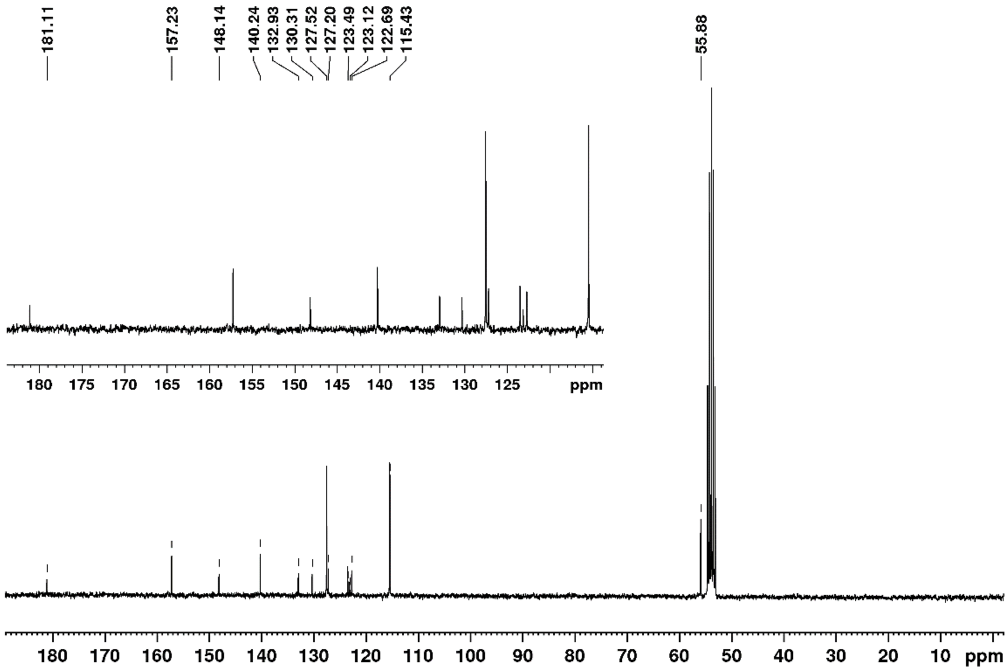

Figure S14. $^{13}C\{^{1}H\}$ NMR spectrum of compound **6** recorded in CD_2Cl_2 at 125 MHz.

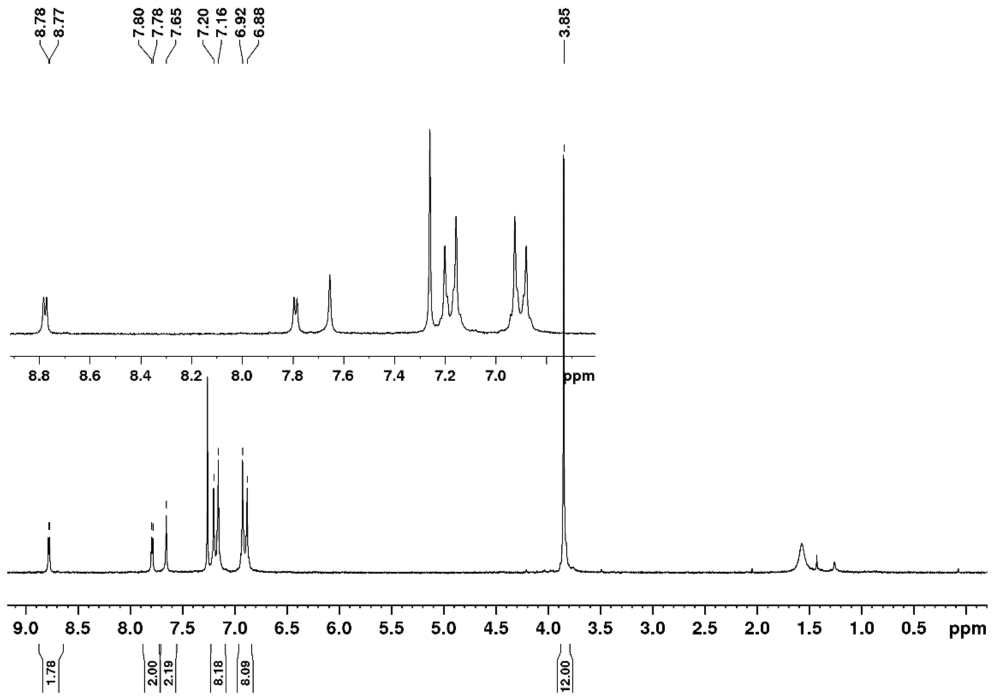

Figure S15. ^{1}H NMR spectrum of compound **7** recorded in $CDCl_3$ at 200 MHz.

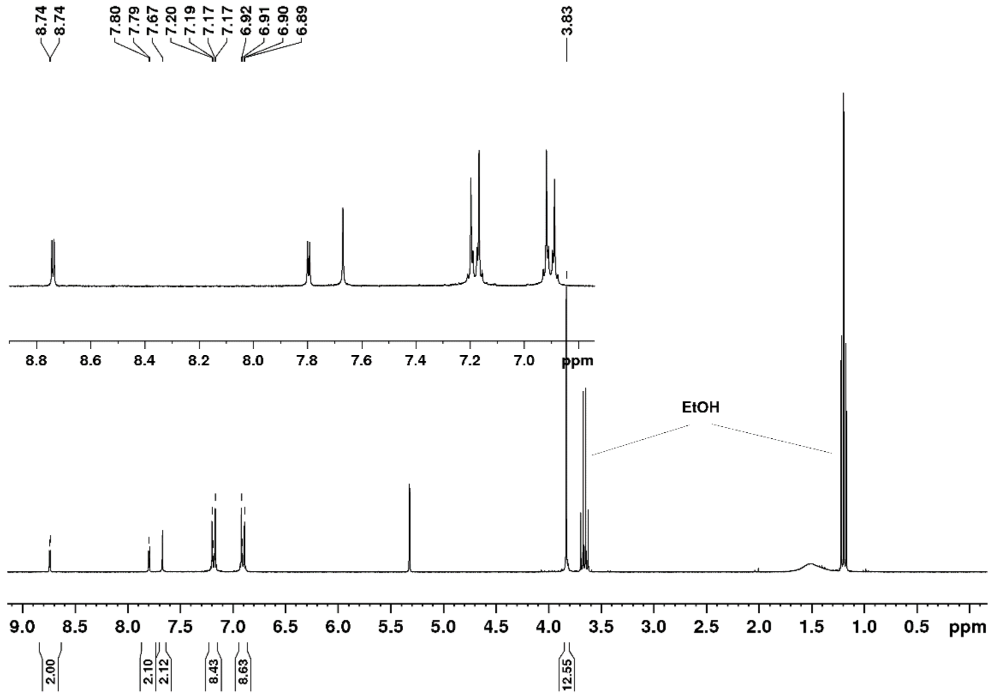

Figure S16. ^{1}H NMR spectrum of compound **7** recorded in CD_2Cl_2 at 300 MHz.

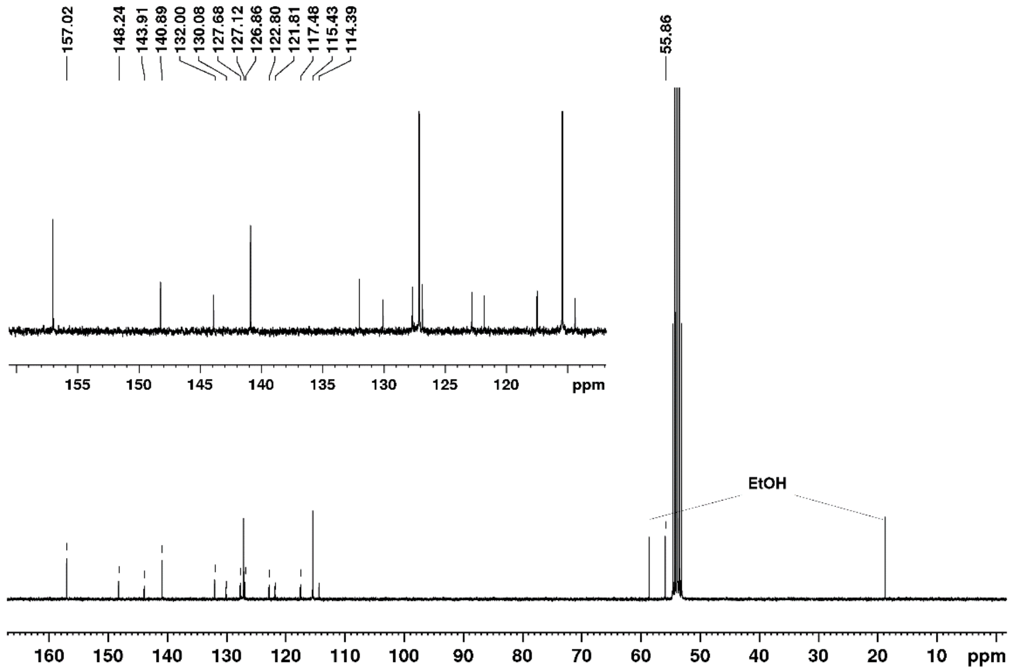

Figure S17. $^{13}C\{^1H\}$ NMR spectrum of compound **7** recorded in CD_2Cl_2 at 75 MHz.

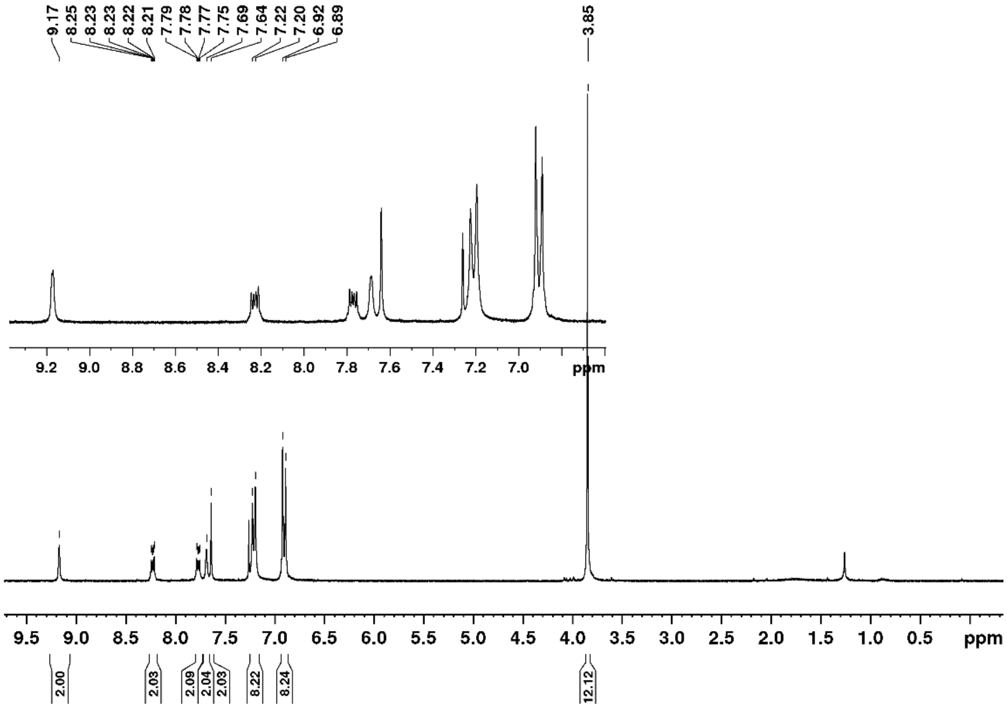

Figure S18. ^1H NMR spectrum of compound **8** recorded in $CDCl_3$ at 300 MHz.

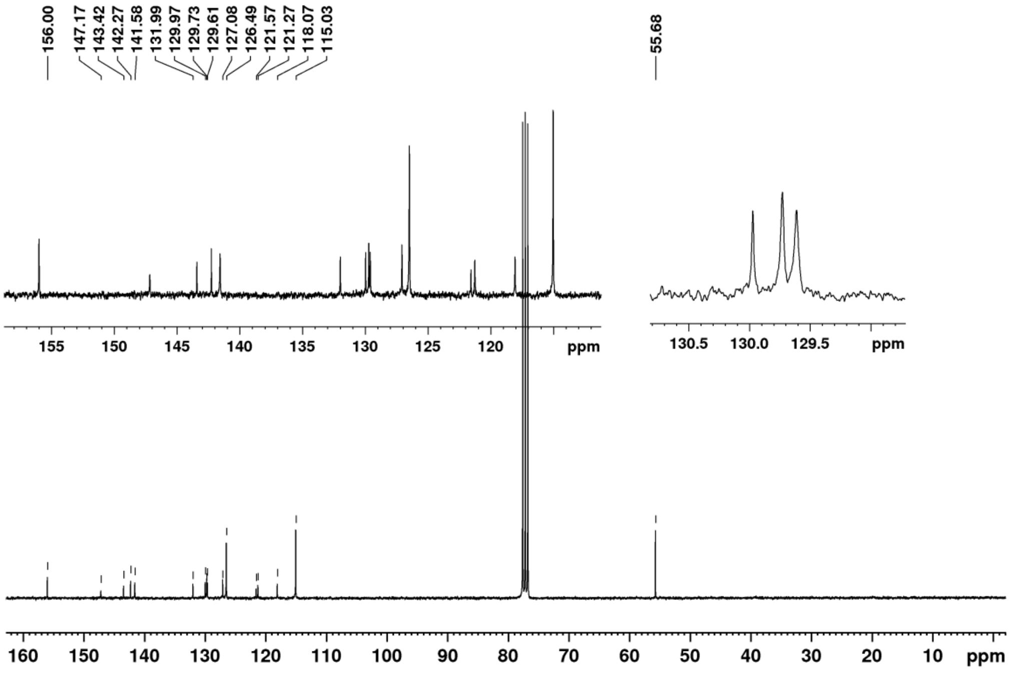

Figure S19. $^{13}C\{^{1}H\}$ NMR spectrum of compound **8** recorded in $CDCl_3$ at 75 MHz.

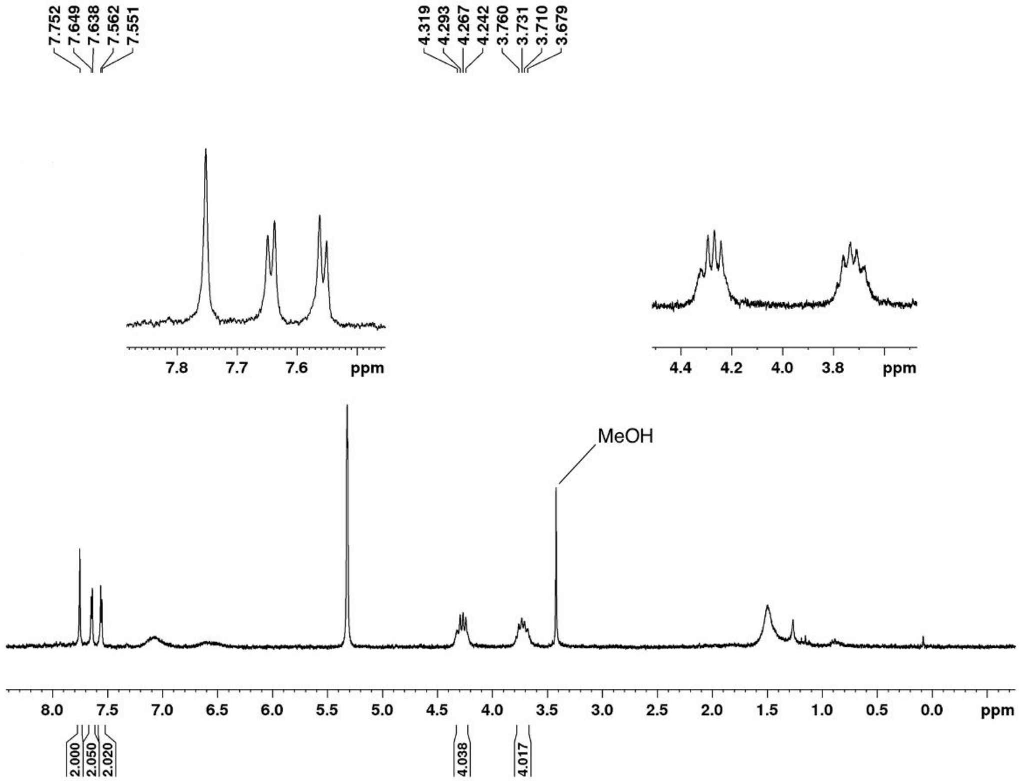

Figure S20. ^{1}H NMR spectrum of compound **4'** recorded in CD_2Cl_2 at 300 MHz.

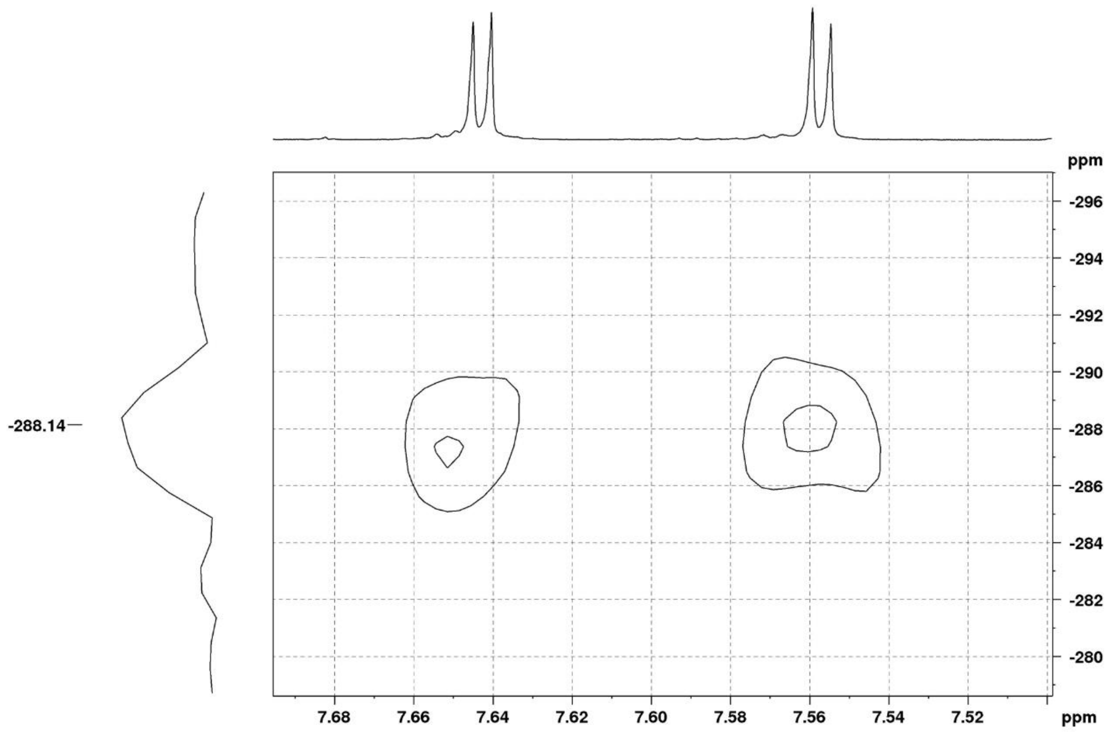

Figure S21. ^{15}N ^{1}H HMBC NMR spectrum of compound **4'** recorded in CD_2Cl_2 at 500 MHz.

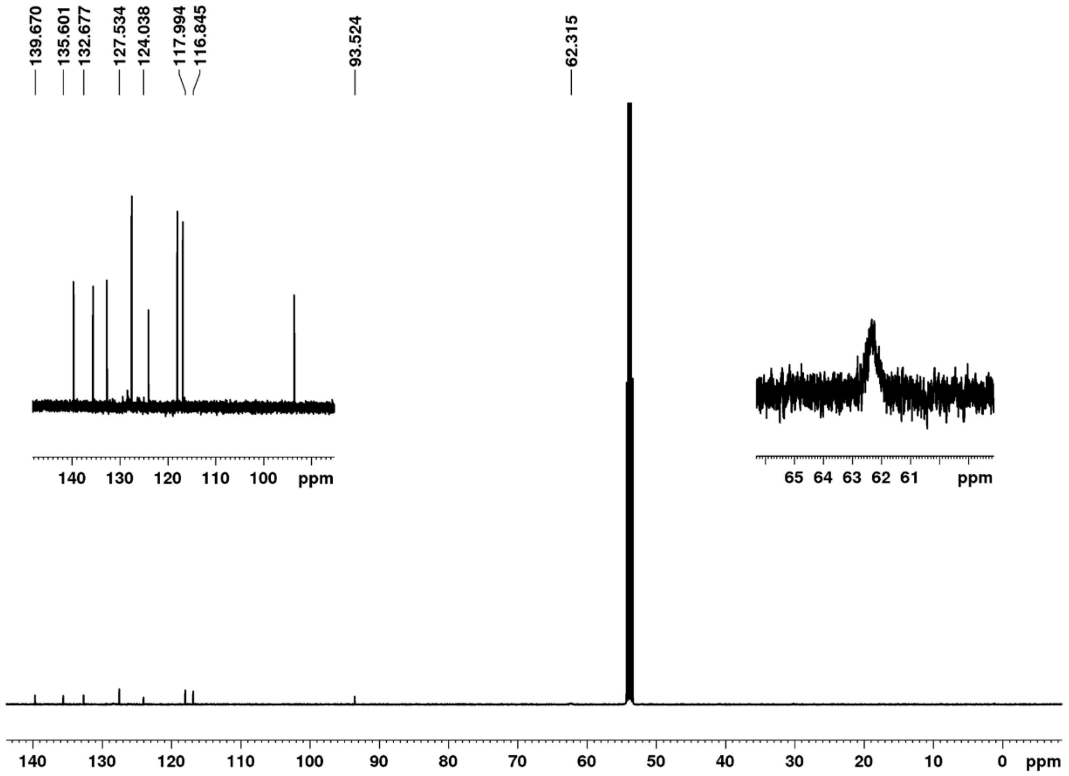

Figure S22. $^{13}C\{^{1}H\}$ NMR spectrum of compound **4'** recorded in CD_2Cl_2 at 75 MHz.

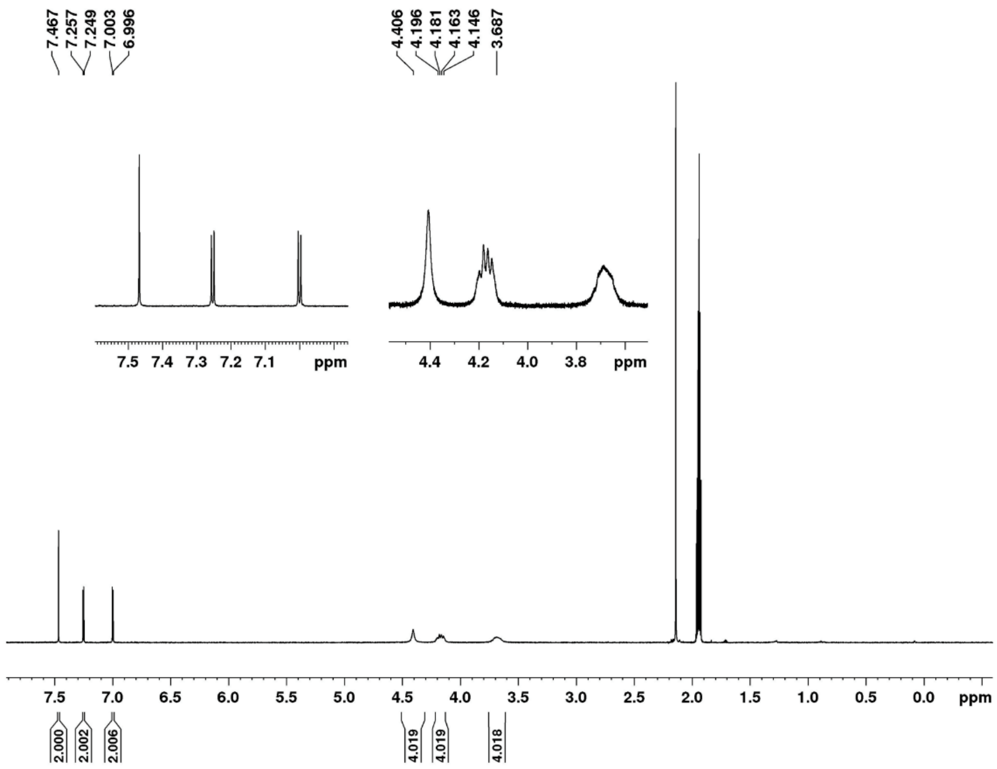

Figure S23. ^{1}H NMR spectrum of compound **5’** recorded in CD_3CN at 300 MHz.

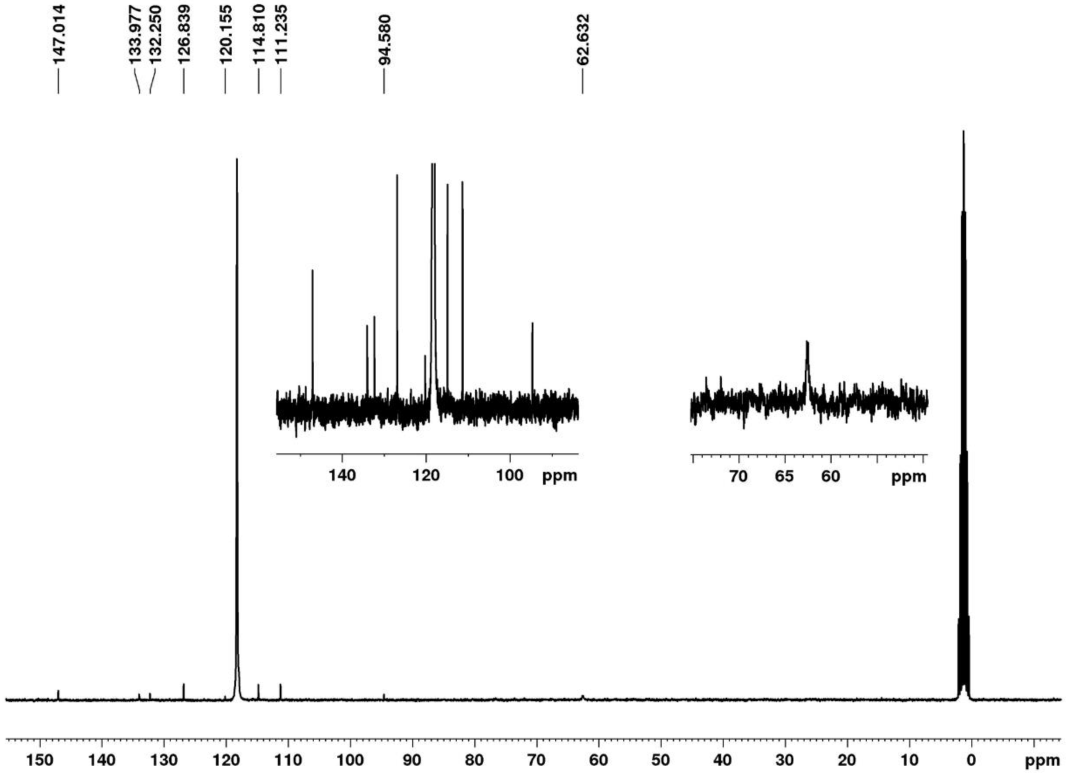

Figure S24. ^{13}C{^{1}H} NMR spectrum of compound **5’** recorded in CD_3CN at 75 MHz.

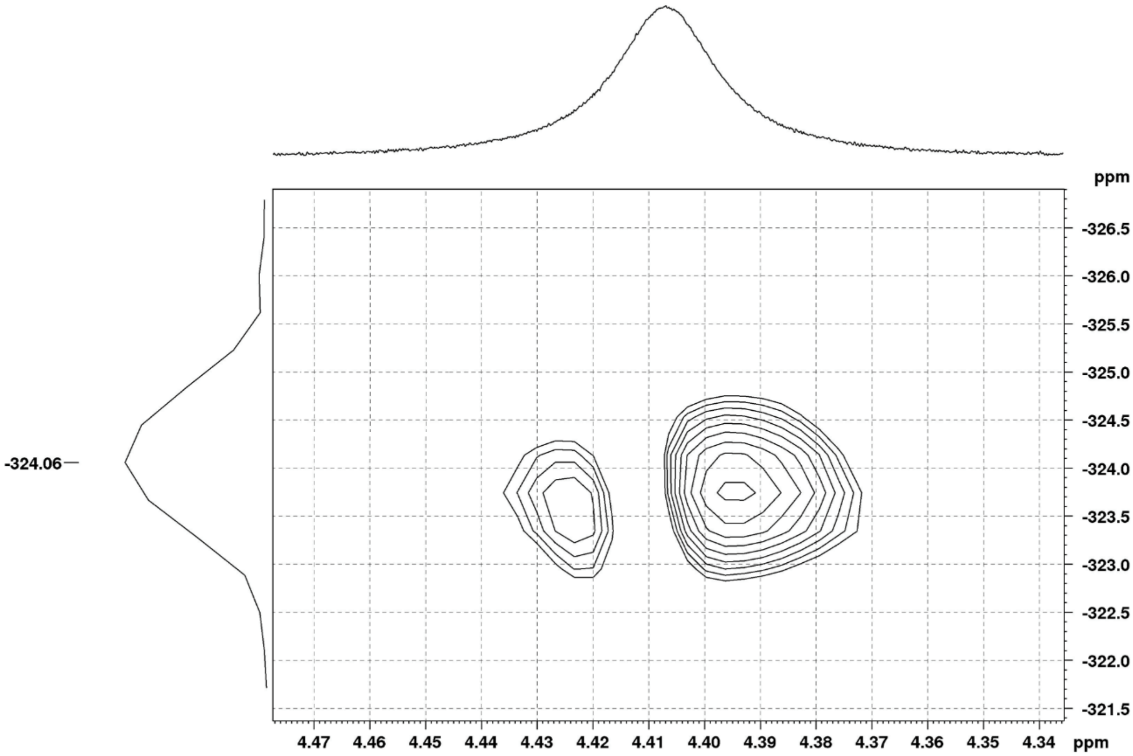

Figure S25. ^{15}N ^{1}H HMBC NMR spectrum of compound **5'** recorded in CD_3CN at 300 MHz.

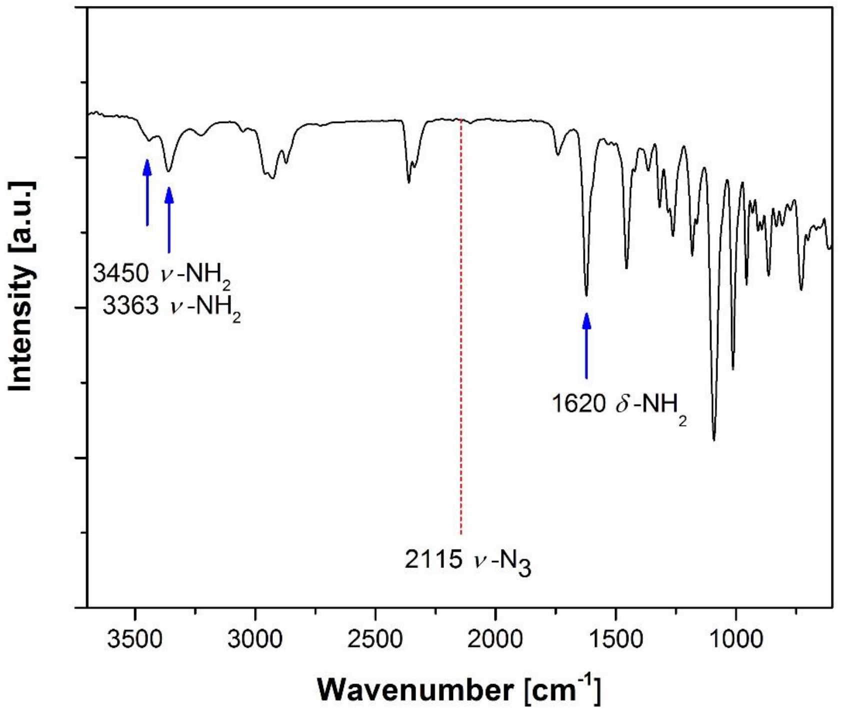

Figure S26. FTIR spectrum of the crude reaction mixture of compound **5'**.

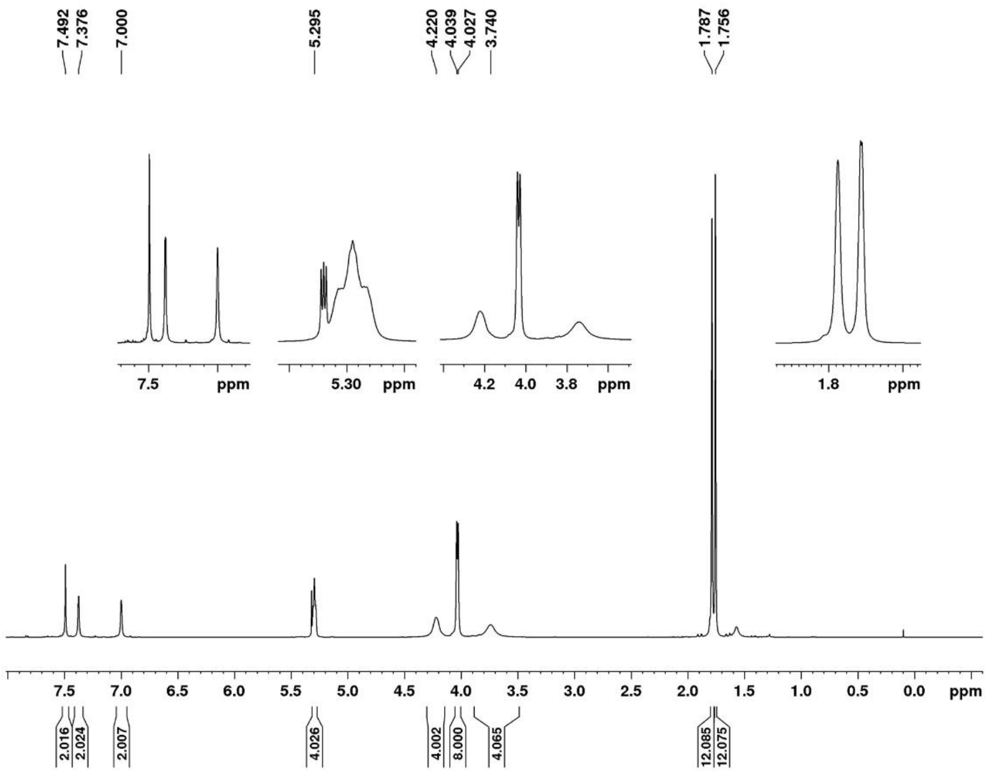

Figure S27. ^{1}H NMR spectrum of compound **6’** recorded in CD_2Cl_2 at 500 MHz.

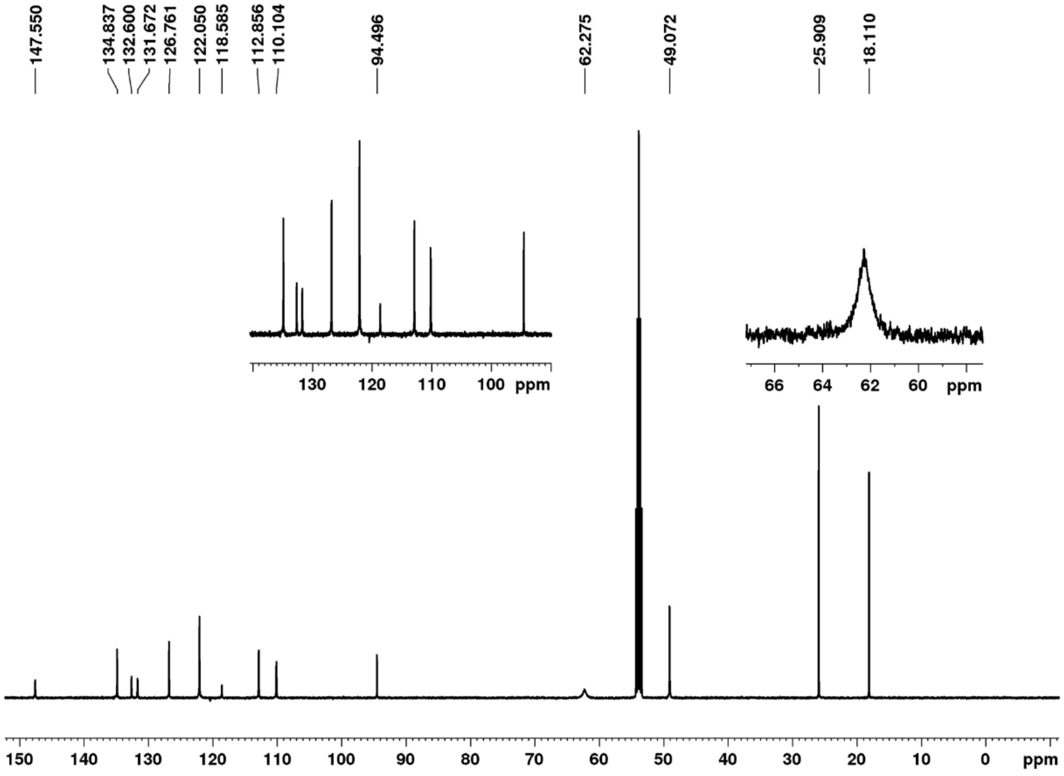

Figure S28. $^{13}C\{^1H\}$ NMR spectrum of compound **6’** recorded in CD_2Cl_2 at 125 MHz.

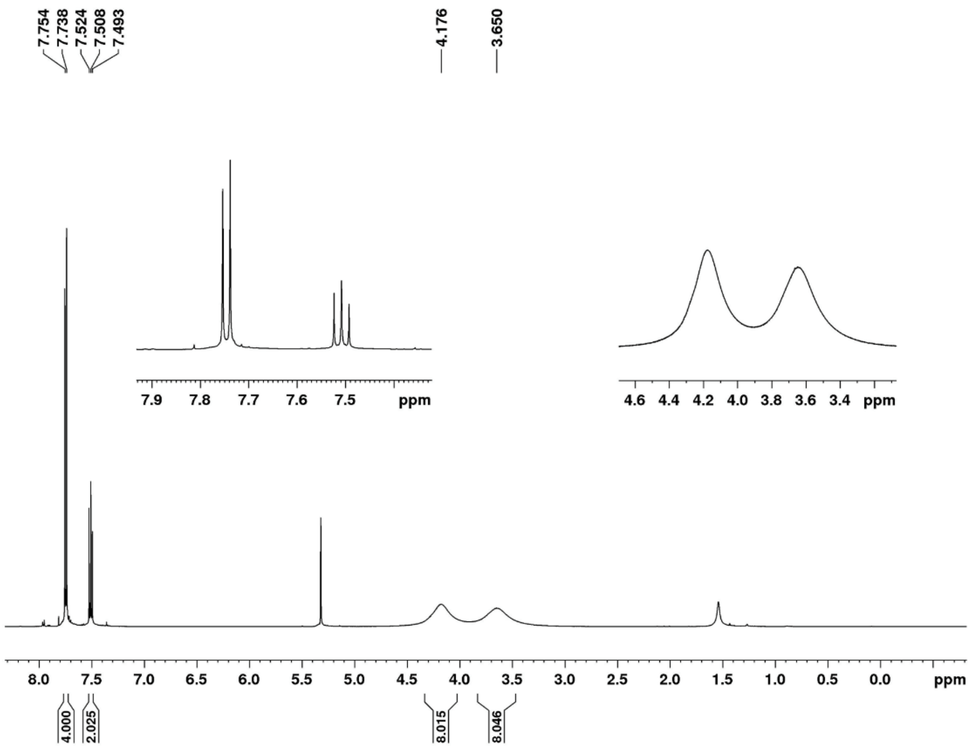

Figure S29. ^{1}H NMR spectrum of compound **10** recorded in CD_2Cl_2 at 500 MHz.

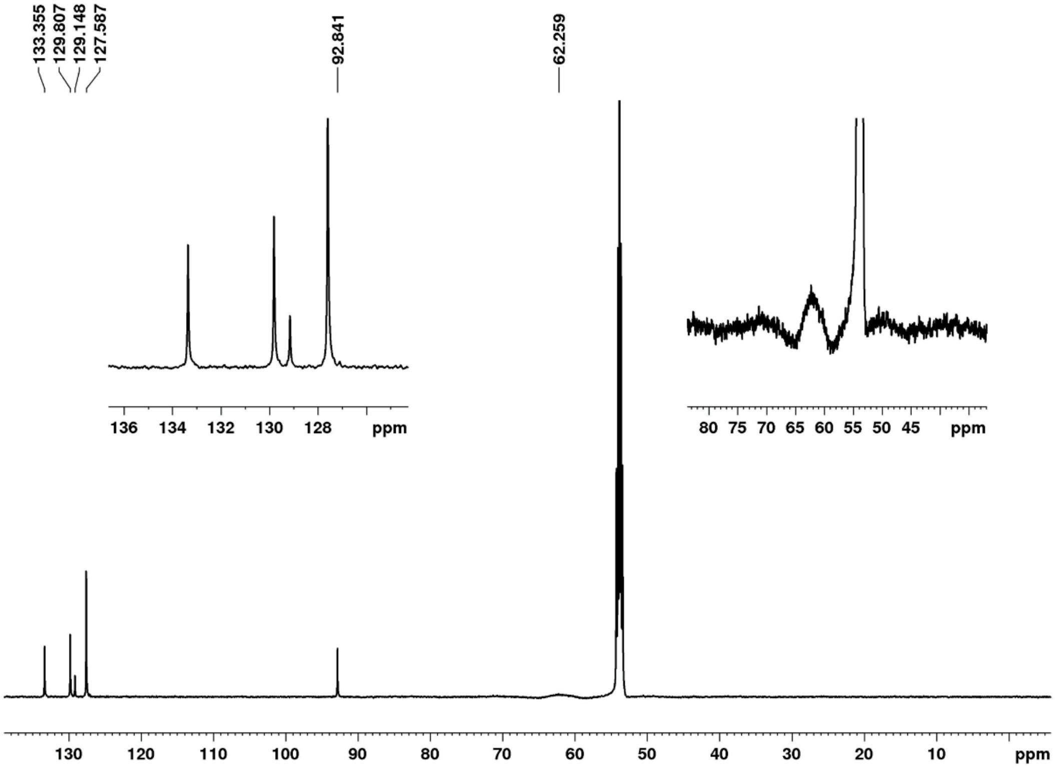

Figure S30. $^{13}C\{^{1}H\}$ NMR spectrum of compound **10** recorded in CD_2Cl_2 at 125 MHz.

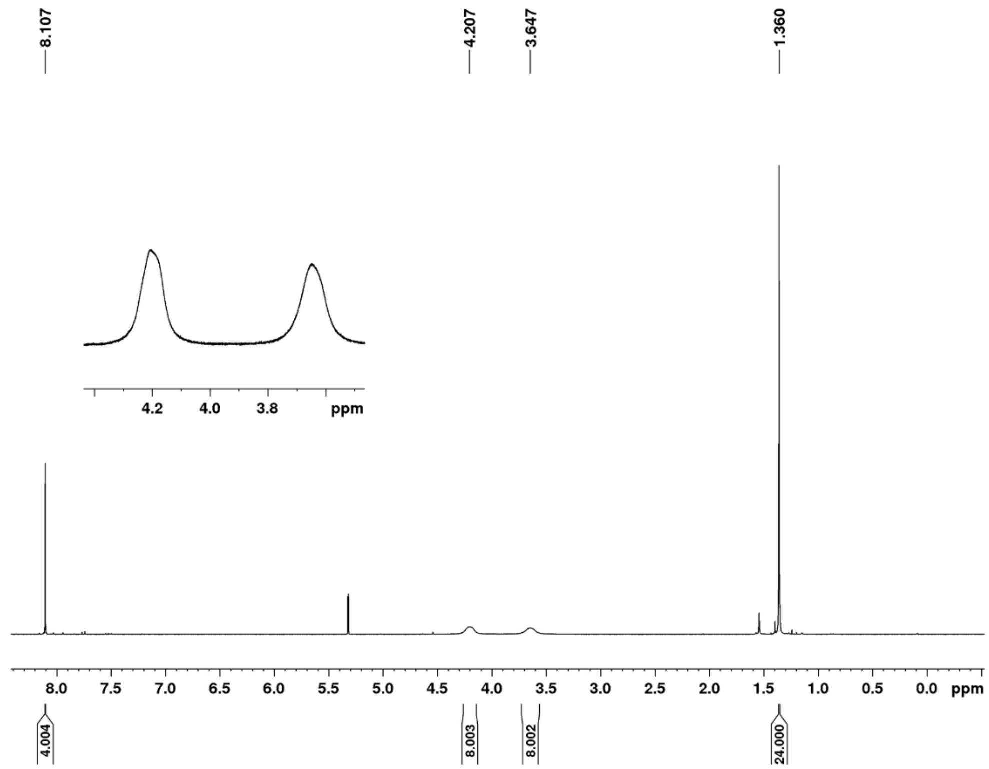

Figure S31. ^{1}H NMR spectrum of compound **11** recorded in CD_2Cl_2 at 300 MHz.

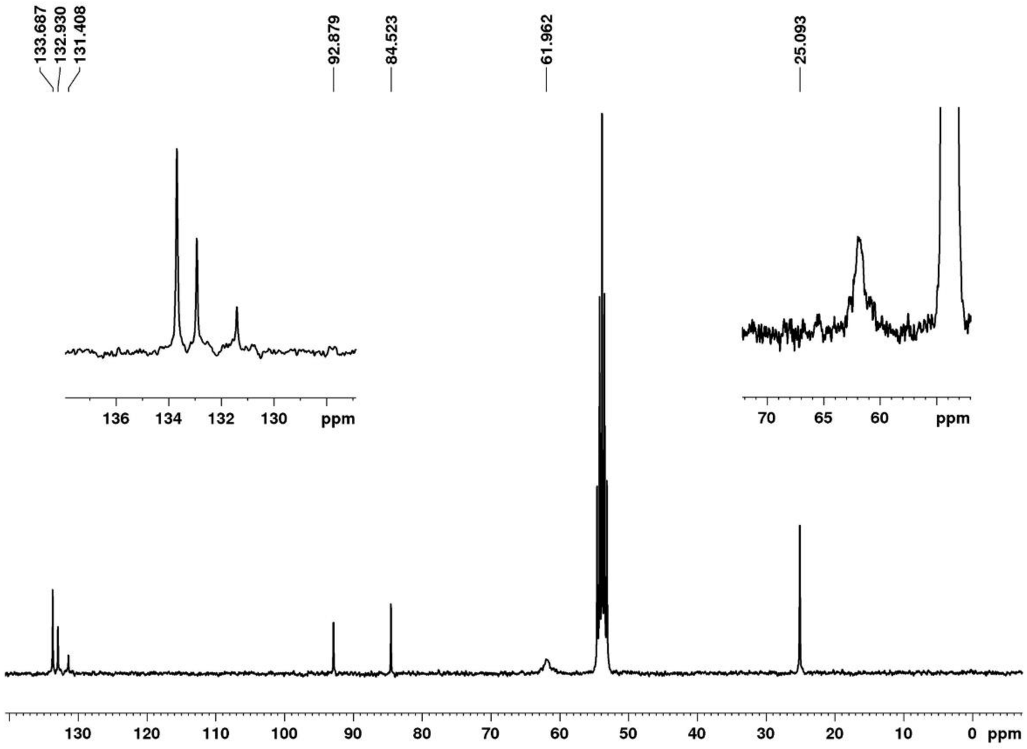

Figure S32. $^{13}C\{^{1}H\}$ NMR spectrum of compound **11** recorded in CD_2Cl_2 at 75 MHz.

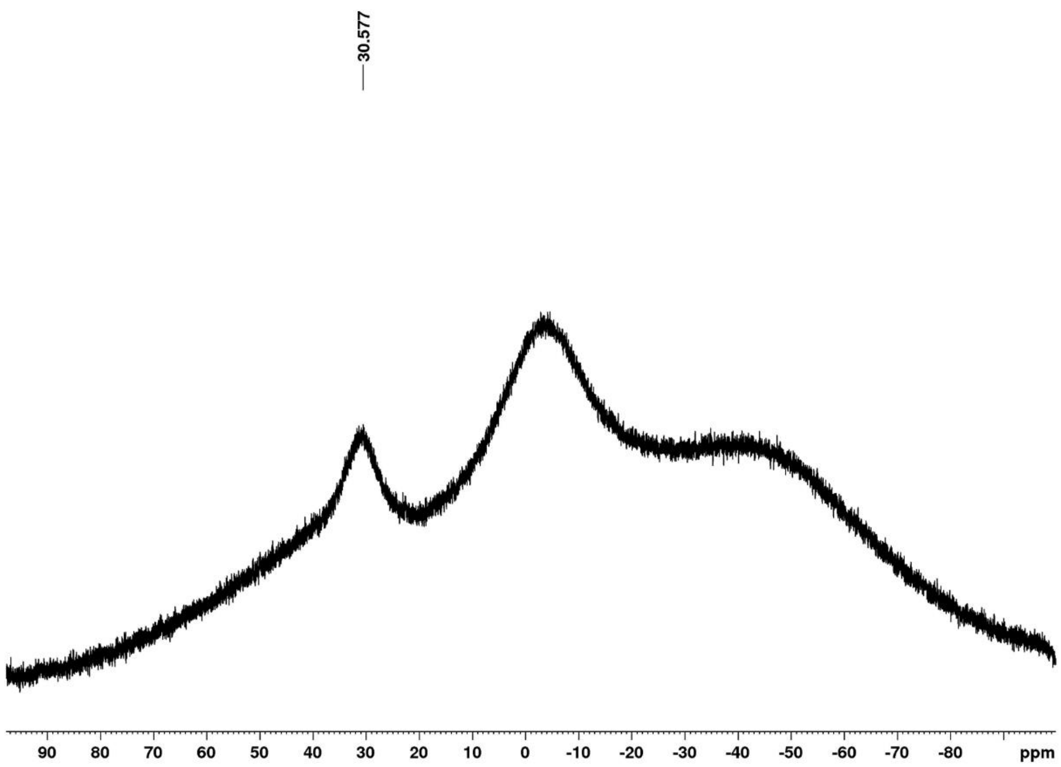

Figure S33. ^{13}B NMR spectrum of compound **11** recorded in CD_2Cl_2 at 96 MHz.

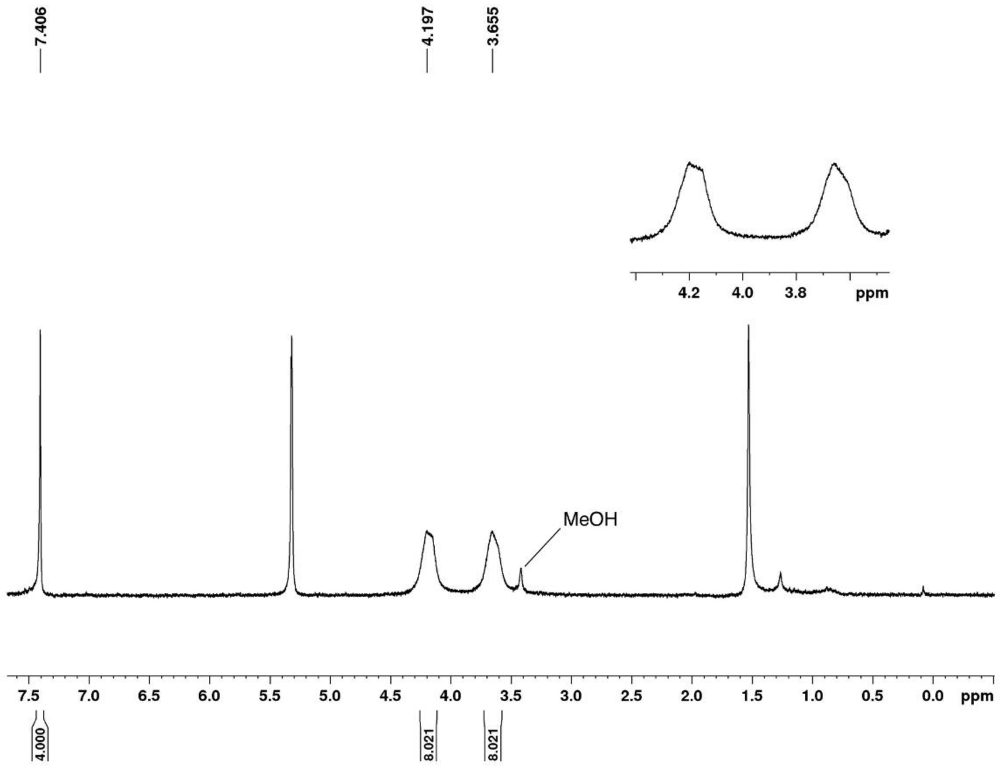

Figure S34. ^{1}H NMR spectrum of compound **12** recorded in CD_2Cl_2 at 300 MHz.

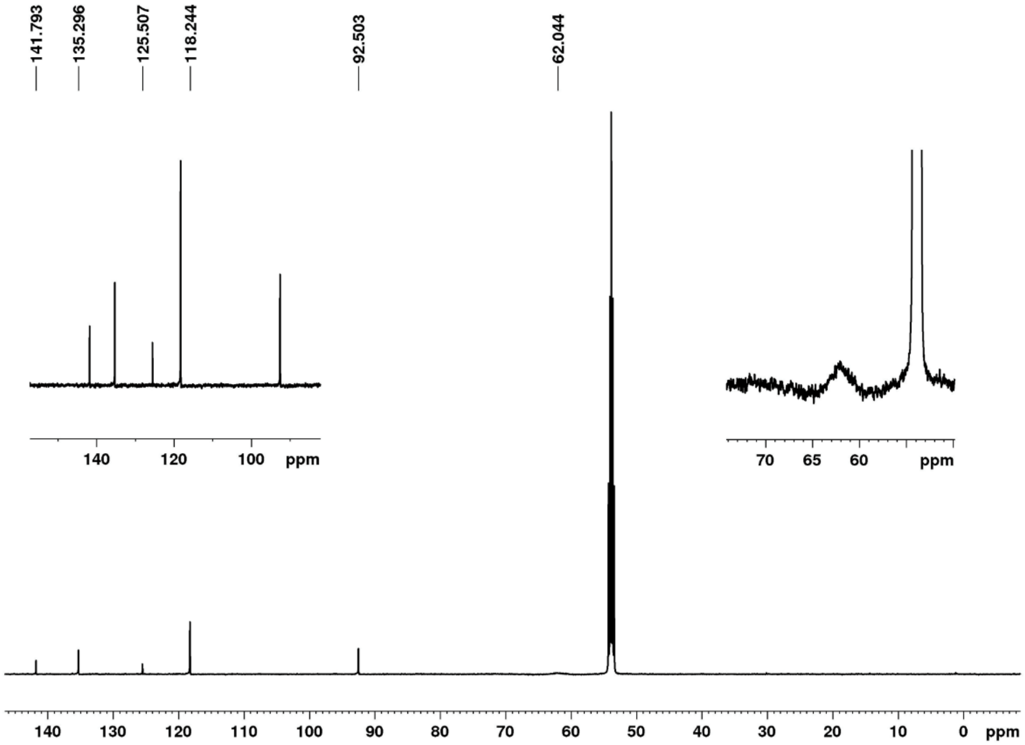

Figure S35. $^{13}C\{^{1}H\}$ NMR spectrum of compound **12** recorded in CD_2Cl_2 at 75 MHz.

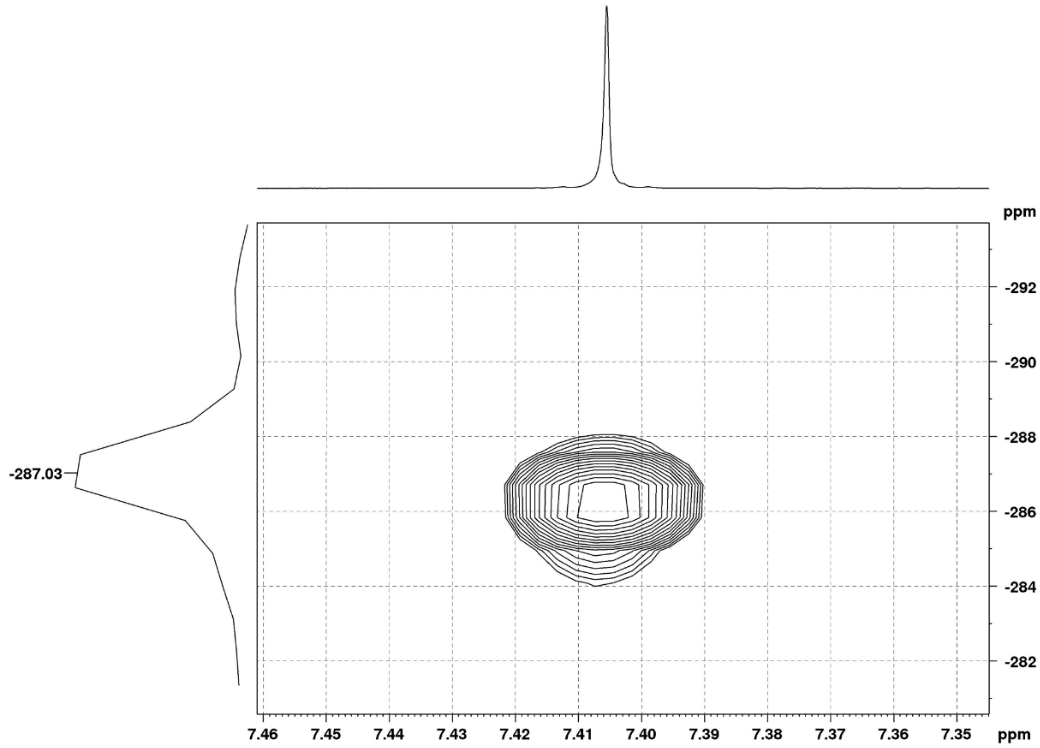

Figure S36. ^{15}N ^{1}H HMBC NMR spectrum of compound **12** recorded in CD_2Cl_2 at 300 MHz.

Single-crystal X-ray diffraction

Crystals suitable for single-crystal X-ray diffraction were selected, coated in perfluoropolyether oil, and mounted on MiTeGen sample holders. Diffraction data were collected on a Bruker X8 Apex II 4-circle diffractometer with a CCD area detector using Mo-Kα radiation monochromated by graphite (**2**, **6'**, **11**, **12**) or multi-layer focusing mirrors (**5'**, **10**). Diffraction data of **7** were collected on a Bruker D8 Quest 4-circle diffractometer with a CMOS area detector (Photon II) and multi-layer mirror monochromated Mo-Kα radiation. The crystals were cooled using Oxford Cryostreams or Bruker Kryoflex II low-temperature devices. Data were collected at 100 K. The images were processed and corrected for Lorentz-polarization effects and absorption as implemented in the Bruker software packages. The structures were solved using the intrinsic phasing method (SHELXT)[1] and Fourier expansion technique. All non-hydrogen atoms were refined in anisotropic approximation, with hydrogen atoms 'riding' in idealized positions, by full-matrix least squares against F^2 of all data, using SHELXL[1] software. In compound **11**, the coordinates of the hydrogen atom of the water molecule, which lies on a two-fold rotation axis, were refined freely; restraints were applied to the O–H and H–H distances. In compound **5'** the coordinates of the hydrogen atoms bonded to nitrogen were refined freely. For methoxyphenyl groups as well as the tetrahydrofuran solvent molecule are strongly disordered in compound **7**. Hence, several restraints had to be applied during the refinement. Diamond[2] software was used for graphical representation. Hirshfeld surfaces were calculated and analyzed using the CrystalExplorer[3] program. Other structural information was extracted using Mercury[4] and OLEX2[5] software. Crystal data and experimental details are listed in Table S1; full structural information has been deposited with the Cambridge Structural Database. CCDC-1917153 (**2**), 1917154 (**5'**), 1917155 (**6'**), 1917156 (**7**), 1917157 (**10**), 1917158 (**11**), and 1917159 (**12**).

Table S1: Single-crystal X-ray diffraction data and structure refinement of **2**, **5'**, **6'**, **7**, **10**, **11**, and **12**.

**Compound**	**2**	**5'**	**6'**
CCDC	1917153	1917154	1917155
Formula	$C_{20}H_{16}O_4$	$C_{20}H_{18}N_2O_4$	$C_{40}H_{50}N_2O_4$
$\rho_{x.}$/ g cm^{-3}	1.488	1.446	1.233
F(000)	336	736	672
Crystal size/mm^3	0.36×0.36×0.37	0.09×0.27×0.47	0.24×0.39×0.47
Crystal color, habit	colorless needle	yellow plate	yellow plate
μ/mm^{-1}	0.104	0.102	0.079
M_r/g·mol^{-1}	320.33	350.36	622.82
T/K	100(2)	100(2)	100(2)
λ/Å, radiation	0.71073, Mo-Kα	0.71073, Mo-Kα	0.71073, Mo-Kα
Crystal system	orthorhombic	monoclinic	triclinic
Space group	$P2_12_12$	Cc	$P\bar{1}$
a/Å	9.6450(8)	9.522(3)	8.987(4)
b/Å	7.7838(6)	15.842(4)	9.778(4)
c/Å	9.5226(8)	10.804(3)	19.688(8)
α/°	90	90	76.792(16)
β/°	90	99.100(17)	88.80(3)
γ/°	90	90	84.95(2)
V/Å^3	714.91(10)	1609.4(8)	1677.8(13)
Z	2	4	2
θ_{max}/°	29.977	28.989	25.086
Reflections collected	8187	24616	34999
Unique refls.	2030	3662	5964
Parameters / restraints	109 / 0	251 / 2	443 / 11
GooF on F^2	1.051	1.025	1.049
R_1 [I>2σ(I)]	0.0356	0.0405	0.0525
wR_2 (all data)	0.0907	0.0990	0.1442
Max./min. residual electron density/e·Å^{-3}	0.341/-0.236	0.378/-0.280	0.296/-0.308

Table S1 continued.

Compound	7	10	11
CCDC	1917156	1917157	1917158
Formula	$C_{48}H_{34}N_6O_4{\cdot}C_4H_8O$	$C_{24}H_{22}O_8$	$C_{36}H_{44}B_2O_{12}{\cdot}0.333(H_2O)$
$\rho_{x.}$/ g cm^{-3}	1.323	1.475	1.358
F(000)	1744	1840	1108
Crystal size/mm^3	0.26×0.27×0.39	0.22×0.24×0.48	0.32×0.34×0.40
Crystal color, habit	black block	colorless needle	colorless block
μ/mm^{-1}	0.087	0.111	0.101
M_r/g·mol^{-1}	830.91	438.41	696.33
T/K	100(2)	100(2)	100(2)
λ/Å, radiation	0.71073, Mo-Kα	0.71073, Mo-Kα	0.71073, Mo-Kα
Crystal system	monoclinic	orthorhombic	trigonal
Space group	$P2_1/n$	*Pbca*	$P3_121$.
a/Å	12.476(4)	18.248(9)	10.091(4)
b/Å	17.608(7)	11.425(6)	10.091(4)
c/Å	19.153(8)	18.943(10)	28.958(13)
α/°	90	90	90
β/°	97.469(8)	90	90
γ/°	90	90	120
V/Å^3	4172(3)	3949(4)	2554(2)
Z	4	8	3
θ_{max}/°	26.444	26.332	27.469
Reflections collected	42125	32701	47551
Unique refls.	8553	4025	3901
Parameters / restraints	676 / 530	289 / 0	238 / 3
GooF on F^2	1.043	1.011	1.097
R_1 [I>2σ(I)]	0.0727	0.0561	0.0435
wR_2 (all data)	0.2059	0.1434	0.1157
Max./min. residual electron density/e·Å^{-3}	0.395/-0.340	0.327/-0.332	0.599/-0.193

Table S1 continued.

Compound	12
CCDC	1917159
Formula	$C_{24}H_{20}N_6O_8$
$\rho_{x.}$/ g cm^{-3}	1.565
F(000)	2160
Crystal size/mm^3	0.16×0.37×0.76
Crystal color, habit	yellow plate
μ/mm^{-1}	0.121
M_r/g·mol^{-1}	520.46
T/K	100(2)
λ/Å, radiation	0.71073, Mo-Kα
Crystal system	orthorhombic
Space group	*Pbca*
a/Å	11.599(2)
b/Å	19.104(15)
c/Å	19.942(18)
α/°	90
β/°	90
γ/°	90
V/Å^3	4419(5)
Z	8
θ_{max}/°	27.995
Reflections collected	21413
Unique refls.	5185
Parameters / restraints	343 / 0
GooF on F^2	1.040
R_1 [I>2σ(I)]	0.0603
wR_2 (all data)	0.1831
Max./min. residual electron density/e·Å^{-3}	0.523/-0.775

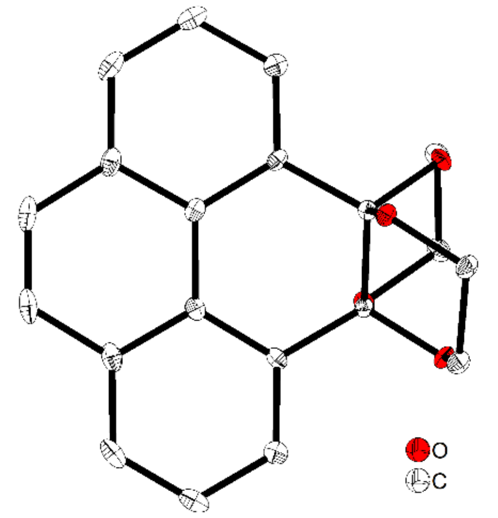

Figure S37. Solid state molecular structure of compound **2** from single-crystal X-ray diffraction at 100 K. Atomic displacement ellipsoids are drawn at the 50% probability level, and hydrogen atoms are omitted for clarity. The molecule has 2-fold rotational symmetry.

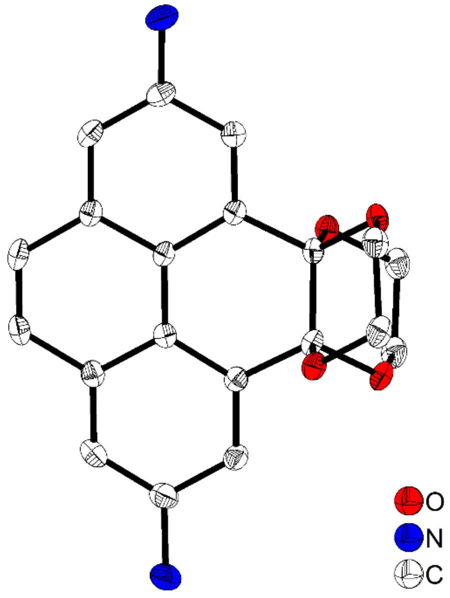

Figure S38. Solid state molecular structure of compound **5’** from single-crystal X-ray diffraction at 100 K. Atomic displacement ellipsoids are drawn at the 50% probability level, and hydrogen atoms are omitted for clarity.

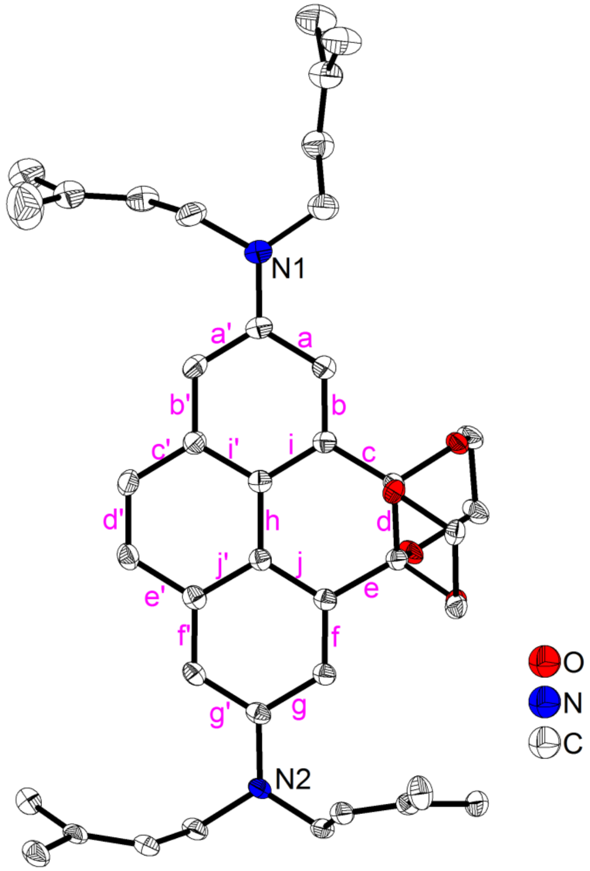

Figure S39. Solid state molecular structure of compound **6’** from single-crystal X-ray diffraction at 100 K. Atomic displacement ellipsoids are drawn at the 50% probability level, and hydrogen atoms are omitted for clarity. The two alkyl chains bonded to the N1 nitrogen atom are disordered and only the major parts with an occupancy of 89% are shown here.

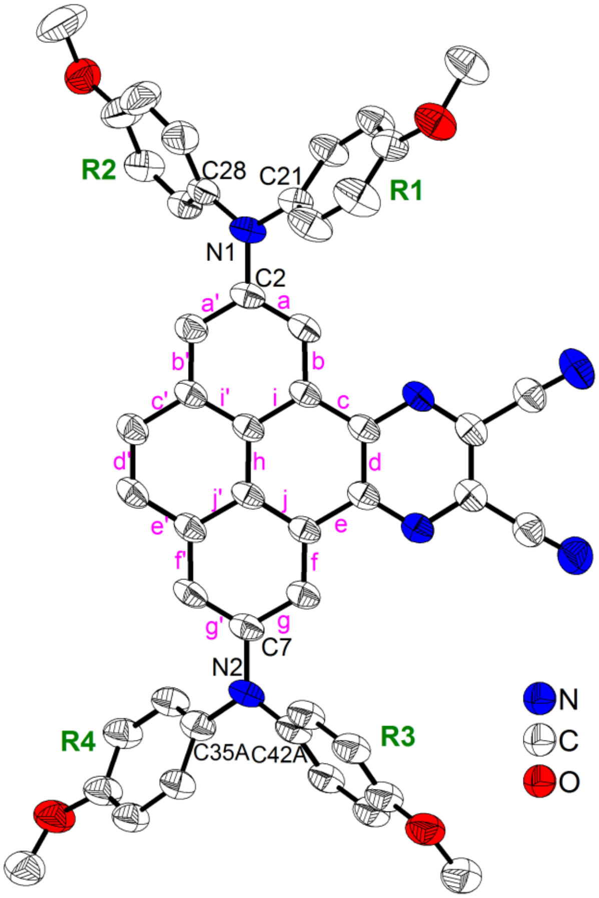

Figure S40. Solid state molecular structure of compound **7** from single-crystal X-ray diffraction at 100 K. Atomic displacement ellipsoids are drawn at the 50% probability level. Hydrogen atoms and the disordered tetrahydrofuran solvent molecule are omitted for clarity. The methoxyphenyl groups are strongly disordered and only the parts with the highest occupancies are shown. Terminal aryl rings bonded to nitrogen are labelled R1 and R2 if bonded to N1 and R3 and R4 if bonded to N2, respectively.

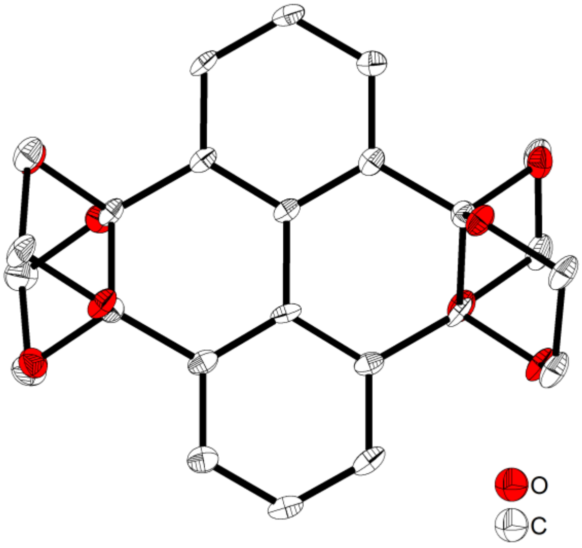

Figure S41. Solid state molecular structure of compound **10** from single-crystal X-ray diffraction at 100 K. Atomic displacement ellipsoids are drawn at the 50% probability level, and hydrogen atoms are omitted for clarity.

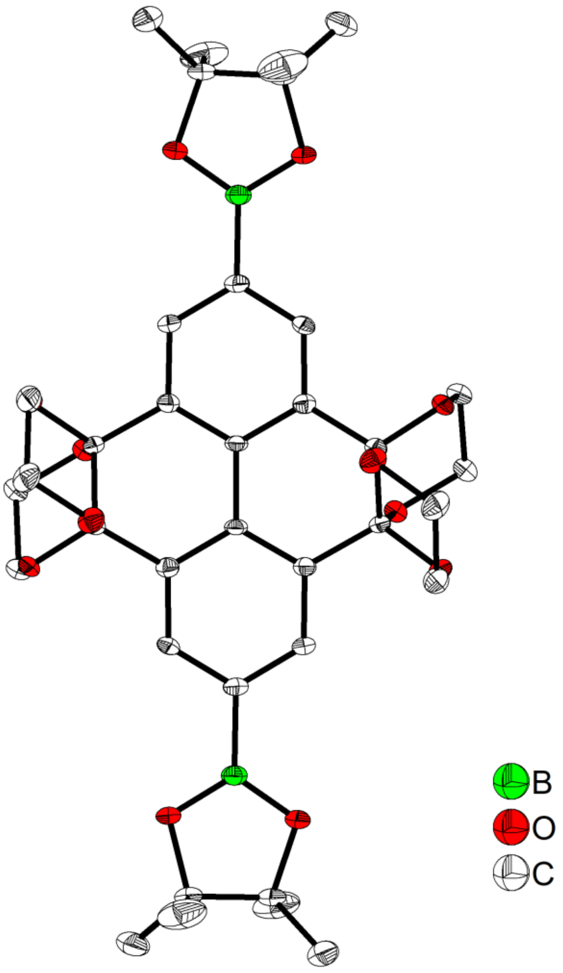

Figure S42. Solid state molecular structure of compound **11** from single-crystal X-ray diffraction at 100 K. Atomic displacement ellipsoids are drawn at the 50% probability level. Hydrogen atoms and the water molecule are omitted for clarity. The molecule has 2-fold rotational symmetry.

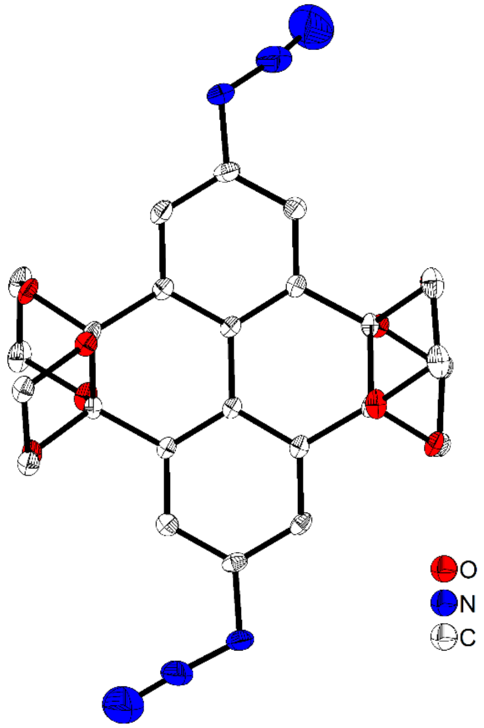

Figure S43. Solid state molecular structure of compound **12** from single-crystal X-ray diffraction at 100 K. Atomic displacement ellipsoids are drawn at the 50% probability level, and hydrogen atoms are omitted for clarity.

Table S2. Selected bond lengths (Å) and angles (°) of compounds **2**, **5'**, **6'**, and **7**. Bonds of the pyrene moiety and of aryl rings are labelled according to Figures S39 and S40.

	**2**	**5'**	**6'** [a]	**7** [b]
a, a'	1.400(2), 1.373(3)	1.413(4), 1.380(4)	1.420(3), 1.395(3)	1.396(4), 1.392(3)
b, b'	1.379(2), 1.407(3)	1.375(4), 1.403(4)	1.377(3), 1.406(3)	1.400(3), 1.398(3)
c, c'	1.515(2), 1.436(3)	1.521(4), 1.441(4)	1.522(3), 1.440(3)	1.451(3), 1.444(3)
d, d'	1.552(3), 1.346(4)	1.549(4), 1.341(4)	1.555(3), 1.363(3)	1.426(3), 1.355(3)
e, e'	= c, c'	1.518(3), 1.445(4)	1.517(3), 1.436(3)	1.453(3), 1.431(4)
f, f'	= b, b'	1.374(3), 1.404(3)	1.382(3), 1.407(3)	1.392(3), 1.406(3)
g, g'	= a, a'	1.413(4), 1.385(4)	1.415(3), 1.393(3)	1.397(3), 1.391(4)
h	1.447(3)	1.440(3)	1.442(3)	1.435(3)
i, I'	1.407(2), 1.415(2)	1.409(3), 1.414(3)	1.411(3), 1.411(3)	1.418(3), 1.417(3)
j, j'	= I, I'	1.402(3), 1.418(3)	1.414(3), 1.409(3)	1.418(3), 1.417(3)
N1–C_{pyrene}		1.398(4)	1.399(3)	1.406(3)
N1–C (R1)			1.460(4)	1.427(3)
N1–C (R2)			1.477(3)	1.422(3)
N2–C_{pyrene}		1.387(4)	1.386(3)	1.415(3)
N2–C (R3)			1.460(3)	1.429(3)
N2–C (R4)			1.457(3)	1.435(2)
∠ $N1C_3$-pyrene			13.23(17)	37.45(10)
∠ $N1C_3$-R1				46.21(13)
∠ $N1C_3$-R2				38.02(12)
∠ $N2C_3$-pyrene			15.54(13)	32.3(2) / 32.7(4) / 33.2(4)
∠ $N2C_3$-R3				47.5(2) / 38.7(4) / 38.2(4)
∠ $N2C_3$-R4				35.5(3) / 35.2(4) / 39.0(6)
Sum ∠ CN1C			357.7(2)	359.8(2)
Sum ∠ CN2C			359.5(2)	360.0(3) / 360.0(5) / 359.0(6)

[a] The alkyl chains bonded to the N1 nitrogen atom are disordered. Only the distances and angles for the major part (89% occupancy) are given here. [b] The methoxyphenyl groups bonded to the N2 nitrogen atom are strongly disordered. Due to similar occupancies of the three parts, different values for the distances and angles are given.

Table S3. Selected bond lengths (Å) and angles (°) of compounds **10**, **11**, and **12**. Bonds of the pyrene moiety are labelled according to Figures S39 and S40.

	**10**	**11**	**12**
a, a'	1.380(4), 1.392(4)	1.392(4), 1.396(4)	1.397(3), 1.393(3)
b, b'	1.399(4), 1.385(4)	1.381(3), 1.396(3)	1.386(3), 1.389(3)
c, c'	1.520(4), 1.523(4)	1.510(4) 2x	1.518(3), 1.517(3)
d, d'	1.544(4), 1.544(4)	1.548(3) 2x	1.547(3), 1.553(3)
e, e'	1.523(4), 1.523(4)	= c, c'	1.519(3), 1.524(3)
f, f'	1.391(4), 1.394(4)	= b, b'	1.386(3), 1.392(3)
g, g'	1.389(4), 1.375(4)	= a, a'	1.389(4), 1.397(3)
h	1.473(4)	1.474(4)	1.466(3)
i, I'	1.401(4), 1.401(4)	1.400(3), 1.400(4)	1.395(3), 1.406(3)
j, j'	1.400(4), 1.398(4)	= i, i'	1.407(3), 1.397(3)
N1/B1–C_{pyrene}		1.557(4)	1.404(3)
N2–C_{pyrene}			1.407(3)

Table S4. Intermolecular interaction distances (Å) in the crystal of compound **7** at 100 K. Aryl rings are labelled according to Figure S40. Only interactions involving the pyrene-4,5-azaacene-CN moiety are included in this list. Further interactions exist between methoxyphenyl groups and with the THF solvent molecule.

π···π interactions (the dimer)	Centroid-centroid distance	Interplanar separation	Offset shift[a]
pyreneazaacene···pyreneazaacene	3.7246(15)	3.4108(14)	1.496(2)
pyrene···pyrene	4.6133(17)	3.4708(19)	3.039(2)
pyrene···azaacene	3.5183(14)	3.4301(17) / 3.3942(15)	0.783(4) / 0.926(2)
	H···O/N/H	C···C/N/O	Aryl/CN ···Aryl/CN
C11···C18		3.334(4)	pyrene···azaacene
C9···C20		3.382(4)	pyrene···CN
C19···C19		3.362(4)	CN···CN
C33-H33···N4	2.733(2)	3.666(3)	R2···azaacene
C33-H33···H6-C6	2.3618(6)	3.952(3)	R2···pyrene
C8-H8···O1A_1	2.260(7)	3.159(7)	pyrene···THF
C10-H10···H25-C25	2.3747(6)	3.698(4)	pyrene···R1

[a] The offset shift, also called inter-centroid shift, is the distance within a plane of an aryl ring between the centroid of the respective aryl ring and the intersection point with the normal to the plane through the centroid of the other aryl ring.

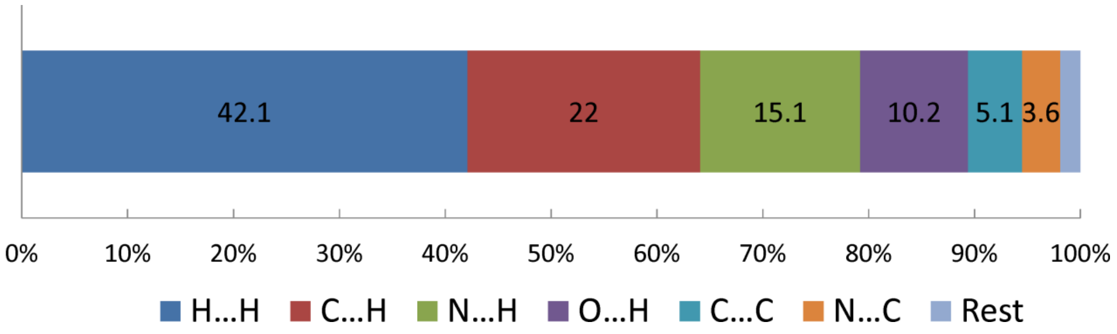

Figure S44. Percentage contributions to the Hirshfeld surface area for the various close intermolecular contacts in **7** at 100 K. Only the major occupied parts of the disordered molecules are considered in this analysis.

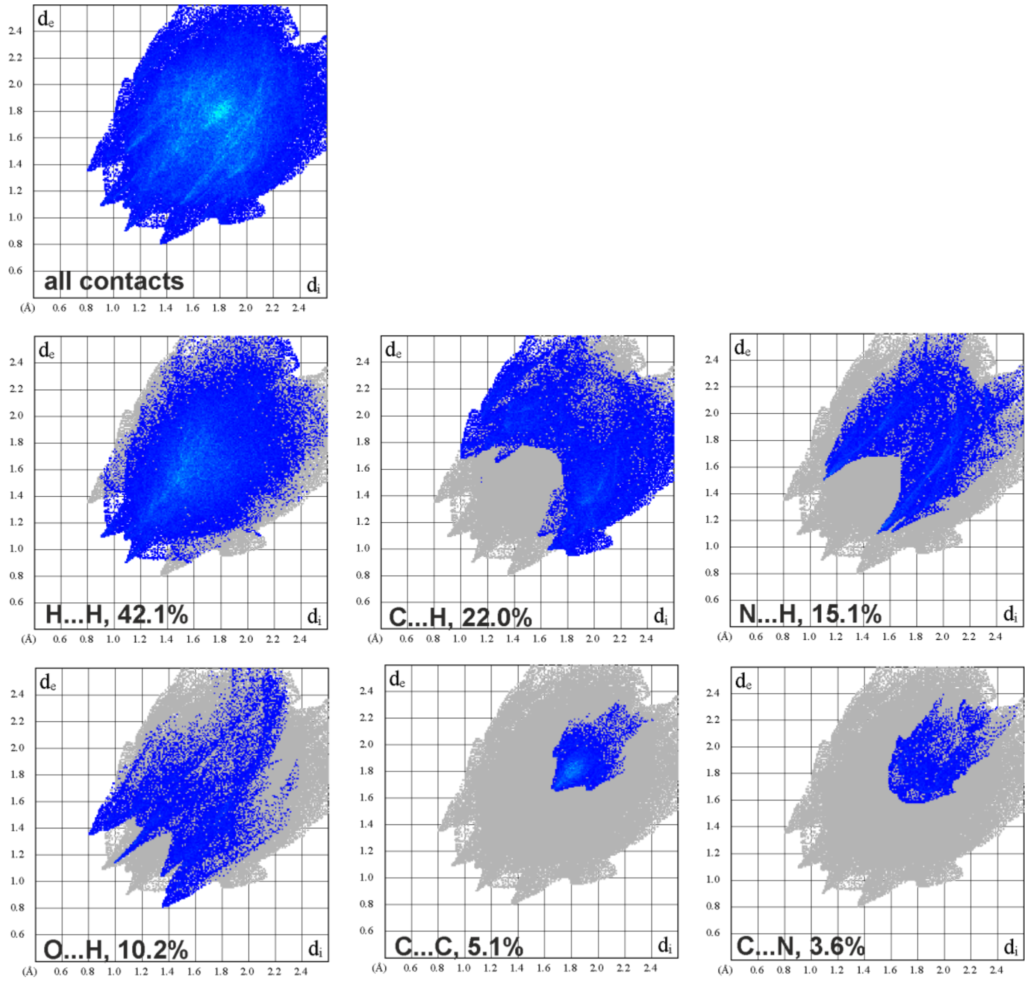

Figure S45. Two-dimensional fingerprint plots of **7** calculated from the Hirshfeld surface at 100 K. The top left figure shows the complete fingerprint plot, while the other plots indicate the contributions of the individual intermolecular interactions within the grey area of all contributions. Only the major occupied parts of the disordered molecules are considered in this analysis.

Depictions of the frontier orbitals of compound 6, 7’ and 15

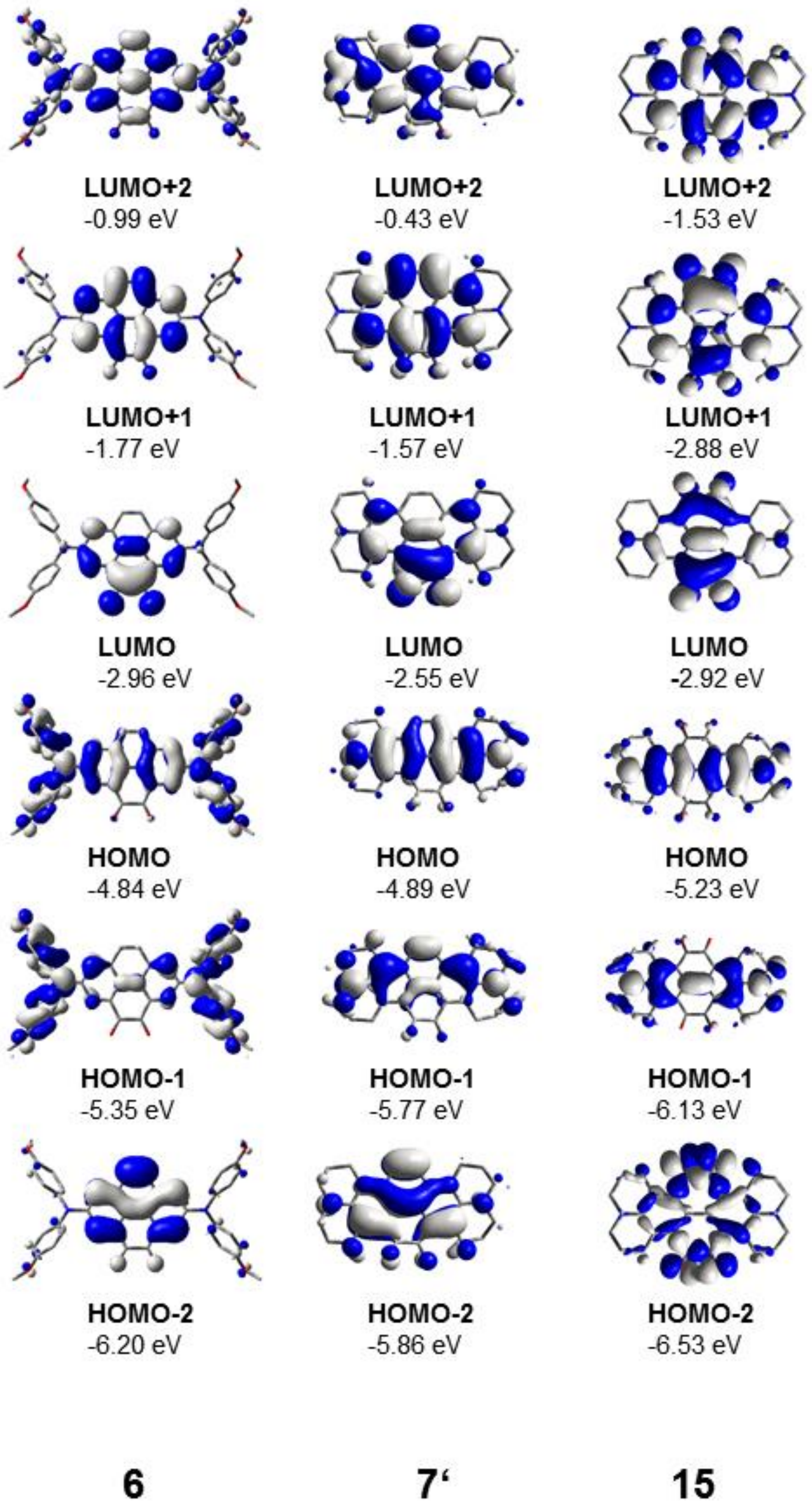

Figure S46. Frontier molecular orbitals of compounds **6**, **7’** and **15** (B3LYP/6-31+G (d)).

TD-DFT Results

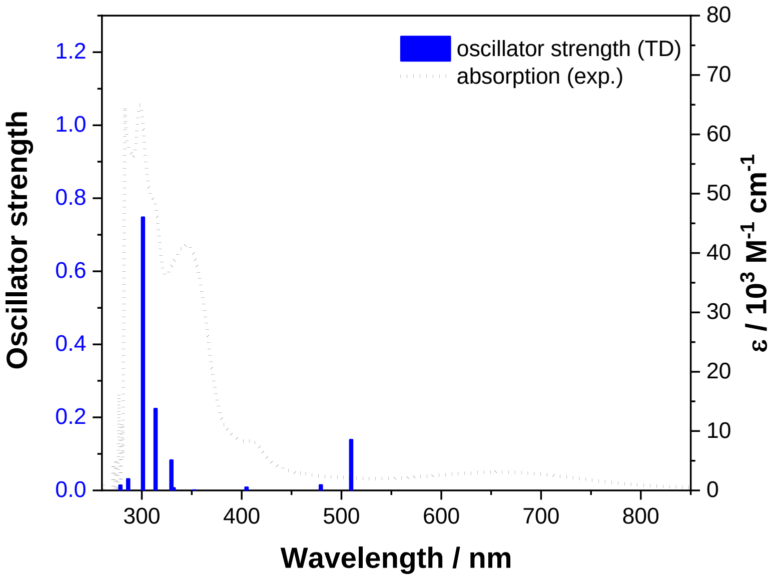

Figure S47. TD-DFT calculated singlet electronic transitions of **6** in the gas phase using CAM-B3LYP/6-31+G (d), and the experimental absorption spectrum, in toluene.

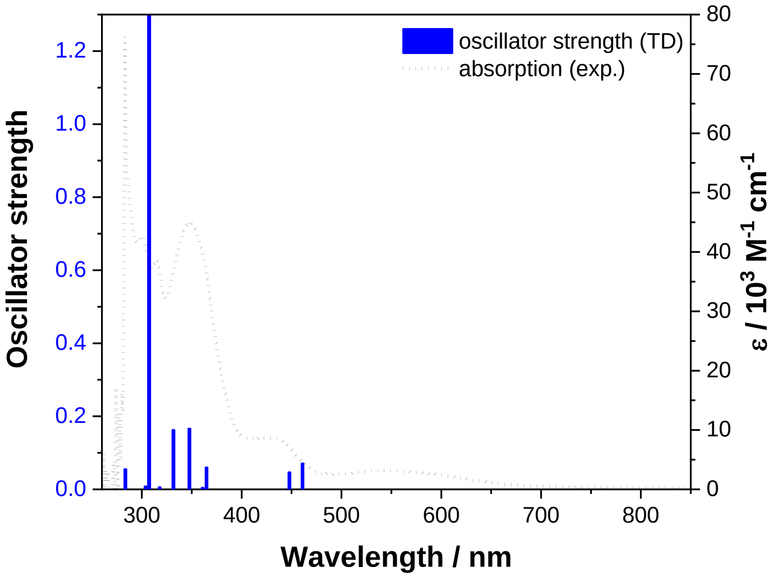

Figure S48. TD-DFT calculated singlet electronic transitions of **7** in the gas phase using CAM-B3LYP/6-31+G (d), and the experimental absorption spectrum, in toluene.

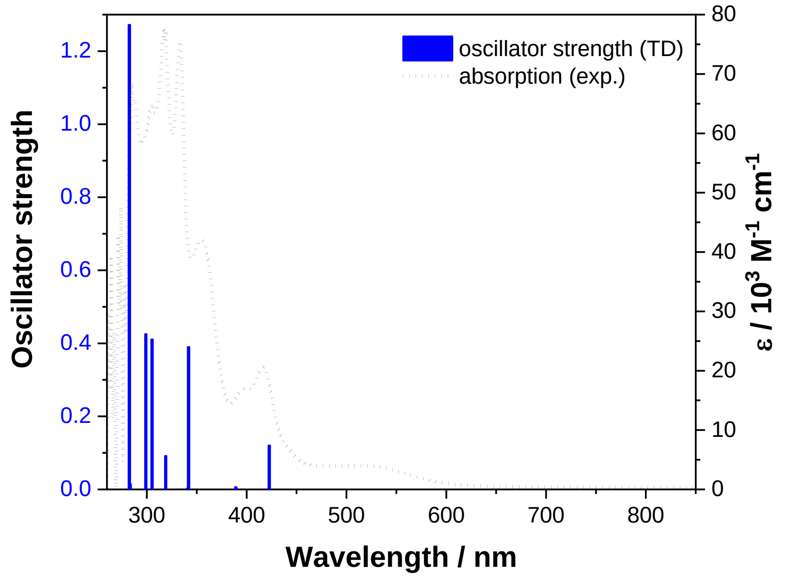

Figure S49. TD-DFT calculated singlet electronic transitions of **7** in the gas phase using CAM-B3LYP/6-31+G (d), and the experimental absorption spectrum, in toluene.

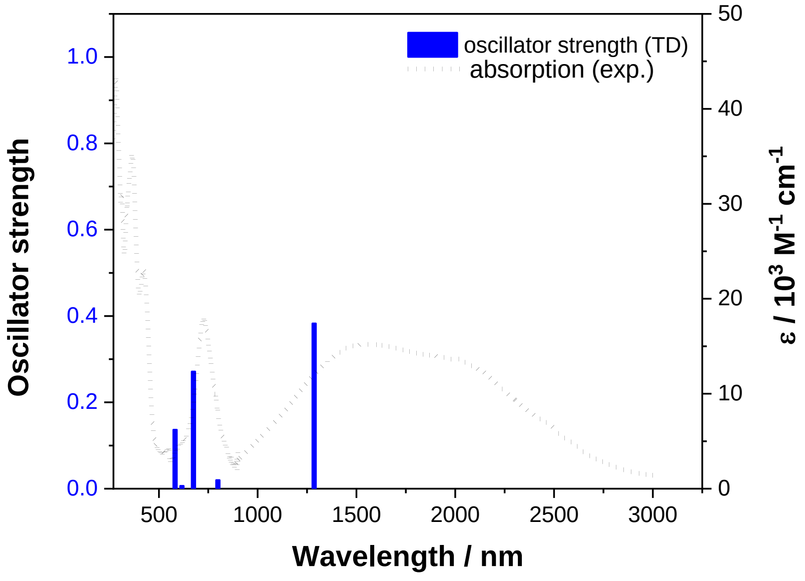

Figure S50. TD-DFT calculated singlet electronic transitions of $\mathbf{7}^{+}$ in dichloromethane using ublyp/svp, and the experimental absorption spectrum, in dichloromethane.

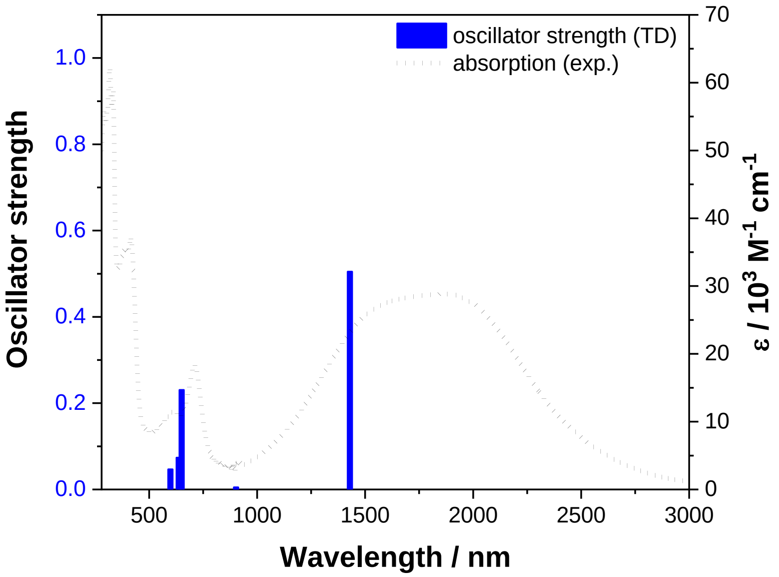

Figure S51. TD-DFT calculated singlet electronic transitions of **8**$^{+}$ in dichloromethane using ublyp/svp, and the experimental absorption spectrum, in dichloromethane.

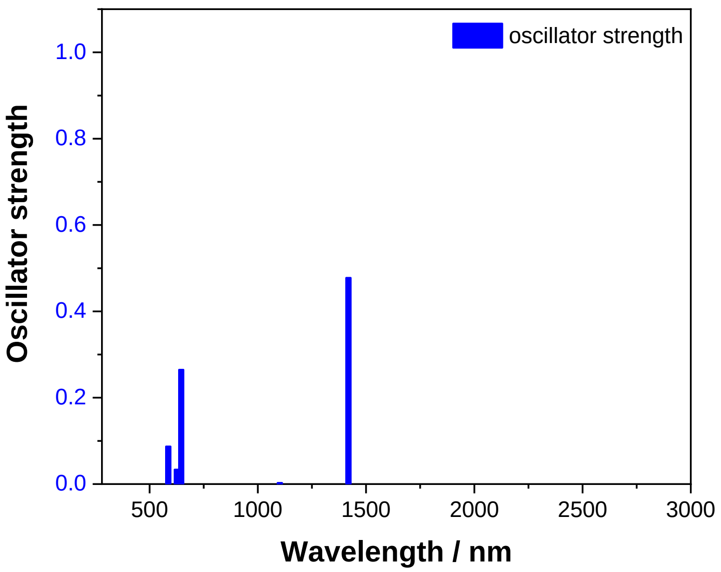

Figure S52. TD-DFT calculated singlet electronic transitions of **III**$^{+}$ in dichloromethane using ublyp/svp.

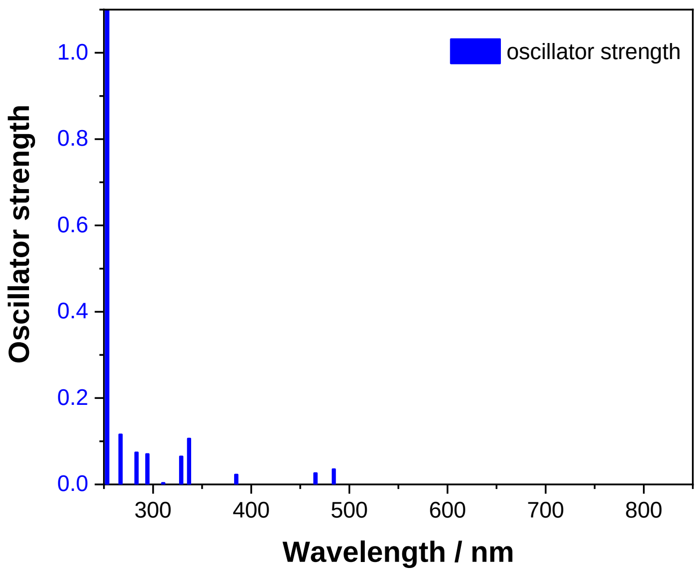

Figure S53. TD-DFT calculated singlet electronic transitions of **6'** in the gas phase using CAM-B3LYP/6-31+G (d).

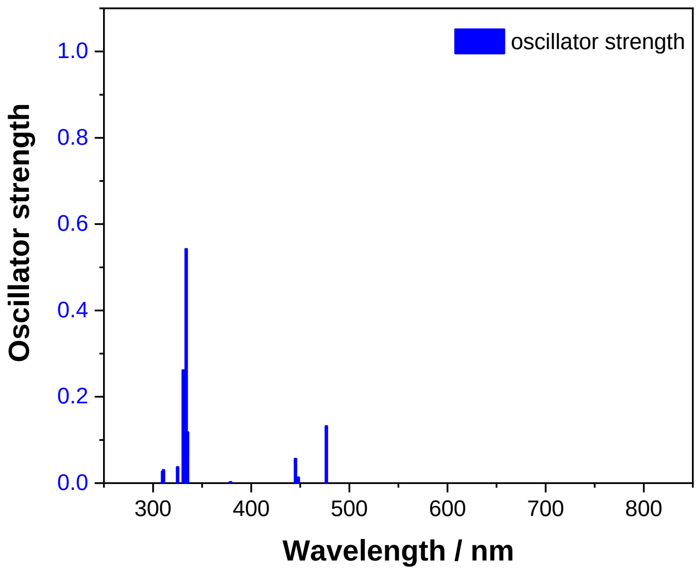

Figure S54. TD-DFT calculated singlet electronic transitions of **7'** in the gas phase using CAM-B3LYP/6-31+G (d).

Cartesian Coordinates

Compound 6 (DFT B3LYP/6-31+G(d), S_0)

Total energy: -1 413 365.49 kcal mol^{-1}

Dipole moment: 5.67 D

C	2.83459600	1.31736200	-0.03072100
C	1.42644200	1.31862100	-0.00752300
C	0.71827100	0.08526300	-0.00635400
C	1.47052800	-1.11975200	-0.01801900
C	2.85787700	-1.09591100	-0.02180100
C	3.56450600	0.12717300	-0.03228100
C	-0.71827100	0.08526300	0.00634800
C	-1.42644200	1.31862000	0.00751900
C	-2.83459600	1.31736200	0.03071800
C	-3.56450500	0.12717200	0.03228000
C	-2.85787600	-1.09591200	0.02179800
C	-1.47052700	-1.11975300	0.01801300
C	0.68125600	2.54807400	-0.00270800
C	-0.68125600	2.54807400	0.00270400
H	3.35960300	2.26740200	-0.04297000
H	3.39211600	-2.03932200	-0.01376800
H	-3.35960400	2.26740200	0.04296900
H	-3.39211500	-2.03932200	0.01376400
H	1.23193700	3.48544100	-0.00554500
H	-1.23193800	3.48544000	0.00554300
N	4.96882800	0.13029600	-0.03578000
N	-4.96882800	0.13029500	0.03578000
C	5.69409400	1.20029200	0.55810300
C	6.74142700	1.82270900	-0.13983200
C	5.39073400	1.64028600	1.84916500
C	7.46549200	2.85452700	0.44347500
H	6.98830000	1.48331700	-1.14105000
C	6.09932800	2.69238500	2.43591300
H	4.58773500	1.16081400	2.40094700
C	7.14648900	3.30198200	1.73404100
H	8.27828100	3.33774700	-0.08985900
H	5.83492200	3.01054600	3.43765900
C	5.69110900	-0.90516200	-0.69274100
C	6.79077500	-1.50164400	-0.07209800
C	5.32613500	-1.33093900	-1.98038500
C	7.52410400	-2.50201900	-0.71580700
H	7.08205800	-1.17602300	0.92188000
C	6.03511100	-2.33985000	-2.61841600
H	4.47707900	-0.86971800	-2.47561700
C	7.14257000	-2.93135500	-1.99258100
H	8.37369100	-2.94177200	-0.20663800
H	5.75327800	-2.67935600	-3.61016500
C	-5.69409400	1.20029100	-0.55810300

C	-6.74142700	1.82270900	0.13983300
C	-5.39073600	1.64028400	-1.84916600
C	-7.46549200	2.85452600	-0.44347400
H	-6.98829800	1.48331700	1.14105200
C	-6.09933100	2.69238300	-2.43591300
H	-4.58773700	1.16081200	-2.40094700
C	-7.14649100	3.30198100	-1.73403900
H	-8.27828100	3.33774600	0.08986100
H	-5.83492500	3.01054400	-3.43765900
C	-5.69110800	-0.90516300	0.69274200
C	-6.79077400	-1.50164500	0.07210000
C	-5.32613400	-1.33093800	1.98038600
C	-7.52410300	-2.50201900	0.71581000
H	-7.08205800	-1.17602500	-0.92187800
C	-6.03511000	-2.33984800	2.61841900
H	-4.47707800	-0.86971700	2.47561800
C	-7.14256900	-2.93135400	1.99258400
H	-8.37369100	-2.94177300	0.20664200
H	-5.75327600	-2.67935400	3.61016800
O	7.78061000	-3.90696200	-2.70588800
O	7.90975400	4.32895000	2.21640000
O	-7.90975600	4.32894800	-2.21639800
O	-7.78060900	-3.90696100	2.70589300
C	-8.89017400	-4.55910200	2.10554500
H	-8.59732900	-5.06896200	1.17840900
H	-9.23177200	-5.29585800	2.83413200
H	-9.70171900	-3.85035300	1.89349400
C	-7.63434100	4.81187800	-3.52316400
H	-7.77367500	4.02489700	-4.27603400
H	-8.35037400	5.61581400	-3.70022700
H	-6.61300400	5.20895700	-3.59382800
C	7.63433800	4.81188000	3.52316500
H	8.35037100	5.61581600	3.70022800
H	6.61300100	5.20895900	3.59382800
H	7.77367100	4.02489900	4.27603600
C	8.89017400	-4.55910300	-2.10554000
H	8.59733000	-5.06896200	-1.17840300
H	9.23177300	-5.29586000	-2.83412500
H	9.70172000	-3.85035300	-1.89348900
O	1.36755700	-3.49807800	-0.02473900
O	-1.36755600	-3.49807800	0.02473700
C	0.77809900	-2.43096700	-0.01132800
C	-0.77809800	-2.43096800	0.01131800

Compound 7 (DFT B3LYP/6-31+G(d), S_0)

Total energy: -1 551 680.98 kcal mol^{-1}

Dipole moment: 8.86 D

C	-2.82982100	-1.91806400	-0.06747600
C	-1.42954900	-1.92919500	-0.02591000
C	-0.71349000	-0.69563500	-0.01490800
C	-1.45443000	0.51942400	-0.03564400
C	-2.84831900	0.50158100	-0.05601400
C	-3.55287800	-0.71552700	-0.07774400
C	0.71348900	-0.69563500	0.01490600
C	1.42954700	-1.92919600	0.02591000
C	2.82981900	-1.91806600	0.06747500
C	3.55287700	-0.71552900	0.07774000
C	2.84831800	0.50157900	0.05601000
C	1.45443000	0.51942300	0.03564100
C	0.71578600	1.77252100	0.01809300
C	-0.71578600	1.77252200	-0.01809500
C	-0.68082500	-3.15845600	-0.01199700
C	0.68082200	-3.15845700	0.01199900
N	1.39721000	2.92875600	0.03626700
C	0.70911100	4.06727800	0.01838400
C	-0.70910900	4.06727900	-0.01839700
N	-1.39720900	2.92875700	-0.03627200
C	-1.45370800	5.29711700	-0.03705200
N	-2.04457300	6.29790000	-0.05013600
C	1.45371000	5.29711600	0.03705700
N	2.04457600	6.29789900	0.05014600
H	-3.36450800	-2.86227300	-0.09232700
H	-3.38509800	1.44192600	-0.05564200
H	3.36450600	-2.86227500	0.09232800
H	3.38509800	1.44192400	0.05563800
H	-1.23086000	-4.09609400	-0.02230400
H	1.23085700	-4.09609400	0.02230800
N	-4.95529700	-0.71922700	-0.11542600
N	4.95529600	-0.71922800	0.11542400
C	-5.70224000	-1.77222100	0.48094300
C	-6.77899400	-2.35225100	-0.20921000
C	-5.39815100	-2.23476400	1.76394800
C	-7.52925200	-3.36533000	0.37291100
H	-7.02713500	-1.99399500	-1.20353600
C	-6.13383400	-3.26952800	2.34908600
H	-4.57454500	-1.78709600	2.31198300
C	-7.20897200	-3.83694700	1.65485200
H	-8.36441600	-3.81563200	-0.15458100
H	-5.86768100	-3.60637600	3.34423800
C	-5.66023000	0.35866800	-0.72495000
C	-6.70354200	0.99544500	-0.05115200

C	-5.32543400	0.78748000	-2.01944300
C	-7.41089900	2.04271500	-0.64874600
H	-6.97109700	0.66623000	0.94838800
C	-6.00798700	1.84014500	-2.61314700
H	-4.51866200	0.29355200	-2.55269300
C	-7.05851500	2.47531300	-1.93267700
H	-8.21672600	2.51507100	-0.09934700
H	-5.75055000	2.18286800	-3.61041800
C	5.70224200	-1.77222000	-0.48094400
C	6.77899600	-2.35224800	0.20921100
C	5.39816000	-2.23476100	-1.76395100
C	7.52925900	-3.36532400	-0.37290800
H	7.02713300	-1.99399200	1.20353900
C	6.13384800	-3.26952200	-2.34908800
H	4.57455600	-1.78709400	-2.31198900
C	7.20898500	-3.83694000	-1.65485100
H	8.36442200	-3.81562500	0.15458600
H	5.86770000	-3.60636900	-3.34424200
C	5.66022600	0.35866900	0.72495000
C	6.70353700	0.99544700	0.05115300
C	5.32542400	0.78748100	2.01944100
C	7.41089100	2.04272000	0.64874800
H	6.97109500	0.66623300	-0.94838600
C	6.00797300	1.84014700	2.61314600
H	4.51865200	0.29355200	2.55268900
C	7.05850200	2.47531800	1.93267800
H	8.21671700	2.51507700	0.09935200
H	5.75053300	2.18287100	3.61041600
O	-7.67281100	3.49338900	-2.60388500
O	-7.99958500	-4.84331900	2.13601500
O	7.99960300	-4.84330900	-2.13601300
O	7.67279400	3.49339600	2.60388800
C	8.71133500	4.20304000	1.94304800
H	8.34427300	4.67780200	1.02398000
H	9.03855100	4.97160600	2.64470600
H	9.55498100	3.54214900	1.70397000
C	7.72551900	-5.34781700	-3.43497200
H	7.83014900	-4.56393800	-4.19662100
H	8.46576600	-6.12929400	-3.61281800
H	6.71718500	-5.77911600	-3.48984600
C	-7.72549500	-5.34782700	3.43497200
H	-8.46574100	-6.12930700	3.61282000
H	-6.71716100	-5.77912400	3.48984300
H	-7.83012600	-4.56394900	4.19662300
C	-8.71135200	4.20303100	-1.94304300
H	-8.34428700	4.67779500	-1.02397600
H	-9.03857100	4.97159600	-2.64470100
H	-9.55499600	3.54213900	-1.70396200

Compound 8 (DFT B3LYP/6-31+G(d), S_0)

Total energy: -1 532 391.68 kcal mol^{-1}

Dipole moment: 0.040 D

C	2.83204000	-1.94237400	0.06426400
C	1.42915400	-1.94974100	0.02439600
C	0.71564700	-0.71565100	0.01378400
C	1.45289500	0.49907500	0.03094400
C	2.84486800	0.47654200	0.04524200
C	3.54898400	-0.74146600	0.06831300
C	-0.71564700	-0.71565100	-0.01377900
C	-1.42915500	-1.94974100	-0.02439100
C	-2.83204000	-1.94237400	-0.06426200
C	-3.54898400	-0.74146600	-0.06831300
C	-2.84486800	0.47654300	-0.04524000
C	-1.45289500	0.49907500	-0.03093900
C	-0.72210100	1.76891800	-0.01646300
C	0.72210200	1.76891700	0.01647900
C	0.68109100	-3.17834400	0.01138000
C	-0.68109100	-3.17834400	-0.01137500
N	-1.40804000	2.90454300	-0.03520000
N	1.40804100	2.90454300	0.03519600
H	3.36636300	-2.88708800	0.08752200
H	3.38062100	1.41766400	0.03975200
H	-3.36636400	-2.88708700	-0.08752200
H	-3.38062100	1.41766400	-0.03974900
H	1.23126100	-4.11619100	0.02119100
H	-1.23126100	-4.11619100	-0.02118700
N	4.95814600	-0.74397900	0.09304600
N	-4.95814600	-0.74397900	-0.09304800
C	5.68948900	-1.77350300	-0.55807700
C	6.76834600	-2.39729400	0.09013300
C	5.36094800	-2.17870400	-1.85467800
C	7.49793800	-3.39296400	-0.54628600
H	7.03385600	-2.08770300	1.09625100
C	6.07533600	-3.19578700	-2.49455400
H	4.53192600	-1.70127000	-2.36806800
C	7.15371800	-3.80477600	-1.84222400
H	8.33364600	-3.87677100	-0.05004800
H	5.78873500	-3.48776500	-3.49823800
C	5.66881500	0.29184300	0.75702100
C	6.78006600	0.88952500	0.15727100
C	5.27800600	0.72467300	2.03551700
C	7.49941700	1.89373700	0.81209800
H	7.09308800	0.56167100	-0.82921800
C	5.97246100	1.73732500	2.68313100
H	4.41853400	0.26577200	2.51418400
C	7.09186600	2.32799100	2.07827600

H	8.35929100	2.33124300	0.31820100
H	5.67054100	2.07893700	3.66838700
C	-5.68948900	-1.77350300	0.55807700
C	-6.76834500	-2.39729500	-0.09013300
C	-5.36094800	-2.17870200	1.85467800
C	-7.49793700	-3.39296500	0.54628700
H	-7.03385500	-2.08770600	-1.09625200
C	-6.07533600	-3.19578500	2.49455500
H	-4.53192600	-1.70126700	2.36806700
C	-7.15371700	-3.80477500	1.84222600
H	-8.33364500	-3.87677200	0.05004900
H	-5.78873500	-3.48776100	3.49824000
C	-5.66881600	0.29184300	-0.75702400
C	-6.78006600	0.88952400	-0.15727300
C	-5.27800700	0.72467100	-2.03552000
C	-7.49941800	1.89373600	-0.81210100
H	-7.09308800	0.56167100	0.82921600
C	-5.97246300	1.73732300	-2.68313400
H	-4.41853500	0.26577000	-2.51418600
C	-7.09186700	2.32798900	-2.07828000
H	-8.35929200	2.33124200	-0.31820400
H	-5.67054200	2.07893400	-3.66839100
O	7.71453500	3.30935600	2.80142400
O	7.92521600	-4.79983800	-2.37873400
O	-7.92521500	-4.79983700	2.37873700
O	-7.71453700	3.30935300	-2.80142800
C	-8.84848400	3.94261200	-2.22924700
H	-8.58998700	4.45071300	-1.29062800
H	-9.18003100	4.67954300	-2.96254000
H	-9.65624100	3.22203800	-2.04358200
C	-7.62015700	-5.24795700	3.69069400
H	-7.72086200	-4.43572100	4.42282300
H	-8.34613500	-6.03126900	3.91428100
H	-6.60504300	-5.66355500	3.74392600
C	7.62015800	-5.24796000	-3.69069100
H	8.34613700	-6.03127200	-3.91427700
H	6.60504400	-5.66355900	-3.74392200
H	7.72086200	-4.43572500	-4.42282100
C	8.84848200	3.94261400	2.22924200
H	8.58998600	4.45071500	1.29062300
H	9.18002800	4.67954600	2.96253500
H	9.65624000	3.22204100	2.04357900
C	0.71676600	4.06650800	0.01906100
C	-0.71676400	4.06650800	-0.01910200
C	1.41344500	5.30515500	0.03991900
C	0.71105600	6.48877100	0.02047200
H	1.24304900	7.43602400	0.03628800
C	-0.71105800	6.48877100	-0.02043200
C	-1.41344400	5.30515500	-0.03993600
H	-1.24305100	7.43602500	-0.03621500
H	-2.49856300	5.27897700	-0.07138800

H	2.49856400	5.27897900	0.07135200

Compound 7’ (DFT B3LYP/6-31+G(d), S_0)

Total energy: 840 420.97 kcal mol^{-1}

Dipole moment: 6.17 D

C	3.58900100	-0.22699300	-0.03925000
C	2.89329800	1.01350800	0.11542100
C	1.49085300	1.01692500	0.14956100
C	0.72621500	-0.17868500	0.02145600
C	1.43671800	-1.40372200	-0.08631700
C	2.85668800	-1.42203200	-0.14512400
C	0.74280400	2.25785700	0.41173000
C	-0.72429800	-0.17475300	0.01336600
C	-1.48847400	1.03057400	-0.05695100
C	-0.75091500	2.30884200	0.01478200
C	-2.88922600	1.00924300	-0.16760600
C	-3.58667900	-0.23604600	-0.04237100
C	-2.85006300	-1.43091300	0.05141200
C	-1.43225000	-1.40760700	0.01105600
C	-5.78014000	0.94460000	-0.15581200
H	-6.73387600	0.66040800	-0.61777800
H	-6.01485200	1.36550600	0.83837700
C	-3.59346000	-2.73911400	0.20993500
H	-3.80192900	-3.18857900	-0.77358500
H	-2.98536600	-3.46182200	0.75963200
C	-4.90576100	-2.52071900	0.96115000
H	-5.47369800	-3.45474900	1.03691600
H	-4.68793900	-2.18110400	1.98176200
C	-5.73891900	-1.47368800	0.24292900
H	-6.13360100	-1.88635600	-0.70164700
H	-6.60657500	-1.19975300	0.85730400
C	-5.06286400	1.97392800	-1.00625800
H	-4.95797700	1.58530600	-2.02797600
H	-5.65545500	2.89376500	-1.06373400
C	-3.68605500	2.27268800	-0.42104000
H	-3.78760100	2.84410000	0.51369700
H	-3.13052400	2.92867300	-1.08930600
N	-4.97698400	-0.26097800	-0.02340500
C	3.56013700	-2.75490100	-0.32657800
H	3.46415500	-3.34510300	0.59708100

H	3.05735500	-3.33462400	-1.10891700
C	5.03714900	-2.60816700	-0.68777500
H	5.56309500	-3.55786200	-0.53699000
H	5.14348100	-2.33327400	-1.74426900
C	5.66122400	-1.51313600	0.16057600
H	6.72044800	-1.38538000	-0.08568500
H	5.60190700	-1.78100600	1.23313600
C	5.73808000	0.92858700	0.32030600
H	6.77092300	0.80273900	-0.02267900
H	5.76581400	1.01244100	1.42367300
C	5.10973700	2.17264200	-0.27947500
H	5.68860900	3.06166700	-0.00487600
H	5.12906400	2.08799400	-1.37357400
C	3.67660600	2.30746900	0.22547900
H	3.15639200	3.10707500	-0.30730400
H	3.68294700	2.63145800	1.27404400
N	4.99148100	-0.24854800	-0.09954600
O	1.21466600	3.25965300	0.93159600
O	-1.22727200	3.42207000	-0.16420400
C	-0.67621500	-2.62510400	-0.05209100
H	-1.19378000	-3.57612000	-0.08459000
C	0.68319300	-2.62295800	-0.12003000
H	1.20005200	-3.57246100	-0.18608600

Compound 15 (DFT B3LYP/6-31+G(d), S_0)

Total energy: 933 187.216 kcal mol^{-1}

Dipole moment: 0.40 D

C	1.1985760	2.8356160	-0.1696220
C	1.1995120	1.4425760	-0.1704550
C	0.0000030	0.7349960	-0.0000040
C	-1.1995050	1.4425800	0.1704340
C	-1.1985670	2.8356200	0.1695750
C	0.0000050	3.5331120	-0.0000300
C	0.0000030	-0.7349950	0.0000030
C	1.1995110	-1.4425780	0.1704470
C	1.1985760	-2.8356170	0.1695990
C	0.0000050	-3.5331110	-0.0000010
C	-1.1985680	-2.8356180	-0.1695970
C	-1.1995060	-1.4425780	-0.1704420
C	-2.4746270	-0.6540700	-0.4183610

C	-2.4746240	0.6540730	0.4183640
C	2.4746320	0.6540660	-0.4183690
C	2.4746290	-0.6540680	0.4183710
O	-3.6328790	1.4328790	0.1224680
O	-3.6328780	-1.4328750	-0.1224590
O	3.6328800	1.4328800	-0.1224700
O	3.6328790	-1.4328830	0.1224780
O	2.4942290	0.3725840	-1.7993200
O	2.4942200	-0.3725870	1.7993230
O	-2.4942140	0.3726060	1.7993180
O	-2.4942300	-0.3726030	-1.7993160
C	4.2357460	1.2214440	1.1608220
C	3.2098050	0.8047520	2.1958890
C	4.2357640	-1.2214350	-1.1608020
C	3.2098360	-0.8047450	-2.1958840
C	-3.2098180	-0.8047210	2.1958950
C	-4.2357530	-1.2214120	1.1608210
C	-3.2098520	0.8047130	-2.1958880
C	-4.2357750	1.2214000	-1.1608010
H	2.1368190	3.3627410	-0.3049360
H	-2.1368100	3.3627480	0.3048810
H	0.0000060	4.6196180	-0.0000410
H	2.1368190	-3.3627420	0.3049090
H	0.0000050	-4.6196170	-0.0000050
H	-2.1368100	-3.3627450	-0.3049040
H	4.6918820	2.1744360	1.4469390
H	5.0228580	0.4620790	1.0755610
H	2.4842960	1.6112470	2.3601140
H	3.7018150	0.5699040	3.1448960
H	4.6919100	-2.1744230	-1.4469170
H	5.0228700	-0.4620660	-1.0755250
H	2.4843380	-1.6112450	-2.3601280
H	3.7018610	-0.5698840	-3.1448800
H	-2.4843190	-1.6112190	2.3601410
H	-3.7018370	-0.5698500	3.1448920
H	-4.6919040	-2.1743950	1.4469470
H	-5.0228560	-0.4620390	1.0755410
H	-2.4843640	1.6112200	-2.3601460
H	-3.7018800	0.5698370	-3.1448780
H	-4.6919420	2.1743750	-1.4469240
H	-5.0228670	0.4620170	-1.0755030

References

[1] G. M. Sheldrick, *Acta Crystallogr.* **2015**, *A71*, 3.

[2] K. B. H. Putz, *Diamond*, Crystal and Molecular Structure Visualization, Crystal Impact H. Putz & K. Brandenburg GbR, Bonn (Germany), **2017**.

[3] M. J. Turner, J. J. McKinnon, S. K. Wolff, D. J. Grimwood, P. R. Spackman, D. Jayatilaka and M. A. Spackman, *CrystalExplorer17*, University of Western Australia, **2017**.

[4] C. F. Macrae, I. J. Bruno, J. A. Chisholm, P. R. Edgington, P. McCabe, E. Pidcock, L. Rodriguez-Monge, R. Taylor, J. van de Streek, P. A. Wood, *J. Appl. Crystallogr.* **2008**, *41*, 466.

[5] O. V. Dolomanov, L. J. Bourhis, R. J. Gildea, J. A. K. Howard, H. Puschmann, *J. Appl. Crystallogr.* **2009**, *42*, 339.
